# Photoswitchable glycoligands targeting *Pseudomonas aeruginosa* LecA

**DOI:** 10.3762/bjoc.20.132

**Published:** 2024-07-03

**Authors:** Yu Fan, Ahmed El Rhaz, Stéphane Maisonneuve, Emilie Gillon, Maha Fatthalla, Franck Le Bideau, Guillaume Laurent, Samir Messaoudi, Anne Imberty, Juan Xie

**Affiliations:** 1 Université Paris-Saclay, ENS Paris-Saclay, Institut d'Alembert, CNRS, Photophysique et Photochimie Supramoléculaires et Macromoléculaires, 91190, Gif-sur-Yvette, France; 2 Université Paris-Saclay, CNRS, BioCIS, 92290, Orsay, France; 3 Université Grenoble Alpes, CNRS, CERMAV, 38000 Grenoble, France; 4 Laboratoire de Synthèse Organique, Ecole Polytechnique, CNRS, ENSTA, Institut Polytechnique de Paris, 91128 Palaiseau, France

**Keywords:** carbohydrates, glycosyl azobenzenes, lectin A, photoswitchable ligands

## Abstract

Biofilm formation is one of main causes of bacterial antimicrobial resistance infections. It is known that the soluble lectins LecA and LecB, produced by *Pseudomonas aeruginosa*, play a key role in biofilm formation and lung infection. Bacterial lectins are therefore attractive targets for the development of new antibiotic-sparing anti-infective drugs. Building synthetic glycoconjugates for the inhibition and modulation of bacterial lectins have shown promising results. Light-sensitive lectin ligands could allow the modulation of lectins activity with precise spatiotemporal control. Despite the potential of photoswitchable tools, few photochromic lectin ligands have been developed. We have designed and synthesized several *O*- and *S*-galactosyl azobenzenes as photoswitchable ligands of LecA and evaluated their binding affinity with isothermal titration calorimetry. We show that the synthesized monovalent glycoligands possess excellent photophysical properties and strong affinity for targeted LecA with *K*_d_ values in the micromolar range. Analysis of the thermodynamic contribution indicates that the *Z*-azobenzene isomers have a systematically stronger favorable enthalpy contribution than the corresponding *E*-isomers, but due to stronger unfavorable entropy, they are in general of lower affinity. The validation of this proof-of-concept and the dissection of thermodynamics of binding will help for the further development of lectin ligands that can be controlled by light.

## Introduction

Bacterial infection is a growing health problem due to antimicrobial resistance (AMR) among others. AMR causes approximately 33,000 deaths per annum in Europe only [1], and costs between €1.5 and €9 billion in healthcare and associated activities. Many bacterial infections occur by adhesion to host tissues through receptor–ligand interaction between bacterial carbohydrate-binding proteins (lectins) and oligosaccharides at the host cell surface. *Pseudomonas aeruginosa* (PA), a Gram-negative, opportunistic and ubiquitous environmental bacterium, is known as the leading cause of morbidity and mortality in cystic fibrosis and immunocompromised patients and as one of the leading causes of nosocomial infections [1]. Due to the existence of numerous molecular mechanisms conferring resistance to multiple classes of antibiotics, therapeutic options are increasingly limited for treatment of infections. PA has been classified as a priority 1 pathogen by the WHO [2–3]. Various approaches to treating PA, in addition to traditional antibiotics, have been developed including inhibition of quorum sensing, biofilm formation, iron chelation, and interfering with biosynthetic pathways of the bacterium [2–3]. The soluble lectins LecA and LecB produced by PA play a key role in the infection [4]. PA LecA is demonstrated to be crucial for biofilm formation and internalization, while the extracellular LecB plays a key role in bacterial adhesion to the host and biofilm formation [5–8]. Building synthetic glycoconjugates for the inhibition and modulation of bacterial lectins responsible for biofilm formation have shown promising results [9–10]. Unlike antibiotics, lectin inhibitors could prevent pathogenicity by interfering with virulence factors instead of killing the bacteria. Bacterial lectins are therefore attractive targets for the development of new antibiotic-sparing anti-infective drugs. For example, some *Escherichia coli* fimbrial lectin FimH inhibitors are currently in clinical development to treat and prevent urinary tract infections [9–10]. A large number of glycomimetic inhibitors of PA LecA and LecB have also been reported, with antibiofilm formation activity for some of them [5–8].

Photochromic molecules, which may be reversibly converted between different isomers upon illumination, offer numerous opportunities for reversibly photomodulating chemical, biological or pharmacological activities or properties [11–12]. Light is generally noninvasive and orthogonal toward most elements of living systems. It can be easily and precisely controlled in time, location, wavelength, and intensity, thus enabling the precise activation and deactivation of biological function. It also offers the potential to change the properties of defined molecules in biological systems with minimal disturbance to the rest of the system. Photoswitchable ligands, i.e., the incorporation of light-responsive moieties into a drug-like molecular structure, allow reversible light modulation of their activity since each isomer shows distinct structural and electronic properties [13]. Photoisomerization-induced conformational and polarity changes may allow to increase or decrease the interaction with the target protein or receptors, then modulate the drug potency on/off or from low to high. This strategy can be used for specific targeting or local drug activation to reduce its toxicity [14]. There is an increasing use of the photoisomerization to control the conformation as well as the activities of various biomolecules with the development of photopharmacology [11–18]. The group of Lindhorst has reported a series of mannosyl azobenzenes targeting *E. coli* lectin FimH, demonstrating the possibility to control the type 1 fimbriae-mediated bacterial adhesion to a self-assembled monolayer of mannosyl azobenzene on a gold surface [19–20] or to mannosyl azobenzene-modified human cells [21] through photoswitching the orientation of the attached mannoside [22]. Photoswitchable glycooligomers [23] or glycodendrimers [24] have been investigated for the inhibition of PA lectin PA-IL or LecA and LecB. A variation of the IC_50_ value by a factor up to 1.6 has been observed for the divalent ligand [23]; while almost no difference of inhibition was observed for LecA and LecB upon irradiation, probably due to the low photoisomerization of glycodendrimers [24]. Very recently, the group of Wittmann reported an arylazopyrazole-linked divalent *N*-acetylglucosamine targeting lectin wheat germ agglutinin [25]. The binding affinity *K*_d_ evaluated by isothermal titration calorimetry (ITC) showed a variation by a factor of 12.5 upon photoisomerization. However, a direct photomodulation of a monovalent lectin ligand has not been achieved up to date. Based on our experiences in photoswitchable glycosides and bacterial lectins [4,6–8,26–31], we have designed, synthesized, and characterized the first generation of *O*- and *S*-galactosyl azobenzenes as photoswitchable monovalent ligands targeting PA LecA. Their binding affinity with LecA evaluated by ITC showed *K*_d_ values in the micromolar range with significant thermodynamic differences between *E*- and *Z*-azobenzene isomers, demonstrating the proof-of-concept of photomodulation of the ligand–lectin interactions.

## Results and Discussion

### Design of LecA photoswitchable ligands

**Figure 1:**
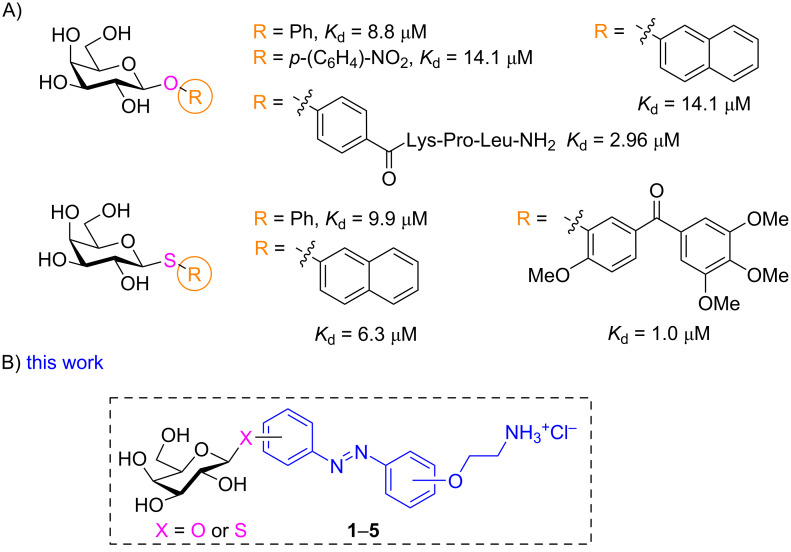
(A) Selected monovalent inhibitors for PA LecA and (B) designed general structure of photoswitchable ligands **1**–**5** targeting LecA.

The cytotoxic LecA which has a tetrameric structure, displays a high affinity for ᴅ-galactose (ᴅ-Gal, with *K*_d_ = 34 μM) and galactosides. The 3- and 4-hydroxy function on the ᴅ-Gal unit are involved in the coordination of Ca^2+^ in the binding site [5–8,32]. A large range of galactosyl conjugates have been synthetized, with *K*_d_ values from micromolar (for monovalent galactosides) to nanomolar range (for di- and multivalent derivatives) [5–8]. For the monovalent system, it has been shown that aromatic aglycons favored “T-shaped” CH^...^π interactions with the protons of the His50 imidazole in the carbohydrate-binding pocket, with the β-linked aromatic aglycons having five-fold higher affinity compared to aliphatic analogues [33–34]. Beside β-*O*-aryl galactosides, enzymatically more stable β-*S*-aryl galactosides have also been successfully developed as monovalent LecA ligands (Figure 1A) [30,35]. Since different sizes and substituents are tolerated on the aryl aglycon, we decided to replace the aryl aglycon by photoswitchable azobenzene in both *O*- and *S*-galactosides (Figure 1B) to investigate their binding affinity and the influence of the photoisomerization on the lectin interaction. The ammonium group is introduced on the azobenzene to increase the water solubility. The influence of *ortho*, *meta*, and *para*-substitution patterns of the azobenzene on the lectin binding has also been studied.

### Synthesis

**Scheme 1:**
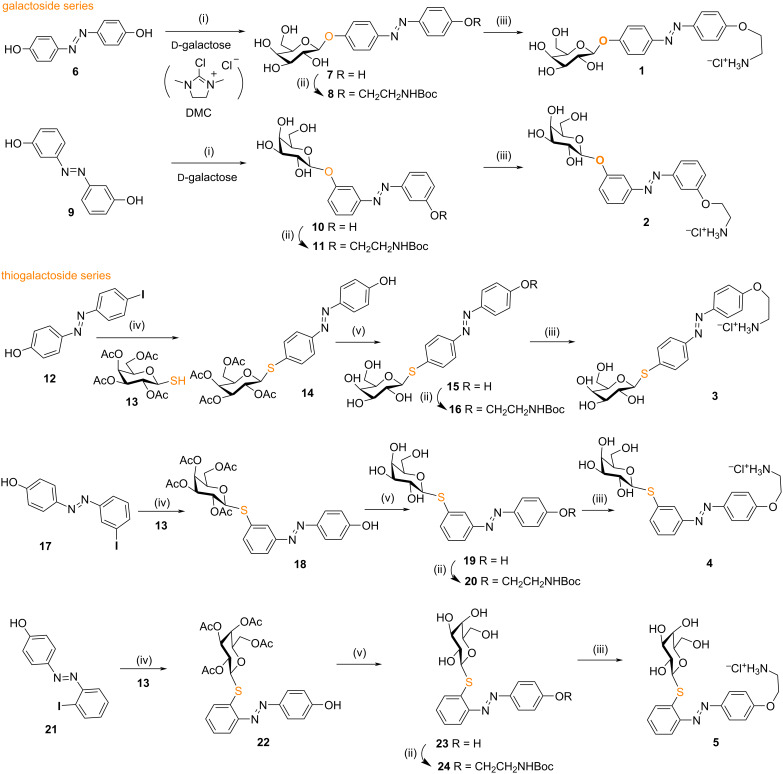
Synthesis of photoswitchable LecA inhibitors. Reagents and conditions: (i) DMC, Et_3_N, H_2_O, −10 °C to rt, 8 h, 50% for **7**, 40% for **10**; (ii) BrCH_2_CH_2_NHBoc, K_2_CO_3_, DMF, 60 °C, 15 h, 91% for **8**, 80% for **11**, 88% for **16**, 50% for **20**, 88% for **24**; (iii) AcCl, MeOH, 0 °C to rt, 15 h, 58% for **1**, 46% for **2**, 90% for **3**, 85% for **4**, 77% for **5**; (iv) Xantphos Pd-G_3_ (5 mol %), Et_3_N, THF, 6–8 h, rt, 90% for **14**, 98% for **18**, 54% for **22**; (v) MeONa/MeOH, 30 min–2 h, rt, compounds **15**, **19** and **23** were used without further purification.

The β-*O*-galactosyl *p*,*p'*-bis-substituted azobenzene derivative **1** was prepared from galactose and commercially available *p*,*p’*-dihydroxyazobenzene (**6**), by using our recently developed DMC (2-chloro-1,3-dimethylimidazolinium chloride)-mediated one-pot glycosylation method in water [28], followed by *O*-alkylation of the remaining hydroxy group with BrCH_2_CH_2_NHBoc and acidic deprotection (Scheme 1). Three equivalents of dihydroxyazobenzene **6** were used for the selective monoglycosylation step, with the excess of azobenene being recovered after column chromatography. Under these conditions, no bisglycosylated azobenzene was observed [28]. The observed 1,2-*trans* glycosylation could be explained either by the formation of the 1,2-anhydro sugar through intramolecular attack of the 2-hydroxy group of the DMC-activated β-intermediate, followed by dihydroxyazobenzene attacking the anomeric center in an S_N_2 manner, or by direct nucleophilic S_N_2 attack on the DMC-activated α-intermediate, to produce the corresponding β-*O*-galactoside [36]. The same strategy was applied for the *m*,*m’*-substituted derivative **2**, starting from the glycosylation of *m*,*m’*-dihydroxyazobenzene (**9**) [37], followed by *O*-alkylation and Boc deprotection to afford the galacoside **2** in 19% total yield. Unfortunately, all our attempts to synthesize the *o*,*o’*-bis-substituted derivative failed. For the β-*S*-galactosyl azobenzene derivatives which are accessible by our previously reported Pd-catalyzed cross-coupling methodology between glycosyl thiols and iodoaryl partners [30,38], the required *p-*, *m-* or *o*-iodo-*p’*-hydroxyazobenzenes **12**, **17**, and **21** were prepared by the diazonium coupling method according to a reported procedure [39–40]. Then the coupling with tetra-*O*-acetylated β-galactosylthiol **13** catalyzed by Xantphos Pd-G_3_ [38] as precatalyst followed by post-functionalization furnished the desired β-*S*-galactosyl azobenzenes **3**, **4**, and **5** in respectively 71%, 41%, and 37% total yields (Scheme 1).

### Photophysical characterization

**Figure 2:**
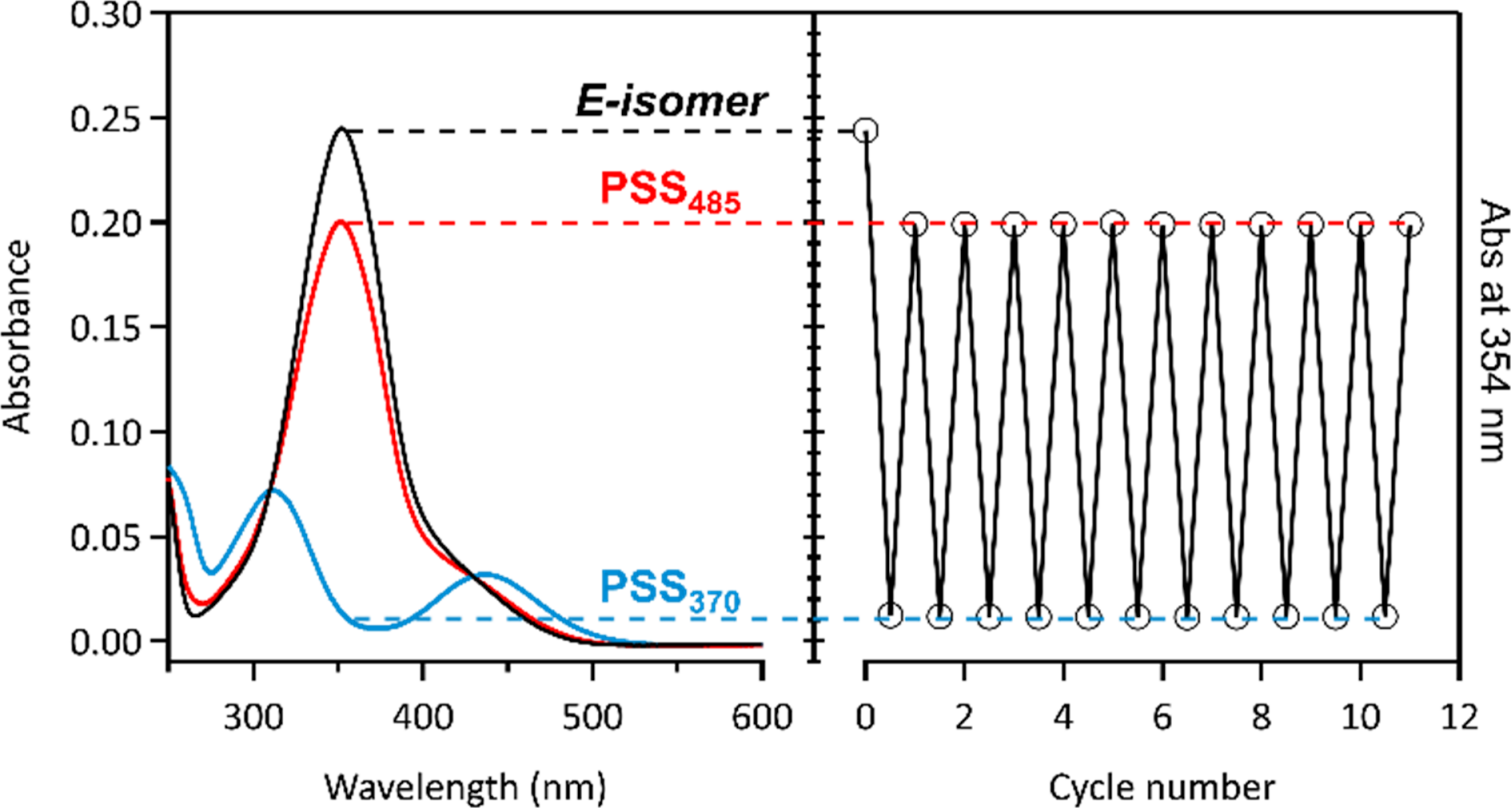
(Left) Absorption spectra and (right) fatigue resistance of **1** under alternated 370/485 nm irradiations in Tris buffer/DMSO 95:5 at rt: *E*-**1** (black line), PSS_370_ (blue line), PSS_485_ (red line). Irradiation conditions at 370 nm: 12.8 mW·cm^−2^, 20 s; at 485 nm: 1.5 mW·cm^−2^, 480 s.

**Figure 3:**
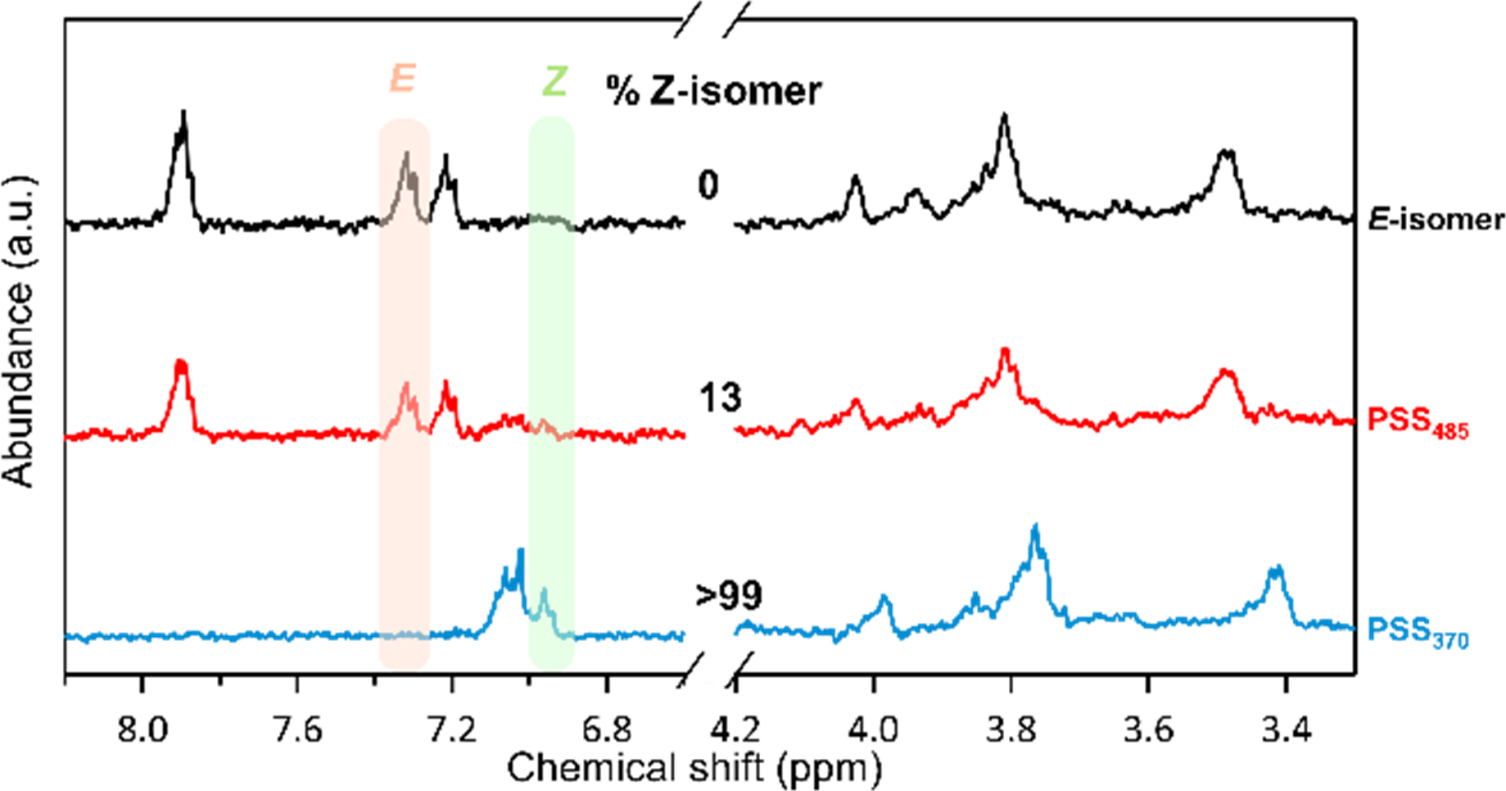
^1^H NMR (400 MHz) spectra of *E*-**1** (black line), PSS_370_ (red line), PSS_485_ (blue line) in D_2_O/DMSO-*d*_6_ 95:5.

The photoswitching properties of galactosyl azobenzenes **1**–**5** were realized in water or in Tris buffer containing 5 to 10% DMSO, in accordance with the biophysical evaluation conditions by using ITC. All these compounds undergo reversible photoisomerization under UV–vis irradiation in aqueous solution. The *O*-galactosyl azobenzene **1** shows reversible photoisomerization under UV (370 nm) and visible (485 nm) irradiations in water, with a high fatigue resistance as no degradation has been observed after more than 10 UV–vis irradiation cycles (Figure 2). According to the absorption spectra (Figure 2, black line), the *E*-isomer shows a relatively strong π→π* transition (λ_max_ = 353 nm) and a weaker forbidden n→π* transition (λ_max_ ≈ 440 nm). After irradiation at 370 nm to induce the *E*-to-*Z* photoisomerization, the band at 353 nm decreases concomitantly to the appearance of two new bands at 312 and 438 nm (Figure 2, blue line). Two isosbestic points can also be observed at 310 and 429 nm. The back *Z*→*E* photoisomerization can be achieved by illumination at 485 nm (Figure 2, red line). ^1^H NMR spectroscopy has been used to determine the *Z*/*E* ratios during irradiation, showing an excellent photoconversion yield of *Z*/*E* = 99:1 at PSS_370_, and *E*/*Z* = 87:13 at PSS_485_ in D_2_O/5% DMSO (Figure 3). As the *Z*-isomer is metastable, its half-life has been determined to be 44.4 h in water at room temperature (Figure S9 in Supporting Information File 1). All the photophysical properties of compounds **1**–**5** are summarized in Table 1 (spectra are shown in Figure S1–S24 in Supporting Information File 1). Concerning the *meta*-substituted azobenzene **2**, a 30 nm blue shift is observed for the π→π* transition (λ_max_ = 321 nm) as well as a lower absorption coefficient compared to the *para*-derivative **1** (Table 1, entries 3 and 4 vs entries 1 and 2), probably due to less-conjugated azobenzene. Compared to compound **1**, a better *Z*→*E* photoconversion was achieved with irradiation at 438 nm instead of 485 nm. Moreover, the thermostability is increased (*t*_1/2_ = 29 days). The *S*-galactosyl azobenzenes **3**–**5** also displayed excellent photoswitching properties, with a red shift for the π→π* transition (λ_max_ = 348–364 nm) compared to the *O*-galactosyl derivatives (Table 1, entries 5–8). However, the absorption coefficient and the thermostability of the *Z*-isomers are increased for the *meta*-derivative **4**, compared to the *ortho*- (**5**) and *para*-substituted **3**.

**Table 1:** Steady-state absorption, photostationary state composition, and half-life of *Z*-isomers of **1**–**5**.

Entry	Compound	Solvent	ε [M^−1^cm^−1^]	λ_max_ [nm]	*Z*/*E* PSS_370_	*E*/*Z* PSS_485_	*t*_1/2_

1	**1**	H_2_O	25632	353	99/1	87/13	44.4 h
2		Tris^a^/DMSO 5%	24400	354	99/1	87/13	n.d.^b^
3	**2**	Tris/DMSO 5%	14155	321	87/13	71/29	29.1 d^c^
4		Tris/DMSO 10%	15288	321	87/13	74/26^d^	n.d.
5	**3**	H_2_O	18111	362	99/1	73/27	30.4 h
6		Tris/DMSO 10%	16991	364	99/1	71/29	25.9 h
7	**4**	Tris/DMSO 10%	22358	348	99/1	72/28	9.0 d
8	**5**	Tris/DMSO 10%	17336	348	92/8	60/40	73.3 h

^a^Tris buffer: Tris 20 mM (pH 7.5), NaCl 100 mM, CaCl_2_ 100 μM; ^b^not determined; ^c^days; ^d^PSS_438_ for **2**.

### Biophysical evaluation by ITC

**Figure 4:**
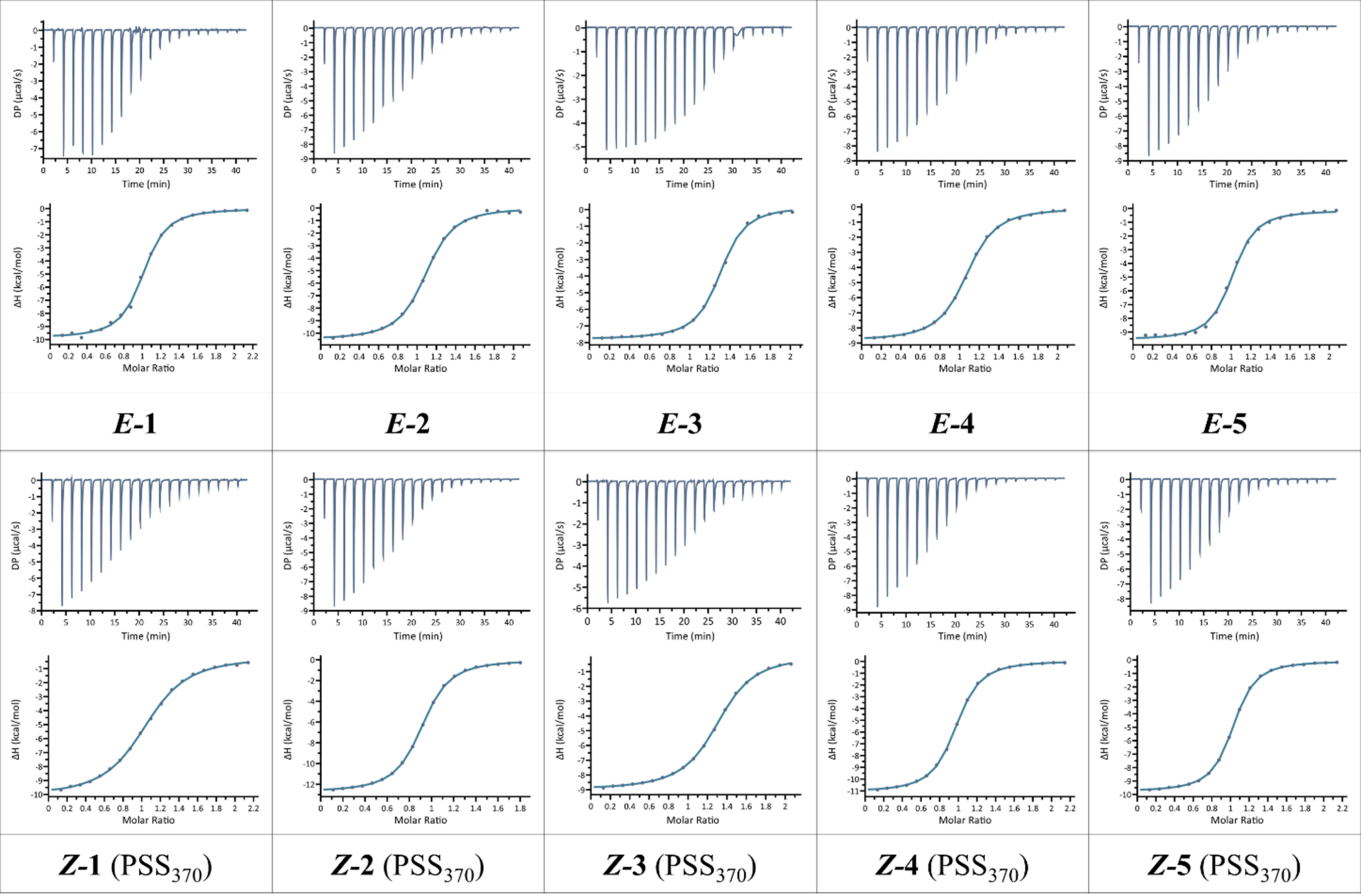
ITC titration of LecA with *E*- (up) and *Z*-isomers (bottom) of compounds **1**–**5** in Tris buffer containing 5 to 10% DMSO. The plot in the lower panel shows the total heat released as a function of total ligand concentration for the titration shown in the upper panel. The solid line represents the best least-square fit to experimental data using a one site model.

The interaction of compounds **1**–**5** with LecA was characterized by ITC analysis for both the *E-* and *Z*-isomers. As the initial isomer state of the galactosyl azobenzenes is the *E*-form, ITC measurements made on *E*-isomers correspond to 100% purity of them. After 370 nm irradiation to induce the photoizomerisation process, a photostationary state is reached between *E*- and *Z*-isomers. For ITC measurements made on *Z*-isomers, the percentage of isomers is shown in the column *Z*/*E* (PSS_370_) of Table 1. Depending on the corresponding galactosyl azobenzenes, the *Z*-isomer is pure from 87 to 99%. Spectroscopy measurements were performed on ligand solution just before each experiment to check the efficiency of the isomerization, with results as indicated in Table 2. In all experiments, strong exothermic peaks were observed for the first injection, followed by titration corresponding to stoichiometry of 1, in agreement with known structure (Figure 4). Control experiment with injection of compounds in buffer only did not show significant heat of dilution.

**Table 2:** Microcalorimetry data and thermodynamics contribution for binding to LecA. The experiments were realized in duplicate at 298 K unless otherwise stated.

Ligand	*K*_d_ [μM]	*n*	−Δ*G* [kJ/mol]	−Δ*H* [kJ/mol]	*T*Δ*S* [kJ/mol]

***E*** **-1**	4.8 ± 0.3	0.98 ± 0.01	30.3	40.8 ± 0.5	−10.5
***Z*** **-1**	13.6 ± 1.2	1.04 ± 0.04	27.8	41.3 ± 0.5	−13.5
***E*** **-2**	5.1 ± 0.7	1.01 ± 0.05	30.2	43.3 ± 1.0	−13.1
***Z*** **-2** ^a^	5.1 ± 0.7	0.97 ± 0.04	30.2	45.8 ± 0.6	−15.6
***E*** **-3**	1.9 ± 0.1	1^b^	32.6	43.5 ± 0.4	−10.9
***Z*** **-3**	4.1 ± 0.02	1^b^	30.7	49.4 ± 0.4	−18.7
***E*** **-4**	7.7 ± 1.3	0.96 ± 0.07	29.2	38.0 ± 1.3	−8.8
***Z*** **-4**	5.1 ± 0.1	0.96 ± 0.02	30.2	47.1 ± 0.2	−16.9
***E*** **-5**	4.3 ± 0.2	1.02 ± 0.05	30.6	40.1 ± 0.6	−9.5
***Z*** **-5** ^a^	4.1 ± 0	0.96 ± 0.03	30.8	41.1 ± 0.4	−10.3

^a^*Z*-isomer of compound **2** is mixed with 13% *E*-isomer and compound **5** is mixed with 8% *E*-isomer as established by PSS_370._ This contamination is less than 2% for the other compounds. ^b^Concentration of compound **3** could not be determined from weight products due to aggregation. Active concentration was determined fitting ITC data to stoichiometry of 1, value confirmed from other compounds. For all other compounds, the concentration was calculated from weighted compound and confirmed by spectroscopy (see Table S1 in Supporting Information File 1).

Affinity values, as well as thermodynamics contribution could be extracted through fitting procedure with a one site model and the data are reported in Table 2. All compounds have a strong affinity for LecA with *K*_d_ values ranging from 1.9 μM to 13.6 μM. These values are in the range of those observed previously for aromatic galactoside derivatives [33–34], confirming the favorable interaction of the aryl group with the protein surface. For all compounds, no significant differences of affinities are observed between the *E*- and *Z*-isomer, with the exception of compounds **1** and **3** with *para*-orientation between the two aryl groups. The affinity of the *E*-isomer is twice better than for its *Z*-counterpart for the *S*-linked compound **3** and three times better for the *O*-glycoside **1**. Even though the other compounds do not exhibit significant variations of affinities between *E-* and *Z*-isomers, a closer look at the thermodynamic values indicates that the mechanisms of binding display significant variations (Table 2). All of the *Z*-isomers display stronger favorable enthalpy of binding, i.e., a more negative Δ*H* contribution (Δ*H* varying from −41.1 to −49.4 kJ/mol) than their *E*-counterpart (Δ*H* from −38.0 to −43.5 kJ/mol). This is fully counterbalanced by a stronger unfavorable entropy barrier, i.e. a more positive entropy contribution (−*T*ΔS), varying from 10.3 to 18.5 kJ/mol for the *Z*-isomers, and from 8.8 to 13.1 kJ/mol for the *E-*isomers. As displayed in Figure 4, this enthalpy–entropy compensation results in a limited variation of Δ*G* and therefore in the observed rather similar *K*_d_ values.

**Figure 5:**
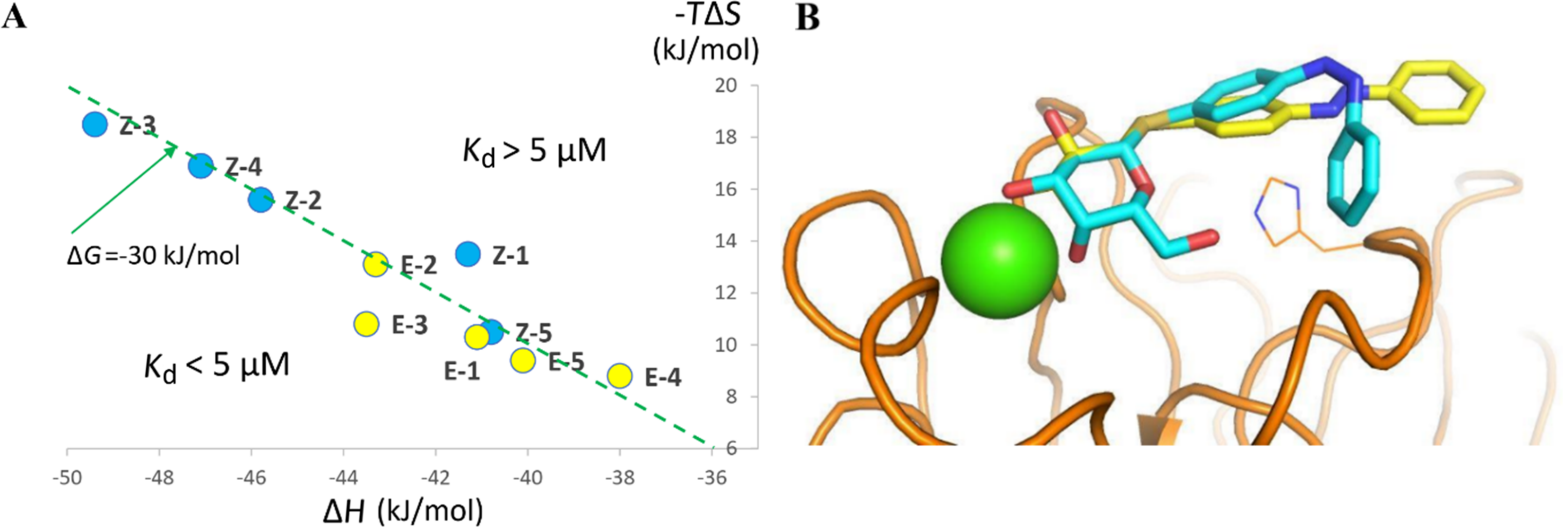
(A) Enthalpy–entropy compensation plot of compounds **1**–**5** from ITC analysis. The dotted green line represents a Δ*G* value of −30 kJ/mol, corresponding to a *K*_d_ of approx 5 μM in the experimental conditions. (B) Manual docking of scaffold for compound **3** with selected low energy conformations of the *E*-isomer (yellow sticks) and *Z*-isomer (cyan sticks) superimposed on conserved position of galactose in all LecA crystalline complexes. The protein is represented by orange ribbon, His53 by lines, and calcium by green sphere.

In order to rationalize this difference in binding mechanism, molecular models were obtained for selected low-energy conformations of *E-* and *Z*-isomers of a “model” scaffold of the *para*-azobenzene derivative in the binding site of LecA (Figure 5), by simple superpositioning of the known crystal structure. The extended *E*-isomer establishes contact through galactoside and the first aryl ring only, while the bent *Z*-isomer has proper conformation to wrap around the central His53 residue and to establish a more extended interaction with the protein surface. This would be in agreement with a stronger enthalpy of interaction, while the entropy barrier could arise from a limitation of flexibility and/or blocking of water molecules at the new interface.

## Conclusion

We have designed and synthesized in three to five steps *O*- and *S*-galactosyl azobenzenes targeting the *Pseudomonas aeruginosa* lectin LecA. The five synthesized glycoconjugates can be reversibly photoconverted between *E-* and *Z*-isomers under UV and vis irradiation, with good to excellent photoconversion yields and high fatigue resistance in aqueous media. Furthermore, all the Z-isomers displayed good thermostability, with the half-live varied from 26 h to 29 days at room temperature depending on the type of glycosidic linkage and the substitution pattern on the azobenzene moiety. The bistability of the azobenzene derivatives is suitable for the investigation of azobenzene isomers on the binding affinity with LecA. All the galactosyl azobenzenes bound to LecA in the low micromolar range. Interestingly, the *para*-substituted *O*- (**1**) and *S*- (**3**) galactosides displayed 2 to 3-fold difference in affinity between *E-* and *Z*-isomers (3-fold difference for **1** and 2-fold for **3**), demonstrating the proof-of-concept of tuning the LecA binding by light. Few differences were observed for the *meta*- (**2** and **4**) and *ortho*-substituted azobenzenes (**5**). Thermodynamics contributions exhibit larger variations with stronger enthalpy of binding for the *Z*-isomer, probably in relation with a folded conformation generating additional contact with the surface. Due to enthalpy–entropy compensation, that is a general effect in protein–carbohydrate interactions [41], this does not reflect in differences in affinity. However, these observations, together with future modeling studies, will help in designing new compounds with more selective binding of one isomer only.

## Experimental

**General experimental details.** Commercially available solvents and reagents were used without further purification. Reactions carried out under anhydrous conditions are performed under argon in glassware previously dried in an oven. DMF and THF were previously dried through alumina or molecular sieves-containing cartridges using a solvent purificator MBRAUN SPS-800. All the reactions with azobenzene-containing substrates were carried out in the dark. Reactions were monitored by TLC on Silica Gel 60F-254 plates with detection by UV (254 nm or 365 nm) or by spraying with 10% H_2_SO_4_ in EtOH and heating about 30 s at 400–600 °C. Column chromatography purification was performed on CombiFlash^®^ Rf+ and RediSep^®^ RF or RF Gold normal phase silica columns (with UV detection at 254 and 350 nm for all azobenzene derivatives), or by flash column chromatography employing silica gel (60 Å pore size, 40–63 µm). ^1^H and ^13^C NMR spectra were recorded on a JEOL ECS-400 spectrometer or on Bruker Avance 300 and 400 spectrometers. Structural assignments were made with additional information from gCOSY, HMBC, and gHMQC experiments. High-resolution mass spectra (HRMS) were performed on a Bruker maXis mass spectrometer by the SALSA platform from ICOA laboratory or on an Agilent 1260 Infinity system with a quadrupole time-of-light (Q-TOF) mass analyzer. Melting points were measured with a Köfler bench previously calibrated using the usual standard references or on a digital melting point capillary apparatus. Specific optical rotations were measured in solution using sodium light at 589 nm where no absorption occurred for all compounds. Absorption spectra were recorded on a Cary-5000 spectrophotometer from Agilent Technologies. Photochromic reactions were induced in situ by a continuous irradiation Hg/Xe lamp (Hamamatsu, LC6- or LC8-Lightningcure, 200 W) equipped with narrow band interference filters of appropriate wavelengths: Semrock BP-370/36 for λ_irr_ = 370 nm, Semrock FF01-438/24-25 for λ_irr_ = 438 nm, Semrock FF01-485/20-25 for λ_irr_ = 485 nm. The irradiation power was measured using a photodiode from Ophir (PD300-UV) and corrected after a measurement with an additional Schott long pass filter (LP-545) to measure NIR contribution (P_LP_) that is let through the Semrock filter (P_Total_), considering a 90% transmittance: P_λirr_ = P_Total_ − (10/9 × P_LP_). The photoconversion reaction was followed by a combination of ^1^H NMR and UV–vis absorption spectra, realized by successive irradiations at 370 nm (438 or 485 nm). The *E*/*Z* ratios were determined by integration of the azobenzene proton signals of each isomer. A quartz cell of 10 mm path length has been used for solution measurement.

The photoconversion yields were measured from a solution of the compounds in deuterated solvent and monitored by ^1^H NMR and UV–vis absorption, after successive irradiations at 370 nm (438 nm or 485 nm) in the case of the PSS. The *E/Z* ratios were determined by integration of characteristic of each isomer.

Data processing of spectroscopic measurements was realized with the help of Microsoft^®^ Excel^®^ and Igor Pro from WaveMetrics, Inc (versions 7 to 9).

**Isothermal titration calorimetry:** LecA was expressed and purified as previously described [42]. All experiments were performed at 25 °C with an ITC200 isothermal titration calorimeter (Microcal-Malvern Panalytical, Grenoble, France). The lyophilized LecA protein was dissolved in a buffer composed of 20 mM Tris^.^HCl (pH 7.5), 100 mM NaCl and 100 μM CaCl_2_ with 5% or 10% DMSO final. All compounds were first dissolved in DMSO then in same buffer for a final concentration of 5% or 10% DMSO. The 200 μL sample cell containing LecA (concentrations ranging from 200 to 300 μM) was subjected to injections of ligand solution: 20 injections of 2 μL (2–3 mM, depending on the ligand) at intervals of 120 s while stirring at 850 rpm. Control experiments were performed by repeating the same protocol, but injecting the ligand into buffer solution. The supplied software Origin 7 or MicroCal PEAQ-ITC was used to fit the experimental data to a theoretical titration curve allowing the determination of affinity (i.e., dissociation constant, *K*_d_), binding enthalpy (Δ*H*), and stoichiometry (*n*). Values for free energy change (Δ*G*) and entropy contributions (*T*Δ*S*) were derived from the equation Δ*G* = Δ*H* − *T*Δ*S* = − *RT* ln *K*_d_ (with *T* = 298.15 K and *R* = 8.314 J mol^−1^K^−1^).

**General procedure I for the *O*-alkylation with BrCH_2_CH_2_NHBoc:** A solution of glycosyl azobenzene (1.0 equiv) in anhydrous DMF (≈3.5 mL per mmol) was added K_2_CO_3_ (2.0–4 equiv) and BocNHCH_2_CH_2_Br (1.5–4 equiv), then stirred overnight at 60 °C. After the reaction was completed (TLC monitoring), the mixture was evaporated to dryness under reduced pressure. The residue was dissolved in EtOAc, neutralized with HCl (1 M), and extracted with EtOAc (3 times). The organic phase was washed with brine, dried over anhydrous Na_2_SO_4_, evaporated under reduced pressure in vacuo, and purified by CombiFlash Rf+ (CH_2_Cl_2_/MeOH 15:1).

**General procedure II for the Boc deprotection**: To a solution of the Boc-protected compound in anhydrous MeOH (≈10 mL per mmol) was added dropwise AcCl (1.0–3.0 equiv) at 0 °C, slowly warmed to rt, and stirred overnight. After the reaction was completed (TLC monitoring), the mixture was evaporated to dryness under reduced pressure. The residue was dissolved in MeOH, acetone was added, and a precipitate was obtained, which was washed with CH_2_Cl_2_ and *n*-pentane successively to give a pure compound.

**General procedure III for the syhthesis of *S*-galactosyl azobenzenes:** A round-bottomed flask was charged with Xantphos Pd-G_3_ (5 mol %), acetylated β-thiogalactoside **13** [38] (1.1 equiv), and iodinated azobenzene (1 equiv). After Ar flushing, dry THF (0.25 M) was added and the mixture stirred for 5 min before NEt_3_ (1.1 equiv) was added. The reaction mixture was stirred at rt under Ar for 6–8 h, diluted with EtOAc, filtered over celite, and washed with EtOAc. The collected organic layers were concentrated under reduced pressure and purified by flash chromatography (cyclohexane/EtOAc 7:3) to give the thioglycoside.

**General procedure IV for the Zemplén deacetylation:** To a seal tube containing the galactose derivatives in dry MeOH (0.15 M), NaOMe (30 mol %, 0.5 M sol. in MeOH) was added. The mixture was stirred at room temperature until total deprotection. The solution was neutralized using Amberlite IR-120 (H), filtered, concentrated and the crude material used without further purification to give the desired product in quantitative yield.

## Supporting Information

File 1 Compound characterization, detailed photochemical and photophysical procedures and copies of spectra.

## Data Availability

All data that supports the findings of this study is available in the published article and/or the supporting information to this article.
