# Supplementary material for: Photoswitchable glycoligands targeting Pseudomonas aeruginosa LecA
Source: Beilstein J Org Chem. 2024 Jul 3;20:1486–96. doi: 10.3762/bjoc.20.132 (PMC11228623; doi:10.3762/bjoc.20.132)

BEILSTEIN JOURNAL OF ORGANIC CHEMISTRY

Supporting Information

for

Photoswitchable glycoligands targeting *Pseudomonas aeruginosa* LecA

Yu Fan, Ahmed El Rhaz, Stéphane Maisonneuve, Emilie Gillon, Maha Fatthalla, Franck Le Bideau, Guillaume Laurent, Samir Messaoudi, Anne Imberty and Juan Xie

Beilstein J. Org. Chem. **2024,** *20,* 1486–1496. doi:10.3762/bjoc.20.132

Compound characterization, detailed photochemical and photophysical procedures and copies of spectra

License and Terms: This is a supporting information file under the terms of the Creative Commons Attribution License (https://creativecommons.org/licenses/by/4.0). Please note that the reuse, redistribution and reproduction in particular requires that the author(s) and source are credited and that individual graphics may be subject to special legal provisions.
The license is subject to the *Beilstein Journal of Organic Chemistry* terms and conditions: (https://www.beilstein-journals.org/bjoc/terms)

Table of contents

Characterization data S3

References S7

Detailed photochemical and photophysical procedures S8

Determination of the molar absorption coefficients (ε) S8

Determination of the half-lifes S11

General procedure for the fatigue resistance measurements S15

General procedure for the determination of the photoconvertion yields by absorption and ^{1}H NMR S17

Concentration of ligands determined by spectroscopy S23

Copies of ^{1}H and ^{13}C{^{1}H} NMR spectra S25

^{1}H NMR of compound E-**1** in CD_3OD at 400 MHz S25

Enlarged ^{1}H NMR of compound E-**1** in CD_3OD at 400 MHz S25

^{13}C{^{1}H} NMR (UDEFT) of compound E-**1** in CD_3OD at 100 MHz S26

^{1}H NMR of compound E-**2** in CD_3OD at 400 MHz S27

Enlarged ^{1}H NMR of compound E-**2** in CD_3OD at 400 MHz S27

^{13}C{^{1}H} NMR of compound E-**2** in CD_3OD at 100 MHz S28

^{1}H NMR of compound E-**3** in $CDCl_3$ at 400 MHz S29

Enlarged ^{1}H NMR of compound E-**3** in $CDCl_3$ at 400 MHz S29

^{13}C{^{1}H} NMR (UDEFT) of compound E-**3** in $CDCl_3$ at 100 MHz S30

^{1}H NMR of compound E-**4** in CD_3OD at 400 MHz S31

Enlarged ^{1}H NMR of compound E-**4** in CD_3OD at 400 MHz S31

^{13}C{^{1}H} NMR of compound E-**4** in CD_3OD at 100 MHz S32

^{1}H NMR of compound E-**5** in CD_3OD at 400 MHz S33

Enlarged ^{1}H NMR of compound E-**5** in CD_3OD at 400 MHz S33

^{13}C{^{1}H} NMR of compound E-**5** in CD_3OD at 100 MHz S34

^{1}H NMR of compound E-**8** in CD_3OD at 400 MHz S35

Enlarged ^{1}H NMR of compound E-**8** in CD_3OD at 400 MHz S35

^{13}C{^{1}H} NMR (UDEFT) of compound E-**8** in CD_3OD at 100 MHz S36

^{1}H NMR of compound E-**10** in CD_3OD at 400 MHz S37

Enlarged ^{1}H NMR of compound E-**10** in CD_3OD at 400 MHz S37

^{13}C{^{1}H} NMR of compound E-**10** in CD_3OD at 100 MHz S38

^{1}H NMR of compound E-**11** in CD_3OD at 400 MHz S39

Enlarged ^{1}H NMR of compound E-**11** in CD_3OD at 400 MHz S39

^{13}C{^{1}H} NMR of compound E-**11** in CD_3OD at 100 MHz S40

^{1}H NMR of compound E-**14** in CD_3OD at 400 MHz S41

^{13}C{^{1}H} NMR of compound E-**14** in CD_3OD at 100 MHz S41

^{1}H NMR of compound E-**15** in CD_3OD at 300 MHz S42

$^{13}C\{^{1}H\}$ NMR of compound E-**15** in $CDCl_3$ at 75 MHz S42
^{1}H NMR of compound E-**16** in CD_3OD at 400 MHz S43
Enlarged ^{1}H NMR of compound E-**16** in CD_3OD at 400 MHz S43
$^{13}C\{^{1}H\}$ NMR of compound E-**16** in CD_3OD at 100 MHz S44
^{1}H NMR of compound E-**18** in $CDCl_3$ at 300 MHz S45
$^{13}C\{^{1}H\}$ NMR of compound E-**18** in $CDCl_3$ at 75 MHz S45
^{1}H NMR of compound E-**19** in CD_3OD at 300 MHz S46
$^{13}C\{^{1}H\}$ NMR of compound E-**19** in acetone-d_6 at 75 MHz S46
^{1}H NMR of compound E-**20** in CD_3OD at 400 MHz S47
Enlarged ^{1}H NMR of compound E-**20** in CD_3OD at 400 MHz S47
$^{13}C\{^{1}H\}$ NMR of compound E-**20** in CD_3OD at 100 MHz S48
^{1}H NMR of compound E-**22** in $CDCl_3$ at 300 MHz S49
$^{13}C\{^{1}H\}$ NMR of compound E-**22** in $CDCl_3$ at 75 MHz S49
^{1}H NMR of compound E-**23** in CD_3OD at 400 MHz S50
$^{13}C\{^{1}H\}$ NMR of compound E-**23** in CD_3OD at 75 MHz S50
^{1}H NMR of compound E-**24** in CD_3OD at 400 MHz S51
Enlarged ^{1}H NMR of compound E-**24** in CD_3OD at 400 MHz S51
$^{13}C\{^{1}H\}$ NMR of compound E-**24** in CD_3OD at 100 MHz S52

Characterization data

(E)-4-[4′-(2-*tert*-Butyloxycarbonylaminoethyloxy)phenylazo)phenyl-β-D-galactopyranoside **(*E*-8):** From **7** [26] (100 mg, 0.26 mmol) and $BocNHCH_2CH_2Br$ (87 mg, 0.39 mmol), K_2CO_3 (72 mg, 0.52 mmol) according to the General procedure I, compound **8** (123 mg, 91%) was isolated as a yellow solid. R_f = 0.5 (CH_2Cl_2/MeOH = 5/1), mp: 132°C, $[\alpha]_D^{23}$: -28.6 (c = 0.5, MeOH); ^{1}H NMR (400 MHz, CD_3OD): δ 7.85 (d, J = 9.2 Hz, 2H, H_{Ph}), 7.83 (d, J = 9.2 Hz, 2H, H_{Ph}), 7.23 (d, J = 9.2 Hz, 2H, H_{Ph}), 7.07 (d, J = 8.8 Hz, 2H, H_{Ph}), 4.97 (d, J = 8.0 Hz, 1H, H_1), 4.09 (t, J = 5.6 Hz, 2H, OCH_2), 3.92 (d, J = 3.2 Hz, 1H, H_4), 3.83 (dd, J = 10.0, 7.6 Hz, 1H, H_6), 3.81-3.71 (m, 3H, $H_{2,5,6'}$), 3.61 (dd, J = 9.6, 3.2 Hz, 1H, H_3), 3.46 (t, J = 6.0 Hz, 2H, NCH_2), 1.45 (s, 9H, 3×CH_3); ^{13}C NMR (100 MHz, CD_3OD): δ 162.6, 161.1, 149.3, 148.4 (Cq); 125.4, 125.1, 118.0, 115.9 (CH_{Ph}); 102.6 (C_1); 77.1 ($C_{2\text{ or }5}$); 74.8 (C_3); 72.2 ($C_{5\text{ or }2}$); 70.2 (C_4); 68.3 (OCH_2); 62.4 (C_6); 40.9 (NCH_2); 28.7 (CH_3). HRMS (ESI) *m/z*: Calcd for $C_{25}H_{34}N_3O_9$ $[M+H]^+$: 520.2290, Found 520.2281.

(E)-4-[4′-(2-Aminoethyloxy)phenylazo)phenyl-β-D-galactopyranoside·HCl **(*E*-1):** From **8** (70 mg, 0.13 mmol) and AcCl (11 mg, 0.13 mmol) according to the General procedure II, compound **1** (34 mg, 58%) was isolated as a yellow solid. mp: 226°C, $[\alpha]_D^{23}$: -49.0 (c = 0.2, H_2O); ^{1}H NMR (400 MHz, CD_3OD): δ 7.90 (d, J = 7.2 Hz, 2H, H_{Ph}), 7.85 (d, J = 8.8 Hz, 2H, H_{Ph}), 7.24 (d, J = 9.2 Hz, 2H, H_{Ph}), 7.16 (d, J = 9.2 Hz, 2H, H_{Ph}), 4.98 (d, J = 7.6 Hz, 1H, H_1), 4.32 (t, J = 4.8 Hz, 2H, OCH_2), 3.92 (d, J = 3.2 Hz, 1H, H_4), 3.85 (dd, J = 9.6, 7.6 Hz, 1H, H_6), 3.81-3.74 (m, 3H, $H_{2,5,6}$), 3.61 (dd, J = 10.0, 3.6 Hz, 1H, H_3), 3.40 (t, J = 5.2 Hz, 2H, NCH_2); ^{13}C NMR (100 MHz, CD_3OD): δ 161.5, 161.3, 149.2, 148.9 (Cq); 125.5, 125.2, 118.0, 116.0 (CH_{Ph}); 102.6 (C_1); 77.1 ($C_{2\text{ or }5}$); 74.8 (C_3); 72.2 ($C_{5\text{ or }2}$); 70.2 (C_4); 65.6 (OCH_2); 62.4 (C_6); 40.3 (NCH_2); HRMS (ESI) *m/z*: Calcd for $C_{20}H_{26}N_3O_7$ $[M+H]^+$: 420.1765, Found 420.1763.

(E)-3-(3′-Hydroxy-phenylazo)phenyl-β-D-galactopyranoside **(*E*-10):** To a solution of D-galactose (500 mg, 2.77 mmol) in water (20 mL) was added 3,3’-dihydroxyazobenzene **9**[3] (1.78 g, 8.33 mmol, 3.0 equiv.) and Et_3N (10.4 mL, 74.79 mmol) at -10 °C. After stirring for 10 min, DMC (2.8 g, 16.60 mmol) was added and stirred for another 2 h. Et_3N (10.4 mL) was added again at -10°C and stirred for 10 min, then DMC (2.8 g) was added and stirred for 2 h. after repeating one again the addition of Et_3N and DMC, and stirred for 2 h, the reaction was completed as monitored by TLC. Aqueous NH_4OH solution (3×10 mL) was added and co-evaporated to remove $Et_3HN^+Cl^-$ at 50 °C. The residue was further purified by CombiFlash Rf+ (eluted with EtOAc/MeOH = 15/1) to afford the compound **10** as an orange solid (416 mg, 40%), along with 1.42 g of recovered azobenzene **9** (80%). R_f = 0.43 (CH_2Cl_2/MeOH = 5/1), mp: 78°C, $[\alpha]_D^{23}$: -60.8 (c = 1.0, MeOH); ^{1}H NMR (400 MHz, CD_3OD): δ 7.64 (t, J = 2.4 Hz, 1H, H_{Ph}), 7.58 (dt, J = 8.4, 0.8 Hz, 1H, H_{Ph}), 7.46 (t, J = 8.0 Hz, 1H, H_{Ph}), 7.42 (dt, J = 8.0, 1.6 Hz, 1H, H_{Ph}), 7.35 (t, J = 7.6 Hz, 1H, H_{Ph}), 7.30 (t, J = 2.4 Hz, 1H, H_{Ph}), 7.26 (ddd, J = 8.0, 2.0, 0.4 Hz, 1H, H_{Ph}), 6.94 (ddd, J = 8.0, 2.4, 0.6 Hz, 1H, H_{Ph}), 4.97 (d, J = 7.6 Hz, 1H, H_1), 3.92 (d, J = 3.6 Hz, 1H, H_4), 3.85 (dd, J = 9.6, 8.0 Hz, 1H, H_6), 3.82-3.73 (m, 3H, $H_{2,5,6'}$), 3.61 (dd, J = 10.0, 3.6 Hz, 1H, H_3); ^{13}C NMR (100 MHz, CD_3OD): δ 159.9, 159.4, 155.1, 155.0 (Cq); 130.9, 120.6, 119.5, 118.9, 116.8, 110.8, 108.9 (CH_{Ph}); 103.0 (C_1); 77.0 ($C_{2\text{ or }5}$); 74.8 (C_3); 72.2 ($C_{2\text{ or }5}$); 70.2 (C_4); 62.4 (C_6); HRMS (ESI) *m/z*: Calcd for $C_{18}H_{21}N_2O_7$ $[M+H]^+$: 377.1343, Found 377.1341.

(E)-3-[3′-(2-*tert*-Butyloxycarbonylaminoethyloxy)phenylazo)phenyl-β-D-galactopyranoside **(*E*-11):** From **10** (200 mg, 0.53 mmol) and $BocNHCH_2CH_2Br$ (475 mg, 2.12 mmol), K_2CO_3 (293 mg, 2.12 mmol) according to the General procedure I, compound **11** (220 mg, 80%) was isolated as an orange solid. R_f = 0.5 (CH_2Cl_2/MeOH = 5/1), mp: 90°C, $[\alpha]_D^{23}$: -29.0 (c = 0.5 ,

MeOH); ^{1}H NMR (400 MHz, CD_3OD): δ 7.66 (t, *J* = 2.4 Hz, 1H, H_{Ph}), 7.60 (ddd, *J* = 8.0, 1.6, 0.8 Hz, 1H, H_{Ph}), 7.53 (d, *J* = 8.4 Hz, 1H, H_{Ph}), 7.48-7.42 (m, 3H, H_{Ph}), 7.27 (ddd, *J* = 8.4, 2.4, 0.8 Hz, 1H, H_{Ph}), 7.10 (ddd, *J* = 8.0, 2.4, 0.8 Hz, 1H, H_{Ph}), 4.97 (d, *J* = 7.6 Hz, 1H, H_1), 4.09 (t, *J* = 5.2 Hz, 2H, OCH_2), 3.93 (d, *J* = 3.2 Hz, 1H, H_4), 3.88-3.70 (m, 4H, $H_{2,5,6,6'}$), 3.62 (dd, *J* = 9.6, 3.2 Hz, 1H, H_3), 3.47 (t, *J* = 5.2 Hz, 2H, NCH_2), 1.44 (s, 9H, 3×CH_3); ^{13}C NMR (100 MHz, CD_3OD): δ 160.9, 160.0, 158.5, 154.9 (Cq); 131.0, 130.9, 120.8, 119.1, 119.0, 118.2, 110.9, 107.8 (CH_{Ph}); 102.9 (C_1); 80.2 (Cq); 77.0 ($C_{2\ or\ 5}$); 74.7 (C_3); 72.2 ($C_{5\ or\ 2}$); 70.1 (C_4); 68.2 (OCH_2); 62.3 (C_6); 41.0 (NCH_2); 28.7 (CH_3); HRMS (ESI) *m/z*: Calcd for $C_{25}H_{34}N_3O_9$ $[M+H]^+$: 520.2290, Found 520.2286.

(*E*)-3-[3′-(2-Aminoethyloxy)phenylazo)phenyl-β-D-galactopyranoside·HCl **(*E*-2):** From **11** (200 mg, 0.38 mmol) and AcCl (45 mg, 0.57 mmol) according to the General procedure II, compound **2** (80 mg, 46%) was isolated as a yellow solid, along with 93 mg of recovered compound **11** (47%). mp: 140°C, $[\alpha]_D^{23}$: -46.1 (*c* = 0.5, H_2O); ^{1}H NMR (400 MHz, CD_3OD): δ 7.67 (t, *J* = 1.6 Hz, 1H, H_{Ph}), 7.63-7.60 (m, 2H, H_{Ph}), 7.55-7.46 (m, 3H, H_{Ph}), 7.29 (ddd, *J* = 8.4, 2.4, 0.4 Hz, 1H, H_{Ph}), 7.20 (ddd, *J* = 8.4, 2.4, 0.6 Hz, 1H, H_{Ph}), 4.97 (d, *J* = 7.6 Hz, 1H, H_1), 4.33 (t, *J* = 4.8 Hz, 2H, OCH_2), 3.93 (d, *J* = 3.2 Hz, 1H, H_4), 3.87-3.73 (m, 4H, $H_{2,5,6,6'}$), 3.61 (dd, *J* = 9.6, 3.2 Hz, 1H, H_3), 3.41 (t, *J* = 5.2 Hz, 2H, NCH_2); ^{13}C NMR (100 MHz, CD_3OD): δ 160.2, 160.0, 155.1, 154.9 (Cq); 131.3, 131.0, 120.9, 119.2, 119.1, 119.0, 110.9, 107.8 (CH_{Ph}); 103.0 (C_1); 77.1 ($C_{2\ or\ 5}$); 74.8 (C_3); 72.3 ($C_{5\ or\ 2}$); 70.2 (C_4); 65.5 (OCH_2); 62.4 (C_6); 40.3 (NCH_2); HRMS (ESI) *m/z*: Calcd for $C_{20}H_{26}N_3O_7$ $[M+H]^+$: 420.1765, Found 420.1762.

(*E*)-4-(4′-Hydroxyphenylazo)phenyl-2,3,4,6-*tetra*-*O*-acetyl-β-*S*-D-galactopyranoside **(*E*-14):** From Xantphos Pd-G_3 (22 mg, 0.023 mmol), **13** (186 mg, 0.51 mmol), azobenzene **12**[4,5] (150 mg, 0.46 mmol) and NEt_3 (71 µL, 0.51 mmol) in THF (2 mL) according to the General procedure III, compound **14** was obtained as a brown solid (234 mg, 90%). R_f = 0.5 (cyclohexane/EtOAc = 1/1), mp: 155.1°C; $[\alpha]_D^{22}$ = + 6.0 (*c* = 0.1, $CDCl_3$); ^{1}H NMR (300 MHz, $CDCl_3$): δ 7.85 (d, *J* = 8.7 Hz, 2H, H_{Ph}), 7.80 (d, *J* = 8.4 Hz, 2H, H_{Ph}), 7.62 (d, *J* = 8.5 Hz, 2H, H_{Ph}), 6.93 (d, *J* = 8.8 Hz, 2H, H_{Ph}), 5.44 (d, *J* = 3.3 Hz, 1H, H_4), 5.29 (t, *J* = 9.9 Hz, 1H, H_2), 5.09 (dd, *J* = 9.9, 3.3 Hz, 1H, H_3), 4.80 (d, *J* = 10.0 Hz, 1H, H_1), 4.18 (qd, *J* = 11.5, 6.7 Hz, 2H, $H_{6,6'}$), 3.99 (t, *J* = 6.4 Hz, 1H, H_5), 2.13 (s, 3H, CH_3), 2.11 (s, 3H, CH_3), 2.06 (s, 3H, CH_3), 1.98 (s, 3H, CH_3); ^{13}C NMR (75 MHz, $CDCl_3$): δ 170.7, 170.4, 170.3, 169.8, 159.1, 152.2, 147.1, 135.1 (Cq); 132.6, 125.3, 123.1, 116.0 (CH_{Ph}); 86.1 (C_1); 74.8 (C_5); 72.2 (C_3); 67.4 ($C_{2,4}$); 61.9 (C_6); 21.0, 20.8, 20.8, 20.7 (CH_3); HRMS (ESI) *m/z*: calcd for $C_{26}H_{28}N_2O_{10}S$ $[M+H]^+$ 561.1537, Found 561.1545.

(*E*)-4-(4′-Hydroxyphenylazo)phenyl-β-*S*-D-galactopyranoside **(*E*-15):** From **14** (156 mg, 0.28 mmol) and MeONa (280 µL, 0.14 mmol) according to the General procedure IV, compound **15** was obtained as a brown solid (105 mg). R_f = 0.4 (CH_2Cl_2/MeOH = 8:2), mp: 215°C; $[\alpha]_D^{22}$ = -100.0 (*c* = 0.1, MeOH); ^{1}H NMR (300 MHz, CD_3OD): δ 7.91-7.77 (m, 4H, H_{Ph}), 7.76-7.66 (m, 2H, H_{Ph}), 7.02-6.91 (m, 2H, H_{Ph}), 4.77 (d, *J* = 9.7 Hz, 1H, H_1), 3.99 (dd, *J* = 3.3, 1.1 Hz, 1H, H_4), 3.92-3.64 (m, 4H, $H_{2,5,6,6'}$), 3.60 (dd, *J* = 9.1, 3.3 Hz, 1H, H_3); ^{13}C NMR (75 MHz, CD_3OD): δ 162.2, 152.6, 147.5, 139.3 (Cq); 131.6, 125.9, 123.7, 116.8 (CH_{Ph}); 89.5 (C_1); 80.7 (C_5); 76.4 (C_3); 71.0 (C_2); 70.5 (C_5); 62.7 (C_6); HRMS (ESI) *m/z*: calcd for $C_{18}H_{20}N_2O_6S$ $[M+H]^+$ 393.1115, Found 393.1117.

(*E*)-4-[4′-(2-*tert*-Butyloxycarbonylaminoethyloxy)-phenylazo)phenyl-β-S-D-galactopyranoside **(*E*-16):** From **15** (79 mg, 0.20 mmol) and $BocNHCH_2CH_2Br$ (212 mg, 0.50 mmol), K_2CO_3 (56 mg, 0.4 mmol) according to the General procedure I, compound **16** (95 mg,

88%) was isolated as a yellow solid. R_f = 0.3 (CH_2Cl_2/MeOH = 10/1), mp: 159°C, $[\alpha]_D^{23}$: -65.6 (*c* = 0.5, MeOH); ^{1}H NMR (400 MHz, CD_3OD): δ 7.88 (d, *J* = 8.8 Hz, 2H, H_{Ph}), 7.80 (d, *J* = 8.4 Hz, 2H, H_{Ph}), 7.66 (d, *J* = 8.4 Hz, 2H, H_{Ph}), 7.08 (d, *J* = 9.2 Hz, 2H, H_{Ph}), 4.73 (d, *J* = 9.6 Hz, 1H, H_1), 4.10 (t, *J* = 5.6 Hz, 2H, OCH_2), 3.94 (d, *J* = 3.2 Hz, 1H, H_4), 3.79 (dd, *J* = 11.2, 6.8 Hz, 1H, H_6), 3.72 (dd, *J* = 11.2, 5.2 Hz, 1H, $H_{6'}$), 3.71-3.68 (m, 1H, H_5), 3.66 (d, *J* = 9.2 Hz, 1H, H_2), 3.57 (dd, *J* = 9.2, 3.6 Hz, 1H, H_3), 3.46 (t, *J* = 5.6 Hz, 2H, NCH_2), 1.44 (s, 9H, $3\times CH_3$); ^{13}C NMR (100 MHz, CD_3OD): δ 162.9, 158.5, 152.5, 148.3, 140.0 (Cq); 131.3, 125.7, 123.9, 115.9 (CH_{Ph}); 89.4 (C_1); 80.8 ($C_{2\text{ or }5}$); 80.3 (Cq); 76.3 (C_3); 70.9 ($C_{5\text{ or }2}$); 70.5 (C_4); 68.3 (OCH_2); 62.7 (C_6); 40.9 (NCH_2); 28.7 (CH_3); HRMS (ESI) *m/z*: Calcd for $C_{25}H_{34}N_3O_8S$ $[M+H]^+$: 536.2061, Found 536.2057.

(*E*)-4-[4′-(2-Aminoethyloxy)phenylazo)phenyl-β-*S*-D-galactopyranoside·HCl **(*E*-3):** From **16** (45 mg, 0.08 mmol) and AcCl (20 mg, 0.25 mmol) according to the General procedure II, compound **3** (35 mg, 90%) was isolated as a yellow solid. Mp: 224°C, $[\alpha]_D^{23}$: -91.0 (*c* = 0.1, H_2O); ^{1}H NMR (400 MHz, CD_3OD): δ 7.92 (d, *J* = 8.4 Hz, 2H, H_{Ph}), 7.81 (d, *J* = 8.4 Hz, 2H, H_{Ph}), 7.67 (d, *J* = 8.4 Hz, 2H, H_{Ph}), 7.17 (d, *J* = 9.2 Hz, 2H, H_{Ph}), 4.74 (d, *J* = 9.6 Hz, 1H, H_1), 4.33 (t, *J* = 4.8 Hz, 2H, OCH_2), 3.93 (d, *J* = 2.8 Hz, 1H, H_4), 3.80 (dd, *J* = 11.6, 6.8 Hz, 1H, H_6), 3.73 (dd, *J* =11.6, 5.2 Hz, 1H, $H_{6'}$), 3.70-3.64 (m, 2H, $H_{2,5}$), 3.54 (dd, *J* = 9.2, 3.2 Hz, 1H, H_3), 3.42 (t, *J* = 4.8 Hz, 2H, NCH_2); ^{13}C NMR (100 MHz, CD_3OD): δ 161.9, 152.4, 148.8, 140.3 (Cq); 131.2, 125.7, 124.0, 116.1 (CH_{Ph}); 89.3 (C_1); 80.8 ($C_{2\text{ or }5}$); 76.3 (C_3); 70.9 ($C_{5\text{ or }2}$); 70.5 (C_4); 65.6 (OCH_2); 62.7 (C_6); 40.3 (NCH_2); HRMS (ESI) *m/z*: Calcd for $C_{20}H_{26}N_3O_6S$ $[M+H]^+$: 436.1537, Found 436.1535.

(*E*)-3-(4′-Hydroxyphenylazo)phenyl-2,3,4,6-tetra-*O*-acetyl-β-*S*-D-galactopyranoside **(*E*-18):** From Xantphos Pd-G_3 (19 mg, 0.02 mmol), **13** (161 mg, 0.44 mmol), azobenzene **17**[4,5] (130 mg, 0.4 mmol) and NEt_3 (65 µL, 0.44 mmol) in THF (1.65 mL) according to the General procedure III, compound **18** was obtained as an orange solid (219 mg, 98%). R_f = 0.5 (cyclohexane/EtOAc = 1:1), mp 170°C; $[\alpha]_D^{22}$ = -33.0 (*c* = 0.1, $CDCl_3$); ^{1}H NMR (300 MHz, $CDCl_3$): δ 8.03 (t, *J* = 1.9 Hz, 1H, H_{Ph}), 7.93-7.82 (m, 2H, H_{Ph}), 7.82-7.77 (m, 1H, H_{Ph}), 7.55 (dt, *J* = 7.8, 1.5 Hz, 1H, H_{Ph}), 7.44 (t, *J* = 7.8 Hz, 1H, H_{Ph}), 6.99-6.85 (m, 2H, H_{Ph}), 5.44 (dd, *J* = 3.4, 1.1 Hz, 1H, H_4), 5.30 (t, *J* = 9.9 Hz, 1H, H_2), 5.09 (dd, *J* = 9.9, 3.3 Hz, 1H, H_3), 4.82 (d, *J* = 9.9 Hz, 1H, H_1), 4.21-4.11 (m, 2H, $H_{6,6'}$), 3.99 (ddd, *J* = 7.0, 5.8, 1.1 Hz, 1H, H_5), 2.11 (s, 3H, CH_3), 2.09 (s, 3H, CH_3), 1.99 (s, 3H, CH_3), 2.00(s, 3H, CH_3); ^{13}C NMR (75 MHz, $CDCl_3$): δ 170.4, 170.5, 170.3, 169.7, 159.0, 153.2, 147.1, 133.9, 133.8, 129.6, 125.9 (Cq); 125.3, 122.7, 116.0 (CH_{Ph}); 86.5 (C_1); 74.7 (C_5); 72.2 (C_3); 67.5 ($C_{2,4}$); 61.9 (C_6);, 21.0, 20.8, 20.7 (CH_3); HRMS (ESI) *m/z* calcd for $C_{26}H_{28}N_2O_{10}S$ $[M+H]^+$ 561.1537, found 561.1544.

(*E*)-3-(4′-Hydroxy-phenylazo)phenyl-β-*S*-D-galactopyranoside **(*E*-19):** From **18** (218 mg, 0.39 mmol) and MeONa (390 µL, 0.19 mmol) according to the General procedure IV, compound **19** was obtained as a red solid (150 mg). R_f = 0.3 (CH_2Cl_2/MeOH = 8:2); mp: 187.5°C; $[\alpha]_D^{22}$ = -22.0 (*c* = 0.05, MeOH); ^{1}H NMR (300 MHz, CD_3OD): δ 8.09-8.03 (m, 1H, H_{Ph}), 7.90-7.82 (m, 2H, H_{Ph}), 7.77-7.71 (m, 1H, H_{Ph}), 7.69-7.63 (m, 1H, H_{Ph}), 7.48 (t, *J* = 7.9 Hz, 1H, H_{Ph}), 7.00-6.90 (m, 2H, H_{Ph}), 4.72 (d, *J* = 9.6 Hz, 1H, H_1), 3.97 (dd, *J* = 3.3, 1.0 Hz, 1H, H_4), 3.89-3.62 (m, 4H, $H_{2,5,6,6'}$), 3.57 (dd, *J* = 9.2, 3.3 Hz, 1H, H_3); ^{13}C NMR (75 MHz, CD_3OD): δ 147.5, 137. 5, 133.5, 130.4 (Cq); 126.1, 124.9, 122.6, 116.8 (CH_{Ph}); 90.0 (C_1); 80.6 (C_5); 76.4 (C_3); 71.0 (C_2); 70.4 (C_4); 62.6 (C_6); HRMS (ESI) *m/z*: calcd for $C_{18}H_{20}N_2O_6S$ $[M+H]^+$ 393.1115, Found 393.1189.

(*E*)-3-[4′-(2-*tert*-Butyloxycarbonylaminoethyloxy)phenylazo)phenyl-β-*S*-D-galactopyranoside **(*E*-20):** From **19** (150 mg, 0.38 mmol) and $BocNHCH_2CH_2Br$ (213 mg, 0.95 mmol), K_2CO_3

(157 mg, 1.14 mmol) according to the General procedure I, compound **20** (101 mg, 50%) was isolated as an orange solid. R_f = 0.28 (CH_2Cl_2/MeOH = 10/1), mp: 114°C, $[\alpha]_D^{23}$: -36.1 (*c* = 0.5, MeOH); ^{1}H NMR (400 MHz, CD_3OD): δ 8.05 (t, *J* = 2.0 Hz, 1H, H_{Ph}), 7.91 (d, *J* = 9.2 Hz, 2H, H_{Ph}), 7.74 (ddd, *J* = 7.6, 1.6, 0.4 Hz, 1H, H_{Ph}), 7.65 (dt, *J* = 8.0, 0.8 Hz, 1H, H_{Ph}), 7.46 (t, *J* = 8.0 Hz, 1H, H_{Ph}), 7.09 (d, *J* = 8.4 Hz, 2H, H_{Ph}), 4.69 (d, *J* = 9.6 Hz, 1H, H_1), 4.11 (t, *J* = 5.2 Hz, 2H, OCH_2), 3.92 (d, *J* = 2.8 Hz, 1H, H_4), 3.80 (dd, *J* = 11.6, 7.2 Hz, 1H, H_6), 3.73 (dd, *J* = 11.6, 5.2 Hz, 1H, $H_{6'}$), 3.68-3.61 (m, 2H, $H_{2,5}$), 3.52 (dd, *J* = 8.8, 3.2 Hz, 1H, H_3), 3.47 (t, *J* = 5.6 Hz, 2H, NCH_2), 1.45 (s, 9H, 3×CH_3); ^{13}C NMR (100 MHz, CD_3OD): δ 163.1, 158.5, 154.2, 148.2, 137.6 (Cq); 133.5, 130.5, 125.9, 124.8, 122.7, 115.9 (CH_{Ph}); 90.0 (C_1); 80.6 (C_5); 80.3 (Cq); 76.3 (C_3); 71.0 (C_2); 70.4 (C_4); 68.3 (OCH_2); 62.5 (C_6); 40.9 (NCH_2); 28.7 (CH_3); HRMS (ESI) *m/z*: Calcd for $C_{25}H_{34}N_3O_8S$ $[M+H]^+$: 536.2061, Found 536.2055.

(*E*)-3-[4′-(2-Aminoethyloxy)phenylazo)phenyl-β-*S*-D-galactopyranoside·HCl **(*E*-4):** From **20** (47 mg, 0.087 mmol) and AcCl (20 mg, 0.26 mmol) according to the General procedure II, compound **1** (35 mg, 85%) was isolated as an orange solid. mp: 208°C, $[\alpha]_D^{23}$: -17.9 (*c* = 0.2, H_2O); ^{1}H NMR (400 MHz, CD_3OD): δ 8.06 (t, *J* = 1.6 Hz, 1H, H_{Ph}), 7.96 (d, *J* = 8.8 Hz, 2H, H_{Ph}), 7.75 (dt, *J* = 8.4, 1.2 Hz, 1H, H_{Ph}), 7.67 (dt, *J* = 7.6, 1.6 Hz, 1H, H_{Ph}), 7.47 (t, *J* = 8.4 Hz, 1H, H_{Ph}), 7.18 (d, *J* = 8.8 Hz, 2H, H_{Ph}), 4.69 (d, *J* = 10.0 Hz, 1H, H_1), 4.33 (t, *J* = 5.2 Hz, 2H, OCH_2), 3.92 (d, *J* = 3.2 Hz, 1H, H_4), 3.80 (dd, *J* = 11.6, 7.2 Hz, 1H, H_6), 3.73 (dd, *J* = 11.6, 5.2 Hz, 1H, $H_{6'}$), 3.68-3.61 (m, 2H, $H_{2,5}$), 3.52 (dd, *J* = 9.2, 3.2 Hz, 1H, H_3), 3.42 (t, *J* = 5.2 Hz, 2H, NCH_2); ^{13}C NMR (100 MHz, CD_3OD): δ 162.1, 154.2, 148.7, 137.7 (Cq); 133.7, 130.6, 125.9, 124.8, 122.8, 116.1 (CH_{Ph}); 89.9 (C_1); 80.6 (C_5); 76.3 (C_3); 71.0 (C_2); 70.4 (C_4); 65.6 (OCH_2); 62.6 (C_6); 40.2 (NCH_2); HRMS (ESI) *m/z*: Calcd for $C_{20}H_{26}N_3O_6S$ $[M+H]^+$: 436.1537, Found 436.1537.

(*E*)-2-(4′-Hydroxyphenylazo)phenyl-2,3,4,6-tetra-*O*-acetyl-β-*S*-D-galactopyranoside **(*E*-22):** From Xantphos Pd-G3 (24.5 mg, 0.0255 mmol), **13** (204 mg, 0.56 mmol), azobenzene **21**[4,5] (165 mg, 0.51 mmol) and NEt_3 (65 µL, 0.44 mmol) in THF (2.1 mL) according to the General procedure III, compound **22** was obtained as an orange solid (167 mg, 54%). R_f = 0.5 (cyclohexane/EtOAc = 1:1), mp: 165.6°C; $[\alpha]_D^{22}$ = - 42.0 (*c* = 0.1, $CDCl_3$); ^{1}H NMR (300 MHz, $CDCl_3$): δ 7.92-7.84 (m, 2H, H_{Ph}), 7.71-7.60 (m, 2H, H_{Ph}), 7.37-7.32 (m, 2H, H_{Ph}), 7.00-6.90 (m, 2H, H_{Ph}), 5.47 (dd, *J* = 3.4, 1.1Hz, 1H, H_4), 5.40 (t, *J* = 10.0Hz, 1H, H_2), 5.10 (dd, *J* = 9.9, 3.4 Hz, 1H, H_3), 4.99 (d, *J* = 10.1Hz, 1H, H_1), 4.18-4.10 (m, 2H, $H_{6,6'}$), 4.00 (ddd, *J* = 7.1, 5.9, 1.1 Hz, 1H, H_5), 2.17 (s, 3H, CH_3), 2.03 (s, 3H, CH_3), 1.99 (s, 3H, CH_3), 1.97 (s, 3H, CH_3); ^{13}C NMR (75 MHz, $CDCl_3$): δ 170.6, 170.5, 170.3, 169.8, 159.4, 150.9, 147.2, 130.6, 130.4, 127.8, 125.6, 117.6, 116.1(CH_{Ph}); 85.3 (C_1); 74.7 (C_5); 72.3 (C_3); 67.6 (C_2);, 67.5 (C_4); 61.9 (C_6); 21.2, 20.9, 20.8, 20.7 (CH_3); HRMS (ESI) *m/z*: calcd for $C_{26}H_{28}N_2O_{10}S$ $[M+H]^+$ 561.1537, Found 561.1543.

(*E*)-2-(4′-Hydroxyphenylazo)phenyl-β-*S*-D-galactopyranoside **(*E*-23):** From **22** (156 mg, 0.28 mmol) and MeONa (280 µL, 0.14 mmol) according to the General procedure IV, compound **23** was obtained as a brown solid (105 mg). R_f = 0.2 (CH_2Cl_2/MeOH = 4/1); mp: 175.3°C; $[\alpha]_D^{22}$ = +43.0 (*c* = 0.1, MeOH); ^{1}H NMR (300 MHz, CD_3OD): δ 7.95-7.90 (m, 2H, H_{Ph}), 7.81 (dd, *J* = 8.0, 1.3 Hz, 1H, H_{Ph}), 7.66 (dd, *J* = 8.0, 1.5 Hz, 1H, H_{Ph}), 7.48-7.40 (m, 1H, H_{Ph}), 7.37-7.27 (m, 1H, H_{Ph}), 7.04-6.93 (m, 2H, H_{Ph}), 4.92 (d, *J* = 9.7 Hz, 1H, H_1), 4.04 (dd, *J* = 3.3 Hz, 1H, H_4), 3.91-3.73 (m, 4H, $H_{2,5,6,6'}$), 3.66 (dd, *J* = 9.1, 3.3 Hz, 1H, H_3); ^{13}C NMR (75 MHz, CD_3OD): δ 177.8, 138.1, 131.8, 129.8, 127.1, 126.4, 117.4, 116.8 (CH_{Ph}); 87.5 (C_1); 80.5 (C_5); 76.4 (C_3); 71.1 (C_2); 70.6 (C_4); 62.7 (C_6); HRMS (ESI) *m/z*: calcd for $C_{18}H_{20}N_2O_6S$ $[M+Na]^+$ 415.0934, Found 415.0941.

(E)-2-[4′-(2-*tert*-Butyloxycarbonylaminoethyloxy)phenylazo)phenyl-β-*S*-D-galactopyranoside **(E-24):** From **23** (30 mg, 0.076 mmol) and $BocNHCH_2CH_2Br$ (26 mg, 0.12 mmol), K_2CO_3 (21 mg, 0.15 mmol) according to the General procedure I, compound **24** (35 mg, 88%) was isolated as an orange solid after purification by CombiFlash Rf+ (CH_2Cl_2/MeOH = 20/1). R_f = 0.3 (CH_2Cl_2/MeOH = 10/1), mp: 124°C, $[\alpha]_D^{23}$: +95.4 (c = 0.1, MeOH); ^{1}H NMR (400 MHz, CD_3OD): δ 7.91 (d, J = 8.8 Hz, 2H, H_{Ph}), 7.74 (d, J = 6.8 Hz, 1H, H_{Ph}), 7.61 (dd, J = 8.0, 1.6 Hz, 1H, H_{Ph}), 7.39 (td, J = 7.6, 2.0 Hz, 1H, H_{Ph}), 7.24 (td, J = 8.0, 0.8 Hz, 1H, H_{Ph}), 7.08 (d, J = 8.8 Hz, 2H, H_{Ph}), 4.86 (d, J = 9.6 Hz, 1H, H_1), 4.10 (t, J = 5.6 Hz, 2H, OCH_2), 3.94 (d, J = 3.2 Hz, 1H, H_4), 3.80-3.68 (m, 4H, $H_{2,5,6,6'}$), 3.57 (dd, J = 9.2, 3.6 Hz, 1H, H_3), 3.46 (t, J = 5.6 Hz, 2H, NCH_2), 1.44 (s, 9H, 3×CH_3); ^{13}C NMR (100 MHz, CD_3OD): δ 163.1, 158.5, 150.7, 148.6, 139.0 (Cq); 132.2, 129.4, 126.9, 126.4, 117.3, 115.9 (CH_{Ph}); 87.3 (C_1); 80.7 (C_5); 80.3 (Cq); 76.4 (C_3); 71.1 (C_2); 70.5 (C_4); 68.3 (OCH_2); 62.7 (C_6); 40.9 (NCH_2); 28.7 (CH_3); HRMS (ESI) *m/z*: Calcd for $C_{25}H_{34}N_3O_8S$ $[M+H]^+$: 536.2061, Found 536.2057.

(E)-2-[4′-(2-Aminoethyloxy)phenylazo)phenyl-β-*S*-D-galactopyranoside·HCl **(E-5):** From **24** (55 mg, 0.10 mmol) and AcCl (24 mg, 0.30 mmol) according to the General procedure II, compound **5** (36 mg, 77%) was isolated as a green solid. mp: 204°C, $[\alpha]_D^{23}$: +14.3 (c = 0.3, H_2O); ^{1}H NMR (400 MHz, CD_3OD): δ 7.96 (d, J = 8.8 Hz, 2H, H_{Ph}), 7.76 (dd, J = 8.4, 1.2 Hz, 1H, H_{Ph}), 7.63 (dd, J = 7.6, 1.2 Hz, 1H, H_{Ph}), 7.42 (td, J = 7.6, 1.6 Hz, 1H, H_{Ph}), 7.26 (td, J = 8.0, 1.2 Hz, 1H, H_{Ph}), 7.17 (d, J = 9.2 Hz, 2H, H_{Ph}), 4.89-4.88 (m, 1H, H_1), 4.34 (t, J = 4.8 Hz, 2H, OCH_2), 3.96 (d, J = 3.2 Hz, 1H, H_4), 3.81-3.71 (m, 4H, $H_{2,5,6,6'}$), 3.58 (dd, J = 9.2, 3.2 Hz, 1H, H_3), 3.42 (t, J = 4.8 Hz, 2H, NCH_2); ^{13}C NMR (100 MHz, CD_3OD): δ 162.1, 150.6, 149.1, 139.2 (Cq); 132.4, 129.4, 126.9, 126.2, 117.3, 116.1 (CH_{Ph}); 87.2 (C_1); 80.7 (C_5); 76.4 (C_3); 71.1 (C_2); 70.5 (C_4); 65.7 (OCH_2); 62.7 (C_6); 40.2 (NCH_2); HRMS (ESI) *m/z*: Calcd for $C_{20}H_{26}N_3O_6S$ $[M+H]^+$: 436.1537, Found 436.1535.

References:

1. Kuhaudomlarp, S.; Gillon, E.; Varrot, A.; Imberty, A. *Methods Mol. Biol.* **2020**, *2132*, 257-266.
2. Bruneau, A.; Roche, M.; Hamze, A.; Brion, J.; Alami, M.; Messaoudi, S. *Chem. Eur. J.* **2015**, *21*, 8375-8379.
3. Rodrigue, J.; Ganne, G.; Blanchard, B.; Saucier, C.; Giguère, D.; Shiao, T. C.; Varrot, A.; Imberty, A.; Roy, R. *Org. Biomol. Chem.* **2013**, *11*, 6906-6918.
4. Schultzke, S.; Walther, M.; Staubitz, A. *Molecules* **2021**, *26*, 3916.
5. Ngaini, Z.; Hissam, M. A.; Mortadza, N. A.; Abd Halim, A. N.; Daud, A. I. *Nat. Prod. Res.* **2023**, 1486310 (1-11).

Detailed photochemical and photophysical procedures

Determination of the molar absorption coefficients (ε)

A mother solution of compound (*E*)-**1** was freshly prepared in H_2O in a precise concentration (c = 0.219 mmol·L^{-1}; 0.301 mg dissolved in 3000 µL). From the mother solution, a series of 7 to 10 daughter solutions were prepared with a concentration range from 2 µM to 37 µM, and their absorbance was measured independently by UV–vis absorption at room temperature (20 to 23 °C).

To ensure the reproducibility and evaluate the accuracy of the measurements, each recorded spectrum was divided by its concentration in order to obtain the corresponding normalized spectrum; only the overlapped normalized spectra are used for the determination of the molar absorption coefficient. Then, the absorption was taken at the maximum of wavelength (353 nm) to calculate the molar absorption coefficients by a linear regression fitting using the least squares method and with a forcing to pass through the origin of coordinates.

The molar absorption coefficient is calculated according to the BEER-LAMBERT law,

$$A(\lambda) = \varepsilon(\lambda) \cdot l \cdot C$$

with

$A(\lambda)$, represents the absorbance at the wavelength λ
$\varepsilon(\lambda)$, represents the molar absorption coefficient at the wavelength λ, in mol·L^{-1}·cm^{-1}
l, the distance of the trajectory in the media, in cm
C, the concentration of the substrate in mol·L^{-1}

Epsilon of the compound E-1

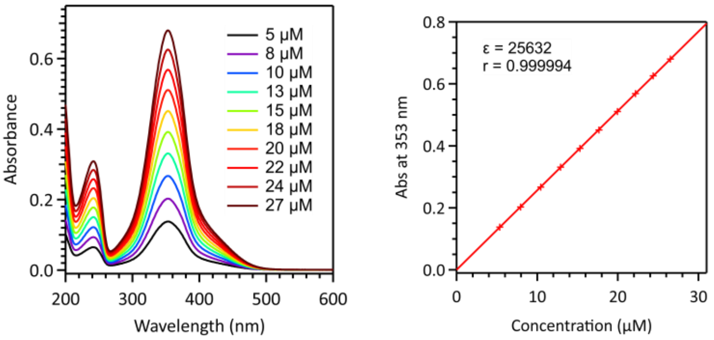

Figure S1. UV–vis absorption spectra of *E*-**1** measured in H_2O for the daughter solutions (left) and evolution of the absorbance (right) as function of the concentration at the maximum of wavelength (353 nm). Mother solution: 0.301 mg dissolved in 3000 µL of H_2O (c = 0.219 mmol·L^{-1}).

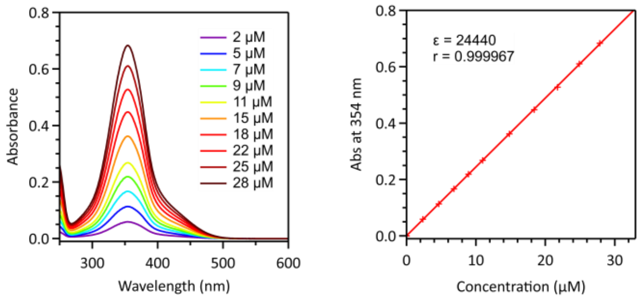

Figure S2. UV–vis absorption spectra of *E*-**1** measured in Tris buffer: DMSO (95/5) for the daughter solutions (left) and evolution of the absorbance (right) as function of the concentration at the maximum of wavelength (354 nm). Mother solution: 0.165 mg dissolved in a mixture solution of 150 µL DMSO and 2850 µL buffer (c = 0.121 mmol·L^{-1}).

Epsilon of the compound E-2

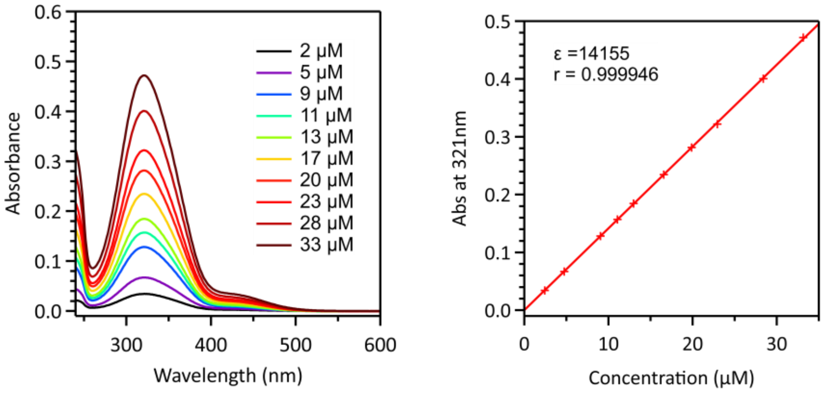

Figure S3. UV–vis absorption spectra of *E*-**2** measured in Tris buffer: DMSO (95/5) for the daughter solutions (left) and evolution of the absorbance (right) as function of the concentration at the maximum of wavelength (321 nm). Mother solution: 0.136 mg dissolved in a mixture solution of 150 µL DMSO and 2850 µL buffer (c = 0.099 mmol·L^{-1}).

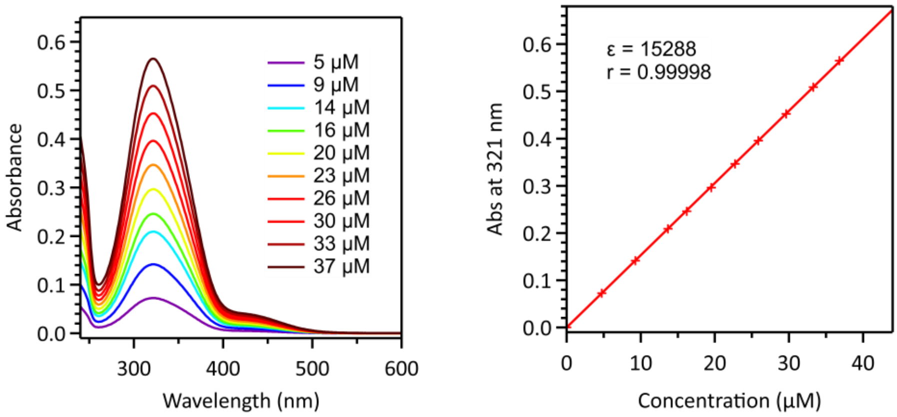

Figure S4. UV–vis absorption spectra of *E*-**2** measured in Tris buffer: DMSO (9/1) for the daughter solutions (left) and evolution of the absorbance (right) as function of the concentration at the maximum of wavelength (321 nm). Mother solution: 0.220 mg dissolved in a mixture solution of 200 µL DMSO and 1800 µL buffer (c = 0.241 mmol·L^{-1}).

Epsilon of the compound E-3

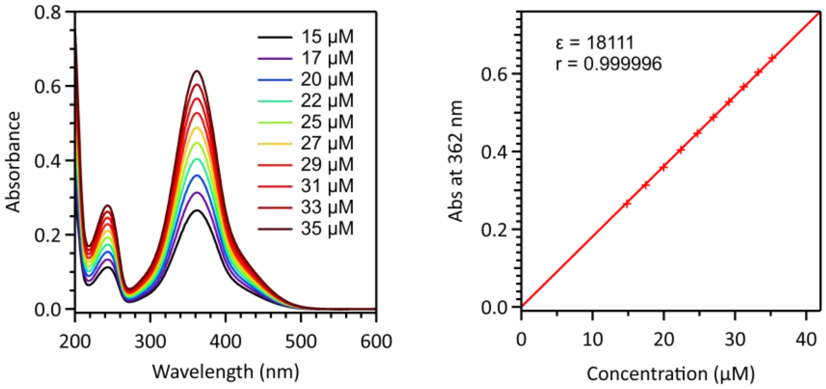

Figure S5. UV–vis absorption spectra of *E*-**3** measured in H_2O for the daughter solutions (left) and evolution of the absorbance (right) as function of the concentration at the maximum of wavelength (362 nm). Mother solution: 0.210 mg dissolved in 3000 µL of H_2O (c = 0.148 mmol·L^{-1}).

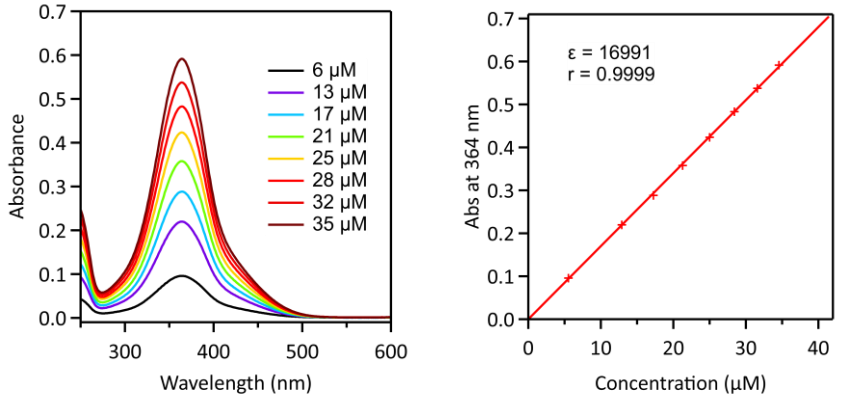

Figure S6. UV–vis absorption spectra of *E*-**3** measured in Tris buffer: DMSO (9/1) for the daughter solutions (left) and evolution of the absorbance (right) as function of the concentration at the maximum of wavelength (364 nm). Mother solution: 0.164 mg dissolved in a mixture solution of 300 μL DMSO and 2700 μL buffer (c = 0.116 mmol·L^{-1}).

Epsilon of the compound E-4

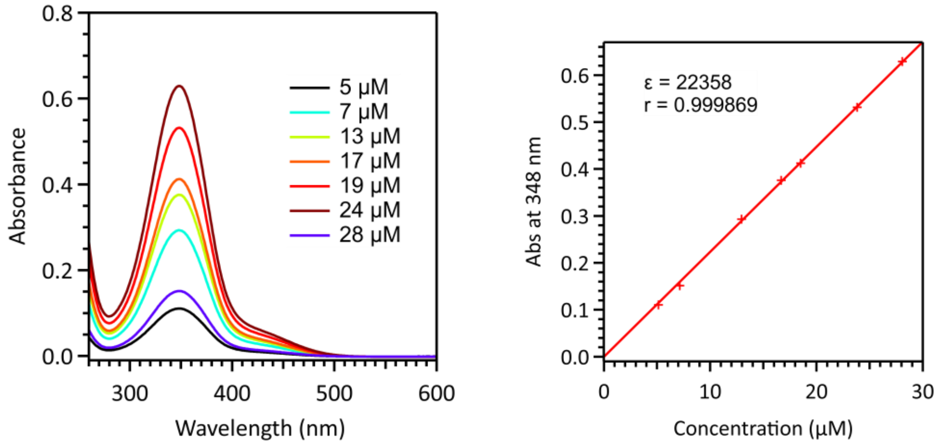

Figure S7. UV–vis absorption spectra of *E*-**4** measured in Tris buffer: DMSO (9:1) for the daughter solutions (left) and evolution of the absorbance (right) as function of the concentration at the maximum of wavelength (348 nm). Mother solution: 0.371 mg dissolved in a mixture solution of 300 μL DMSO and 2700 μL buffer (c = 0.262 mmol·L^{-1}).

Epsilon of the compound E-5

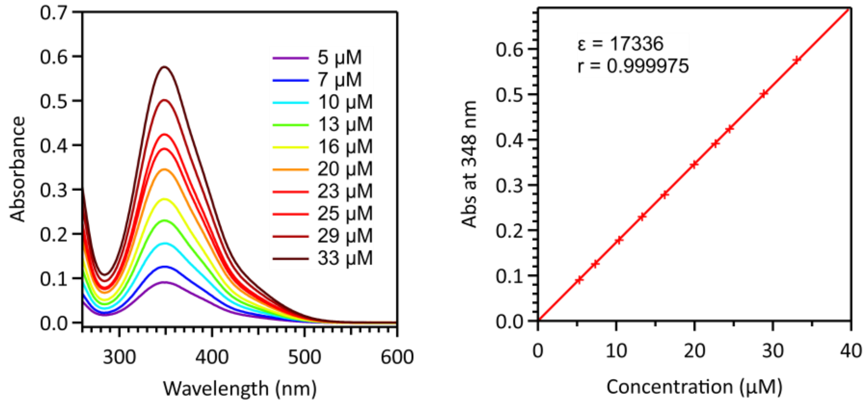

Figure S8. UV–vis absorption spectra of *E*-**5** measured in Tris buffer: DMSO (9/1) for the daughter solutions (left) and evolution of the absorbance (right) as function of the concentration at the maximum of wavelength (348 nm). Mother solution: 0.254 mg dissolved in a mixture solution of 200 μL DMSO and 1800 μL (c = 0.269 mmol·L^{-1}).

Determination of the half-lifes

General procedure, illustrated with the case of compound Z-1 half-life in D_2O

The thermal $Z \rightarrow E$ isomerization was tracked by measuring UV–vis absorption spectra at about 22±1°C (room temperature). The obtained time-dependent absorption profiles, taking *Z*-**1** as an example (black cross, **Figure S9**), were fitted with the following first order rate equation:

$$Abs(t) = A_o - (A_o - A_i)e^{-kt}$$

with
A_o, the absorption of original *E*-isomer (holded parameter)
A_i, the absorption at the PSS_{370} (holded parameter)
k, the rate constant of thermal isomerization
t, the time in minutes

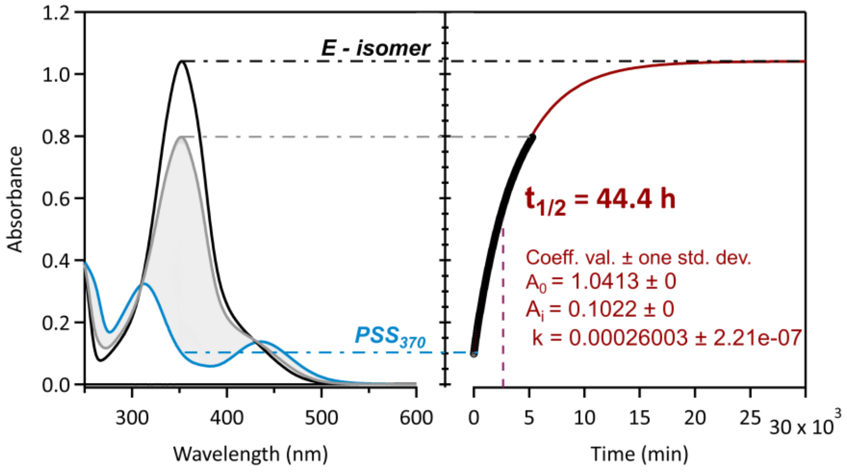

Figure S9: UV–vis absorption spectra (left) and thermal return monitoring (right) of *Z*-**1** at 352 nm in D_2O at room temperature (≈ 22 °C). The reported data points are the result of a single kinetic measurement realized on a solution from the PSS_{370}: 0 to 5300 min (step = 20 min).

After fitting (dark red curve), the rate constant at 22 °C (*T* = 292 K) was obtained from the fitting parameters:

$$k_{292} = 2.6 \text{ x } 10^{-4} \text{ min}^{-1}$$

The half-life of *Z*-**1** at room temperature was then calculated:

$$t_{1/2} = \frac{\ln(2)}{k_T}$$

$$t_{1/2} = 2665.6 \text{ min} \cong 44.4 \text{ h}$$

Similarly, the half-life of *Z*-**2, 3, 4** and **5** was estimated by this method.

Half-life of compound Z-2 in Tris buffer/DMSO (95/5)

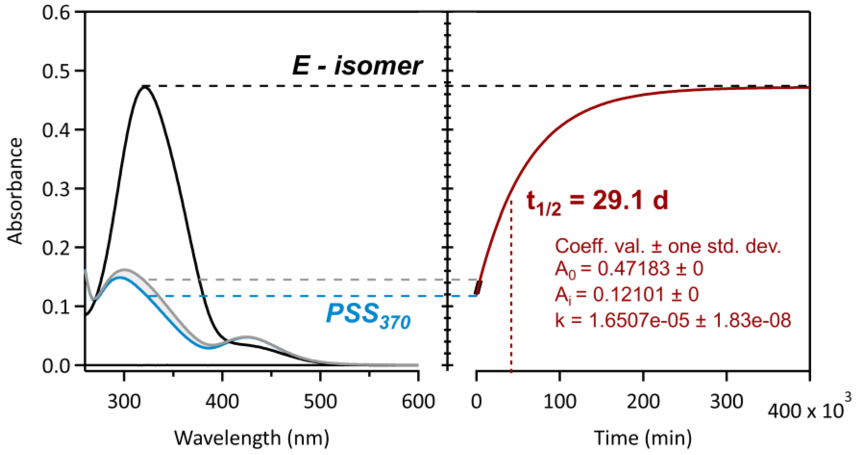

Figure S10: UV–vis absorption spectra (left) and thermal return monitoring (right) of *Z*-**2** at 321 nm in Tris buffer/DMSO (95/5) at room temperature (≈ 22 °C). The reported data points are the result of a single kinetic measurement realized on a solution from the PSS_{370}: 0 to 3930 min (step = 15 min).

$$k_{292} = 1.7 \times 10^{-5}\ min^{-1}$$
$$t_{1/2} = 41991.1\ min \cong 29.1\ days$$

Half-life of compound Z-3 in H_2O

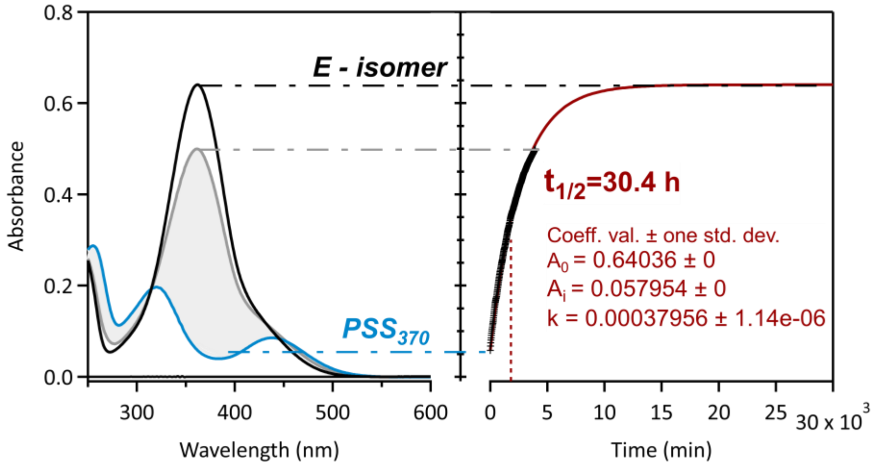

Figure S11: UV–vis absorption spectra (left) and thermal return monitoring (right) of *Z*-**3** at 362 nm in H_2O at room temperature (≈ 22 °C). The reported data points are the result of a single kinetic measurement realized on a solution from the PSS_{370}: 0 to 3940 min (step = 20 min).

$$k_{292} = 3.8 \times 10^{-4}\ min^{-1}$$
$$t_{1/2} = 1826.2\ min \cong 30.4\ h$$

***Half-life of compound Z-3* in Tris buffer/DMSO (9/1)**

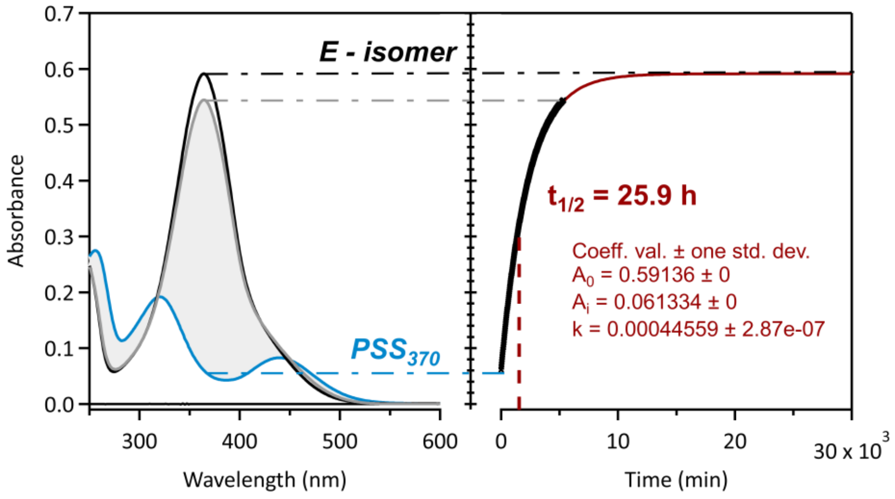

Figure S12: UV–vis absorption spectra (left) and thermal return monitoring (right) of *Z*-**3** at 364 nm in Tris buffer/DMSO (9/1) at room temperature (≈ 22 °C). The reported data points are the result of a single kinetic measurement realized on a solution from the PSS_{370}: 0 to 5340 min (step = 20 min).

$$k_{292} = 4.5 \text{ x } 10^{-4} \text{ min}^{-1}$$
$$t_{1/2} = 1555.6 \text{ min} \cong 25.9 \text{ h}$$

***Half-life of compound Z-4* in Tris buffer/DMSO (9/1)**

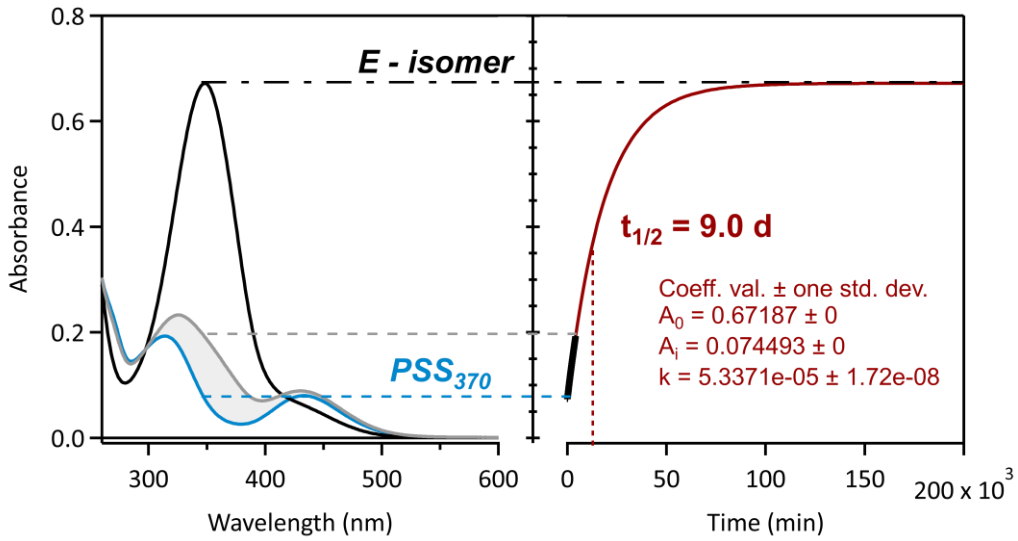

Figure S13: UV–vis absorption spectra (left) and thermal return monitoring (right) of *Z*-**4** at 348 nm in Tris buffer/DMSO (9/1) at room temperature (≈ 22 °C). The reported data points are the result of a single kinetic measurement realized on a solution from the PSS_{370}: 0 to 4095 min (step = 15 min).

$$k_{292} = 5.3 \text{ x } 10^{-5} \text{ min}^{-1}$$
$$t_{1/2} = 12987.6 \text{ min} \cong 9.0 \text{ days}$$

***Half-life of compound Z-5* in Tris buffer/DMSO (9/1)**

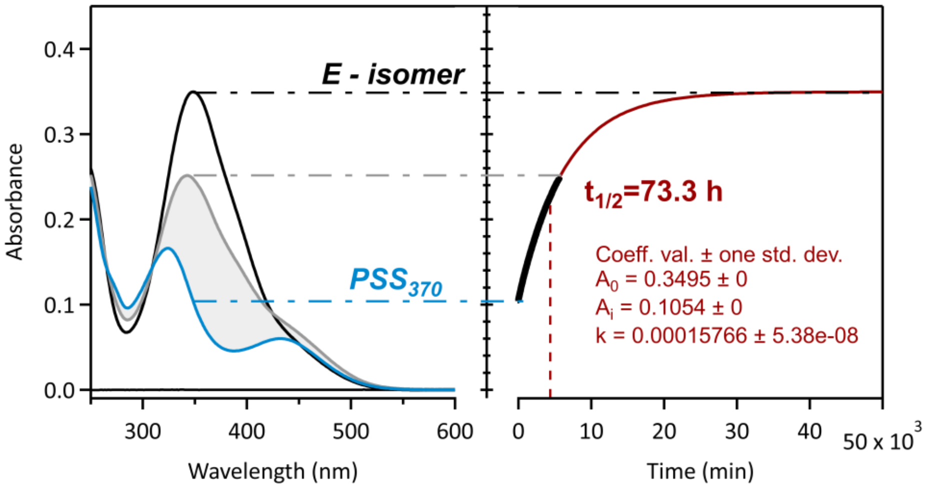

Figure S14: UV–vis absorption spectra (left) and thermal return monitoring (right) of *Z*-**5** at 348 nm in Tris buffer/DMSO (9/1) at room temperature (≈ 22 °C). The reported data points are the result of a single kinetic measurement realized on a solution from the PSS_{370}: 0 to 5595 min (step = 15 min).

$$k_{292} = 1.6 \text{ x } 10^{-4} \text{ min}^{-1}$$
$$t_{1/2} = 4396.5 \text{ min} \cong 73.3 \text{ h}$$

General procedure for the fatigue resistance measurements

Starting from a solution of compound containing 100% of the *E*-isomer, the solution was irradiated sequentially at 370 nm and at 485 nm or 438 nm. The absorbance was measured before and after each illumination realized with constant optical length distance. The sequence, which constitutes a cycle, was repeated for a minimum of 10 times.

Fatigue resistance of the compound 2

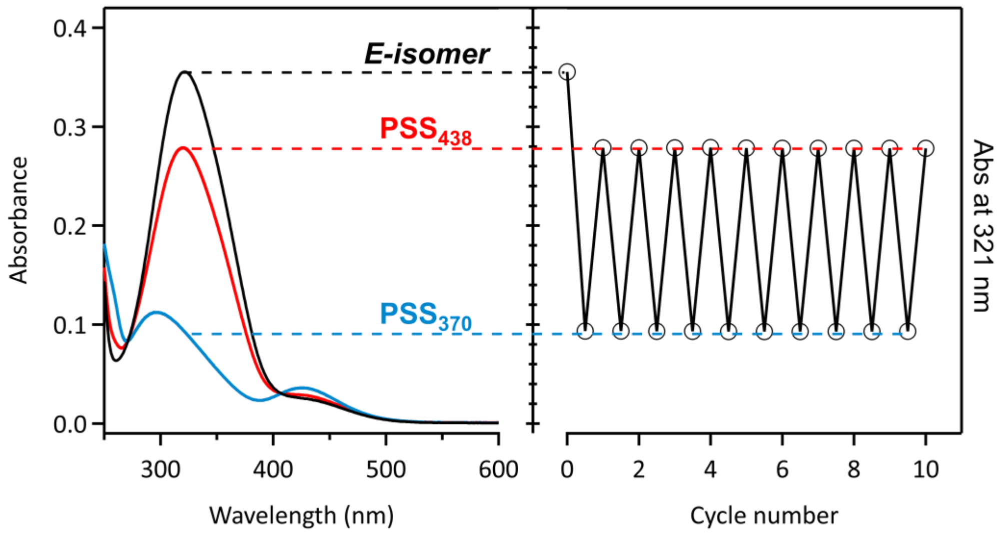

Figure S15: Left: absorption spectra of *E*-**2** (black line), PSS_{370} (blue line), PSS_{438} (red line) in Tris buffer/ DMSO (9/1); Right: Fatigue resistance followed by the absorption band at 321 nm under alternate 370 nm/438 nm irradiation cycles in Tris buffer/DMSO (9/1). Irradiation conditions: 40 s at P_{370} = 14.1 mW cm^{-2} and 180 s at P_{438} = 10.0 mW cm^{-2}.

Fatigue resistance of the compound 3

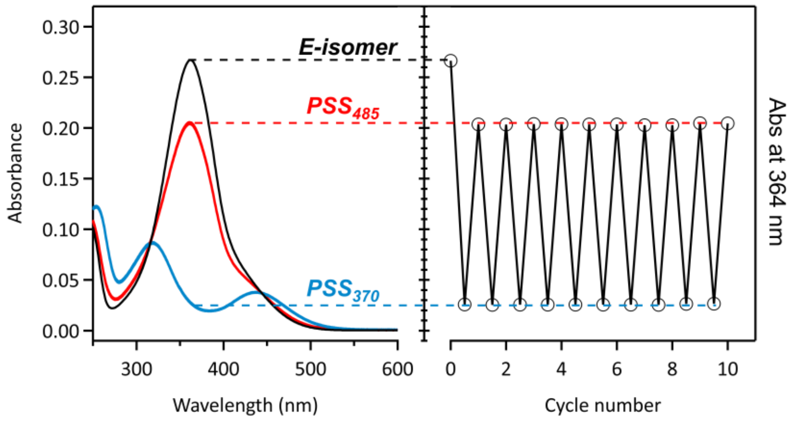

Figure S16: Left: absorption spectra of *E*-**3** (black line), PSS_{370} (blue line), PSS_{485} (red line) in Tris buffer/DMSO (9/1); Right: Fatigue resistance followed by the absorption band at 364 nm under alternate 370 nm/485 nm irradiation cycles in Tris buffer/DMSO (9/1). Irradiation conditions: 30 s at P_{370} = 12.8 mW cm^{-2} and 480 s at P_{485} = 1.5 mW cm^{-2}.

Fatigue resistance of the compound 4

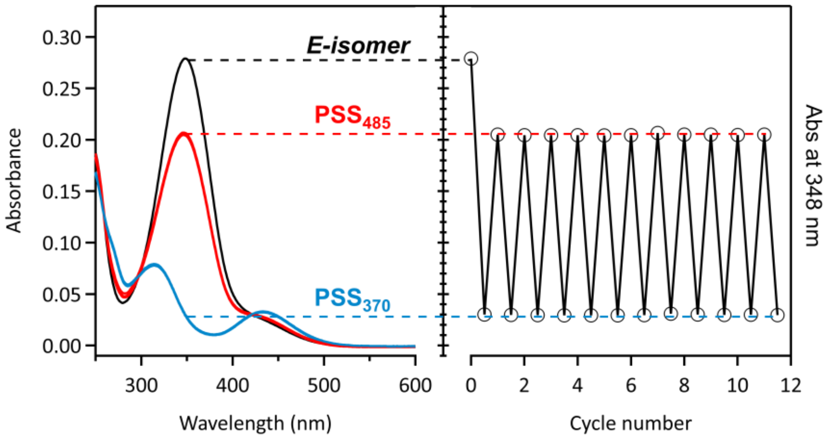

Figure S17: Left: absorption spectra of *E*-**4** (black line), PSS_{370} (blue line), PSS_{485} (red line) in Tris buffer: DMSO (9:1); Right: Fatigue resistance followed by the absorption band at 348 nm under alternate 370 nm/485 nm irradiation cycles in Tris buffer/DMSO (9/1). Irradiation conditions: 40 s at P_{370} = 14.1 mW cm^{-2} and 720 s at P_{485} = 1.6 mW cm^{-2}.

Fatigue resistance of the compound 5

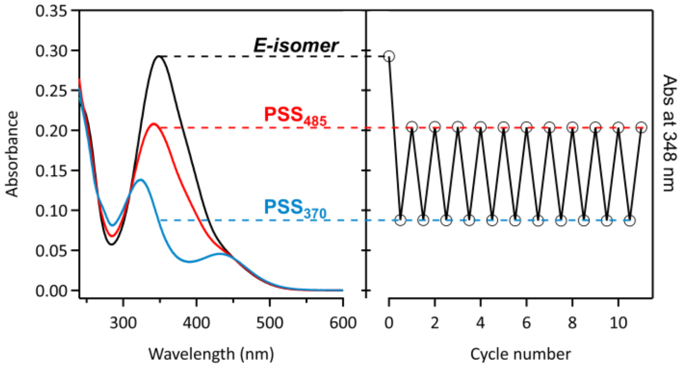

Figure S18: Left: absorption spectra of *E*-**5** (black line), PSS_{365} (blue line), PSS_{485} (red line) in Tris buffer/DMSO (9/1); Right: Fatigue resistance followed by the absorption band at 348 nm under alternate 370 nm/485 nm irradiation cycles in Tris buffer/DMSO (9/1). Irradiation conditions: 30 s at P_{370} = 14.1 mW cm^{-2} and 540 s at P_{485} = 1.6 mW cm^{-2}.

General procedure for the determination of the photoconvertion yields by absorption and ^{1}H NMR

A solution of *E*-isomer was extemporaneously prepared in D_2O or a mixture solvent D_2O/DMSO-d_6 9/1 with the maximum absorbance was close to 1. The deuterated solution was monitored by absorption and ^{1}H NMR before and after irradiation. It is worth noting that the absorbance was recorded first and then a small amount of the solution was taken for ^{1}H NMR monitoring. The sequence was repeated after irradiating the solution to the photostationary state (PSS), and ^{1}H NMR spectra were recorded with a relaxation delay of 0.5 s and 512 scans, in order to have reasonable acquisition time to prevent the impact of thermal return. Then, the corresponding photoconversion yield (CY) was calculated after integrating the pic area according to the following equation,

$$CY_i = \frac{A_E}{A_E + A_Z}$$

With,
CY_i, represents the conversion yield for a given state (i) of the solution,
A_E, represents the area taken under the pic of *E*-isomer signal,
A_Z, represents the area taken under the pic of *Z*-isomer signal.

Study of photoisomerization for compound 1

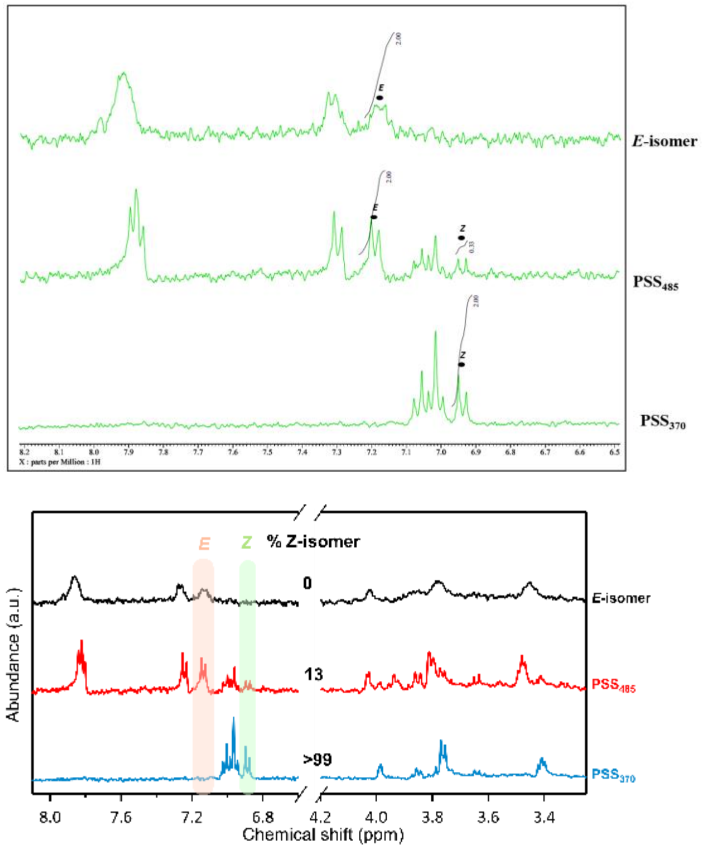

Figure S19: ^{1}H NMR spectra of *E*-**1**, PSS_{370} and PSS_{485} azobenzene region in D_2O. The integral values taken for the determination of the photoconversion yields.

Study of photoisomerization for compound 2

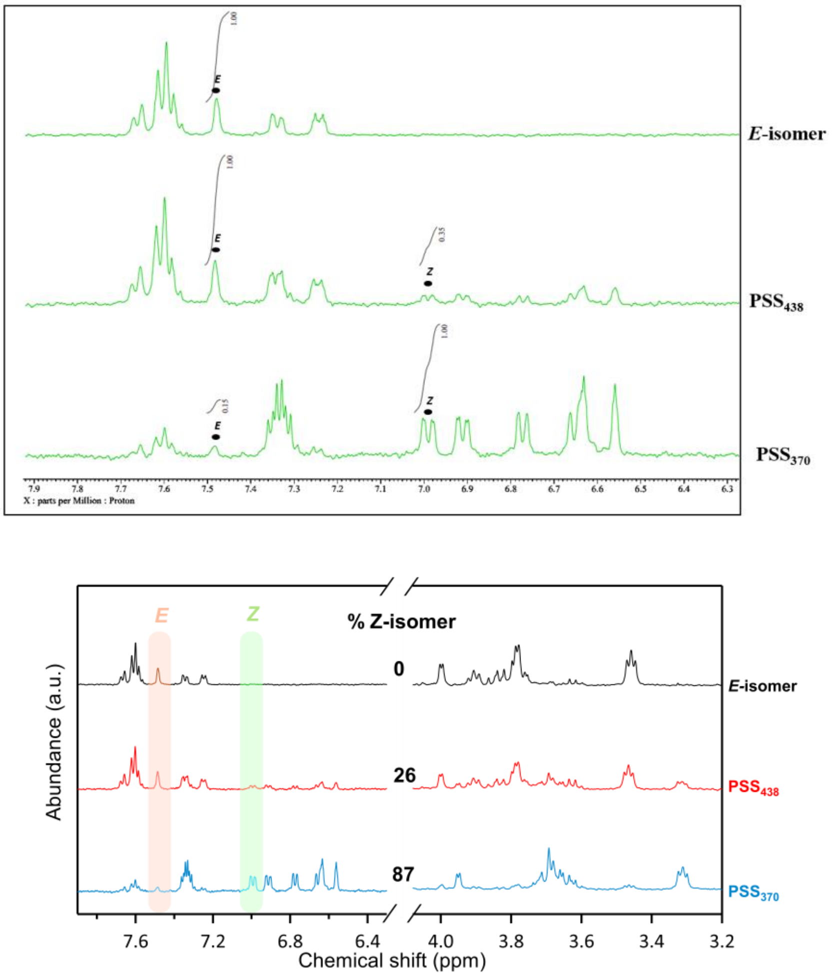

Figure S20: Partial ^{1}H NMR spectra of *E*-**2** (black line), PSS$_{370}$ (blue line) and PSS$_{438}$ (red line) in D_2O/DMSO-d_6 (9/1). Top graph: the region of azobenzene and the integral values taken for the determination of the photoconversion yields.

Study of photoisomerization for compound 3

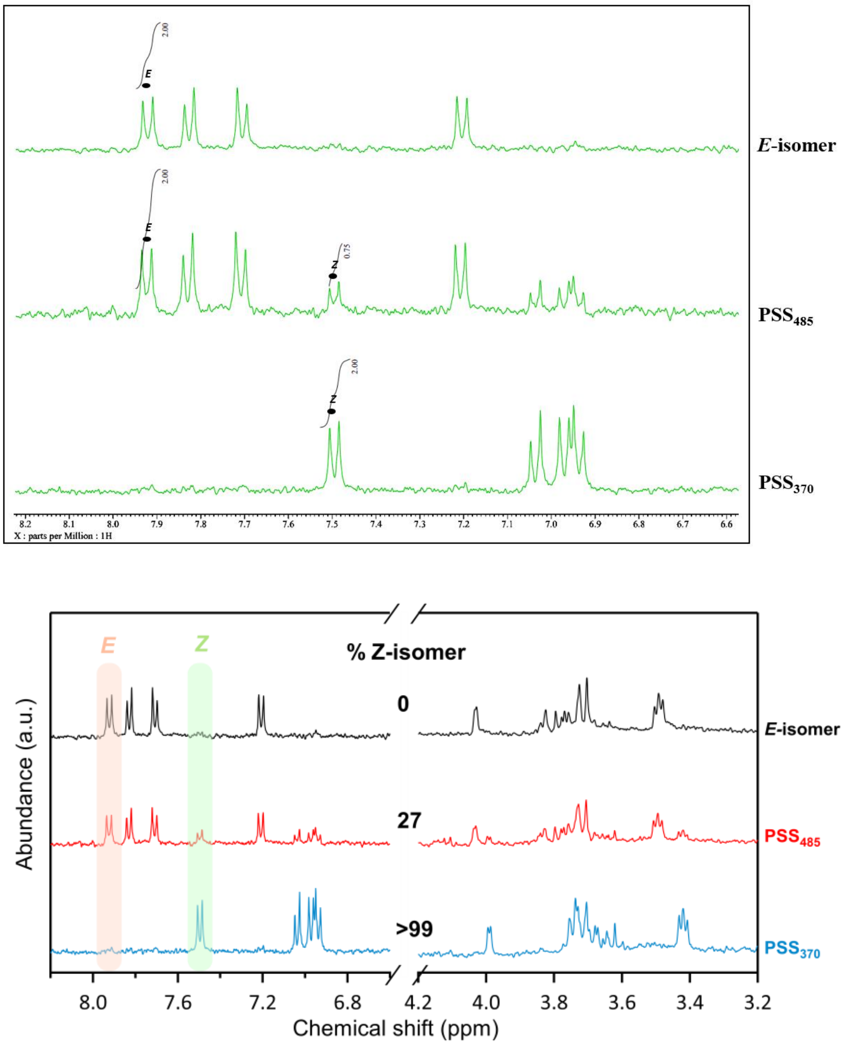

Figure S21: Partial ^{1}H NMR spectra of *E*-**3** (black line), PSS_{370} (blue line) and PSS_{485} (red line) in D_2O. Top graph: the region of azobenzene and the integral values taken for the determination of the photoconversion yields.

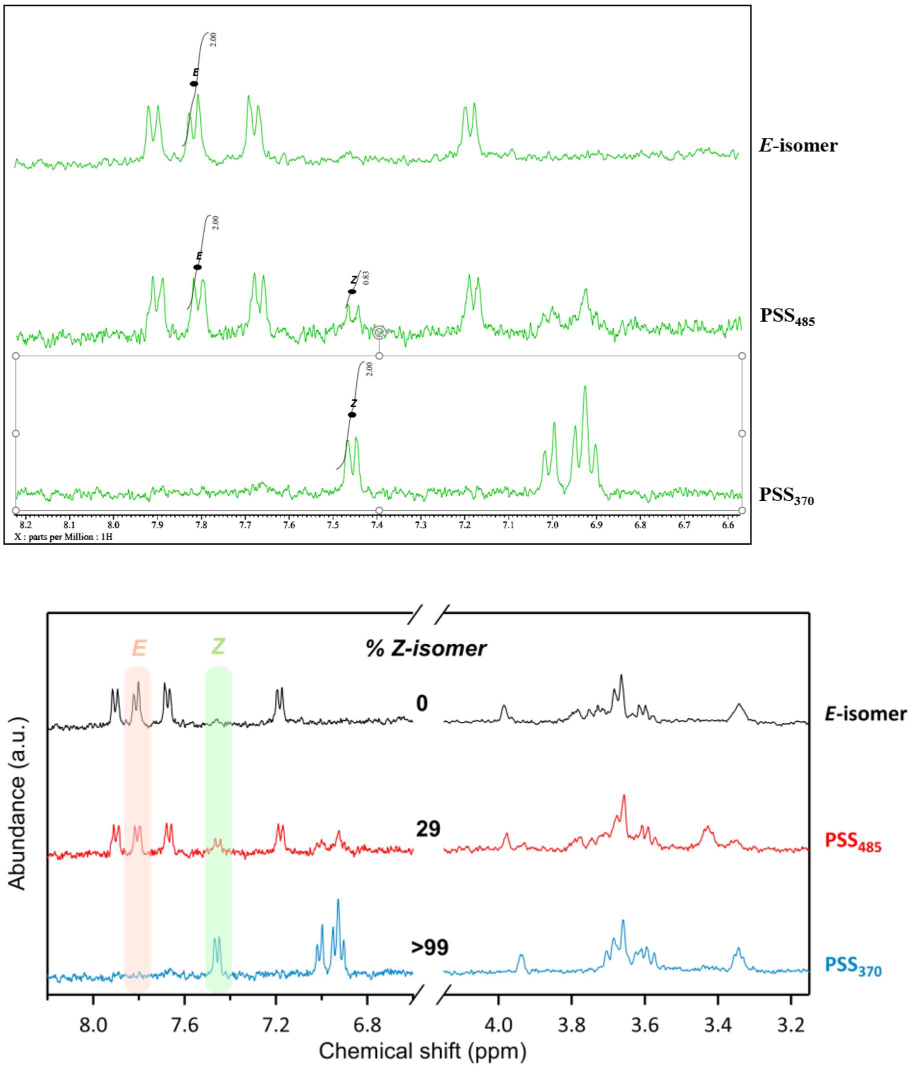

Figure S22: Partial ^{1}H NMR spectra of *E*-**3** (black line), PSS_{370} (blue line) and PSS_{485} (red line) in D_2O/DMSO-d_6 (9/1). Top graph: the region of azobenzene and the integral values taken for the determination of the photoconversion yields.

Study of photoisomerization for compound 4

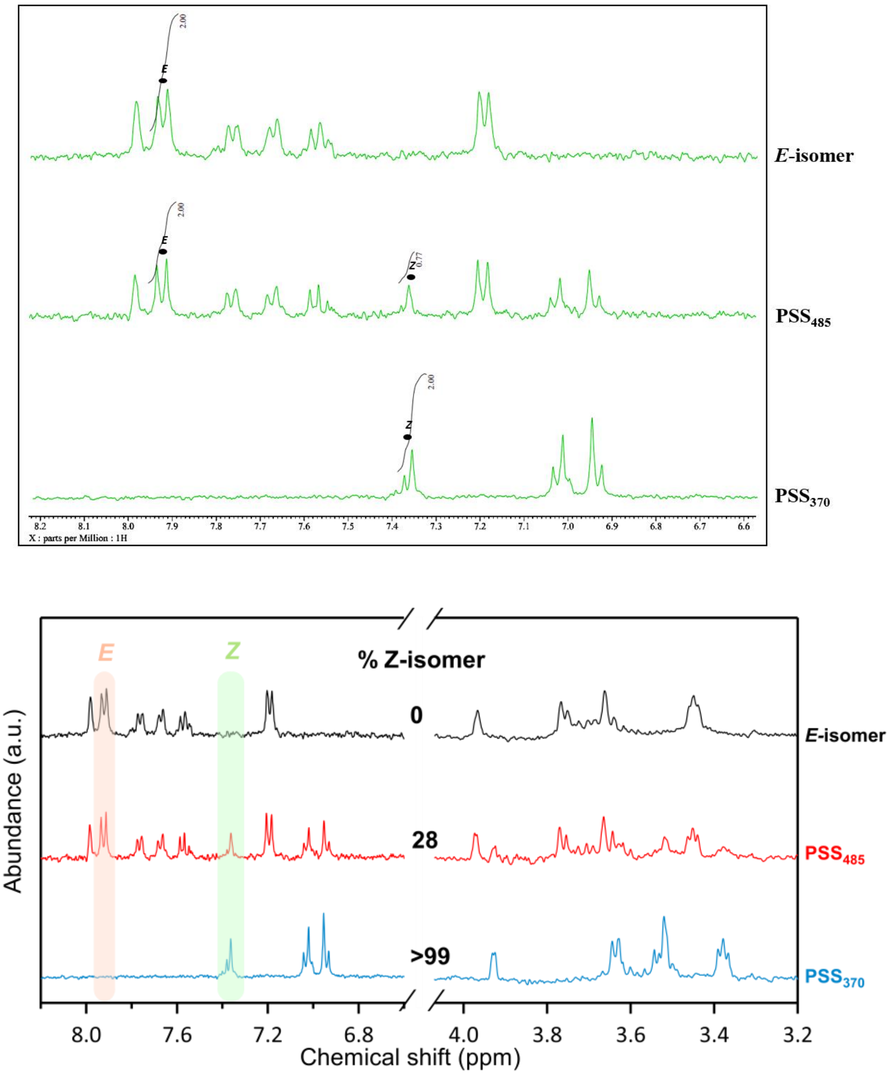

Figure S23: Partial ^{1}H NMR spectra of *E*-**4** (black line), PSS_{370} (blue line) and PSS_{485} (red line) in D_2O/DMSO-d_6 (9/1). Top graph: the region of azobenzene and the integral values taken for the determination of the photoconversion yields.

Study of photoisomerization for compound 5

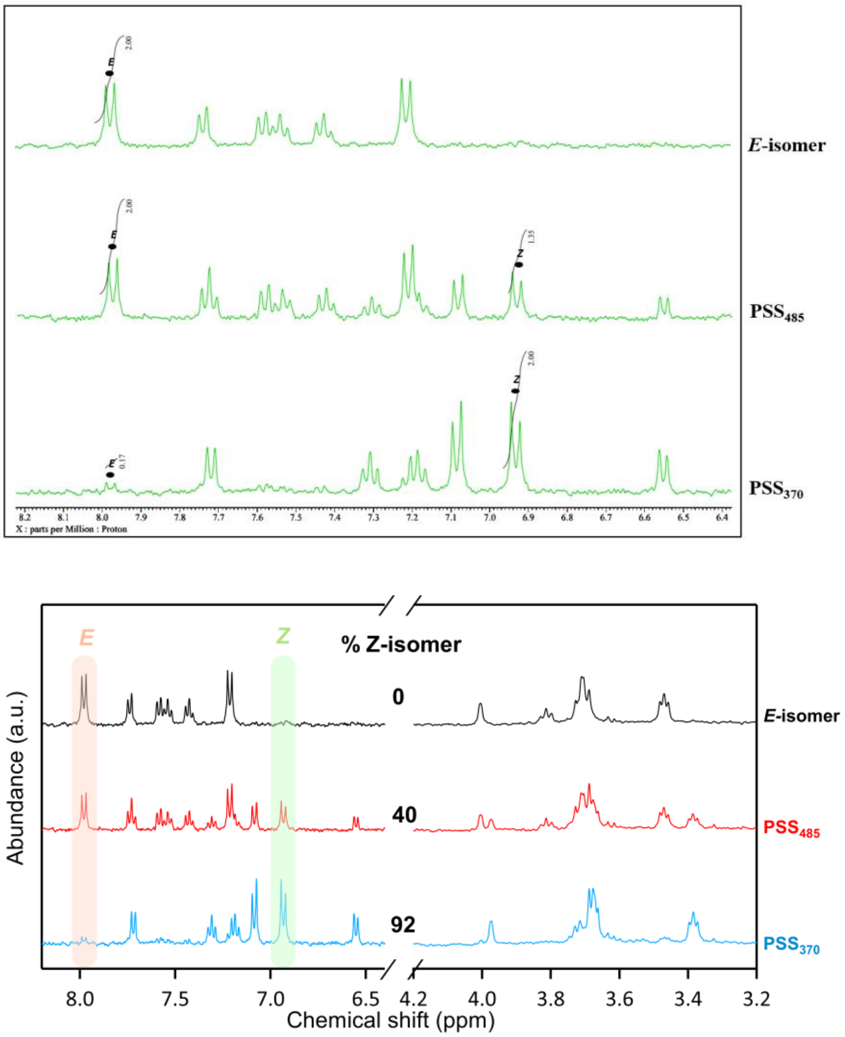

Figure S24: Partial ^{1}H NMR spectra of *E*-**5** (black line), PSS_{370} (blue line) and PSS_{485} (red line) in D_2O/DMSO-d_6 (9/1). Top graph: the region of azobenzene and the integral values taken for the determination of the photoconversion yields.

Concentration of ligands determined by spectroscopy

In previous studies on spectroscopic properties, we measured the molar absorption coefficient (Ɛ) of pure *E*-isomer, and the maximum photoconversion yield of the ligands from *E*-isomer to *Z*-isomer when they reached the photostationary state (PSS) after irradiation at 365 nm. The concentration of the ligands was calculated by following formula:

$$c = \frac{A(\lambda)}{\varepsilon(\lambda) l}$$

with

$A(\lambda)$, represents the absorbance at the wavelength λ

$\varepsilon(\lambda)$, represents the molar absorption coefficient at the wavelength λ, in $mol \cdot L^{-1} \cdot cm^{-1}$

l, the distance of the trajectory in the media, in cm

C, the concentration of the substrate in $mol \cdot L^{-1}$

Table S1: The concentration of ligands.

ligand	solvent	Concentration 1 (mM)	Concentration 2 (mM)
E-**1**	Tris buffer: DMSO (95:5)	2.9	n.d.[c]
Z-**1**	Tris buffer: DMSO (95:5)	3.0	n.d.
E-**2**	Tris buffer: DMSO (9:1)	3.0	2.9
Z-**2**	Tris buffer: DMSO (9:1)	3.0	2.9
E-**3**	Tris buffer: DMSO (9:1)	2.0	n.d.
Z-**3**	Tris buffer: DMSO (9:1)	2.0	n.d.
E-**4**	Tris buffer: DMSO (9:1)	3.0	3.0
Z-**4**	Tris buffer: DMSO (9:1)	3.0	2.9
E-**5**	Tris buffer: DMSO (9:1)	3.0	2.9
Z-**5**	Tris buffer: DMSO (9:1)	3.0	2.9

Concentration 1: The concentration was calculated from weighted mass; Concentration 2: The concentration was calculated by spectroscopy; [c] not determined.

Copies of ^{1}H and $^{13}C\{^{1}H\}$ NMR spectra

^{1}H NMR of compound *E*-1 in CD_3OD at 400 MHz

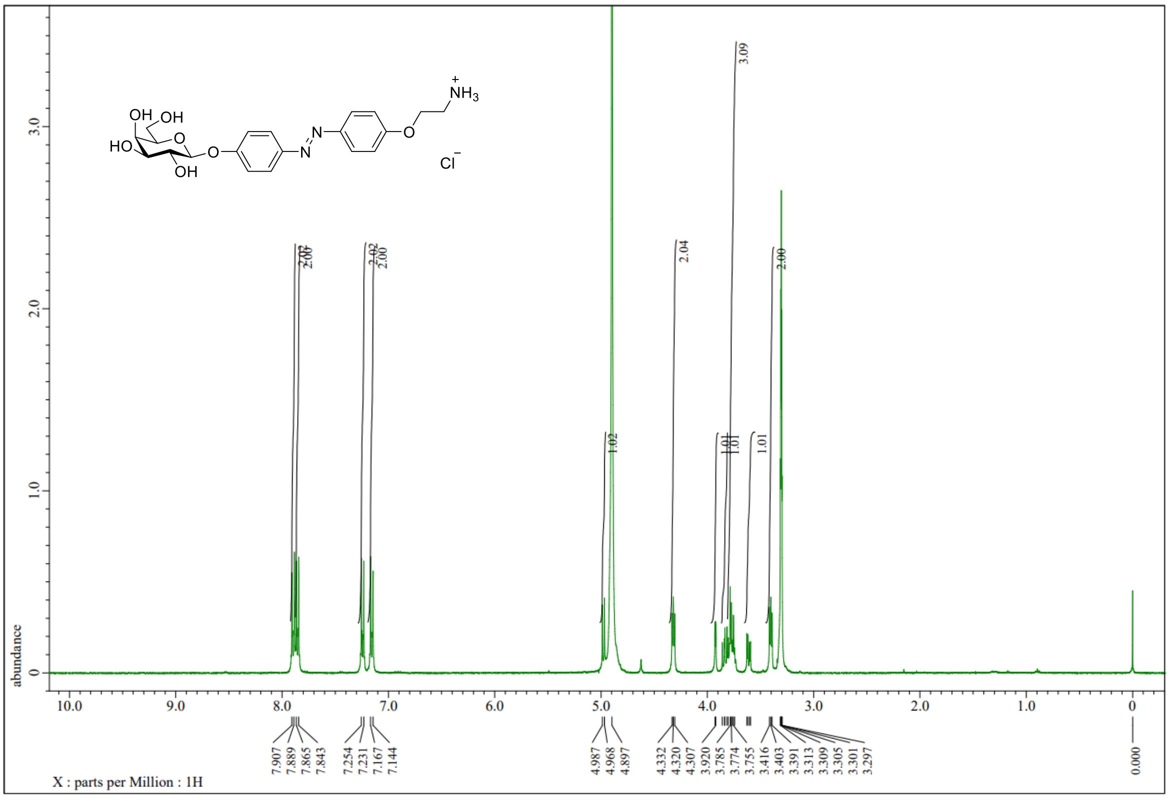

Enlarged ^{1}H NMR of compound *E*-1 in CD_3OD at 400 MHz

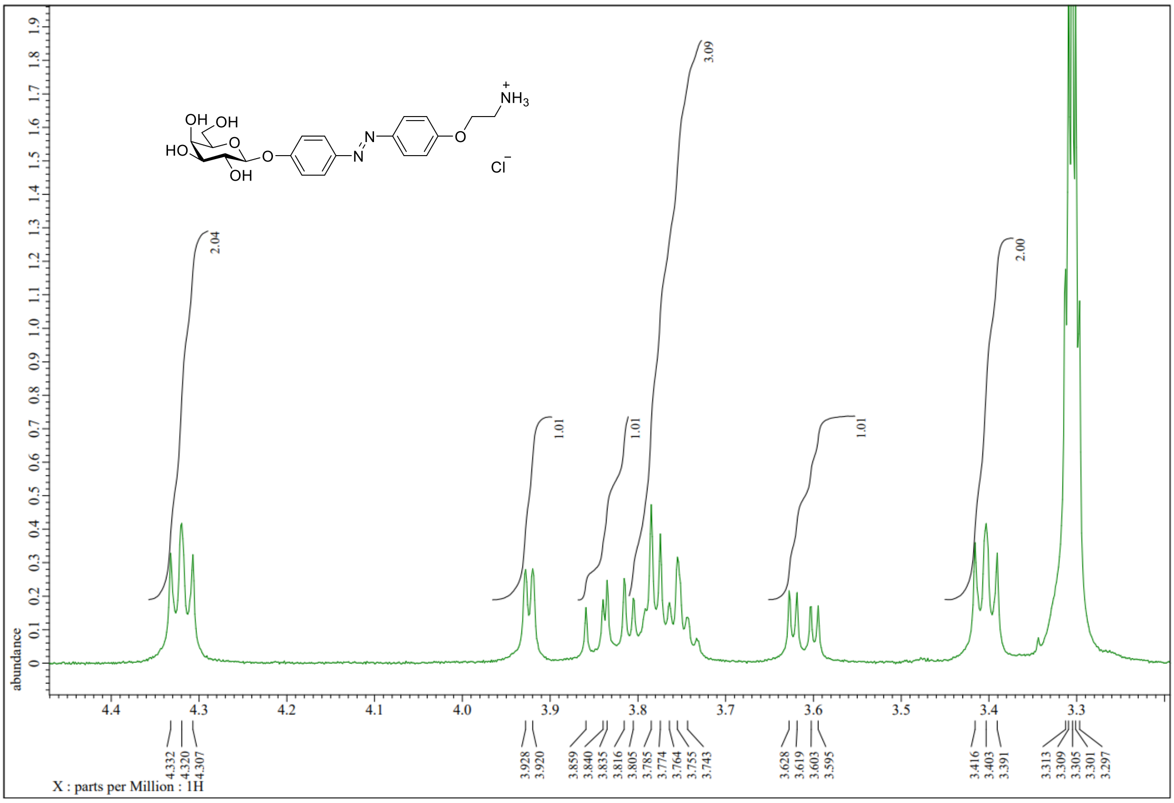

$^{13}C\{^1H\}$ NMR (UDEFT) of compound *E*-1 in CD_3OD at 100 MHz

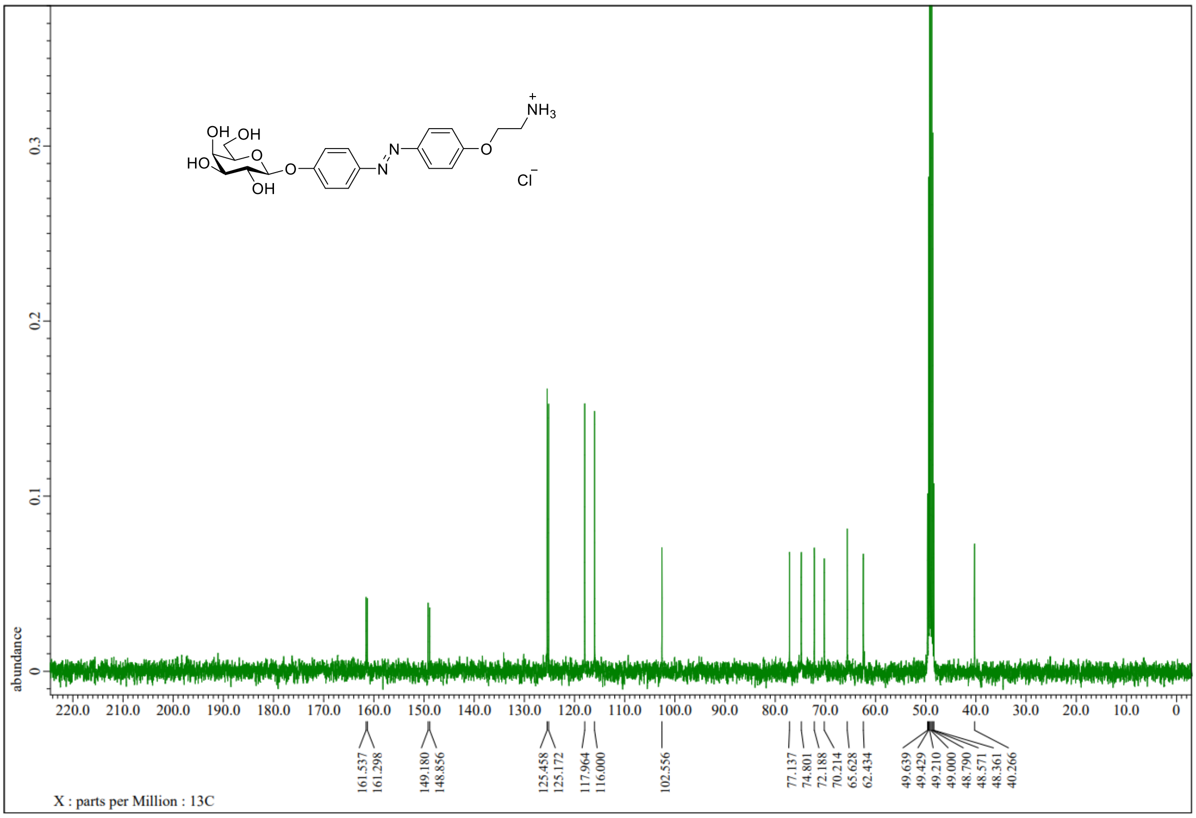

^{1}H NMR of compound *E*-2 in CD_3OD at 400 MHz

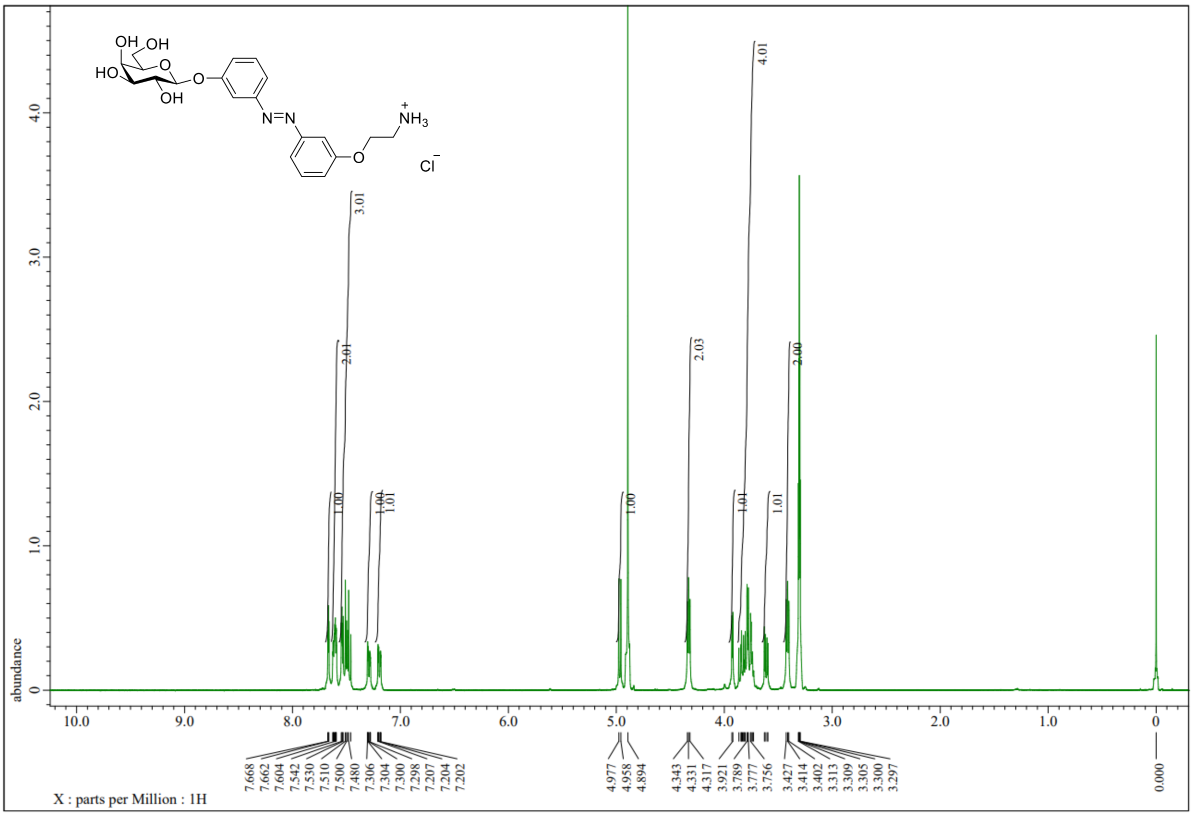

Enlarged ^{1}H NMR of compound *E*-2 in CD_3OD at 400 MHz

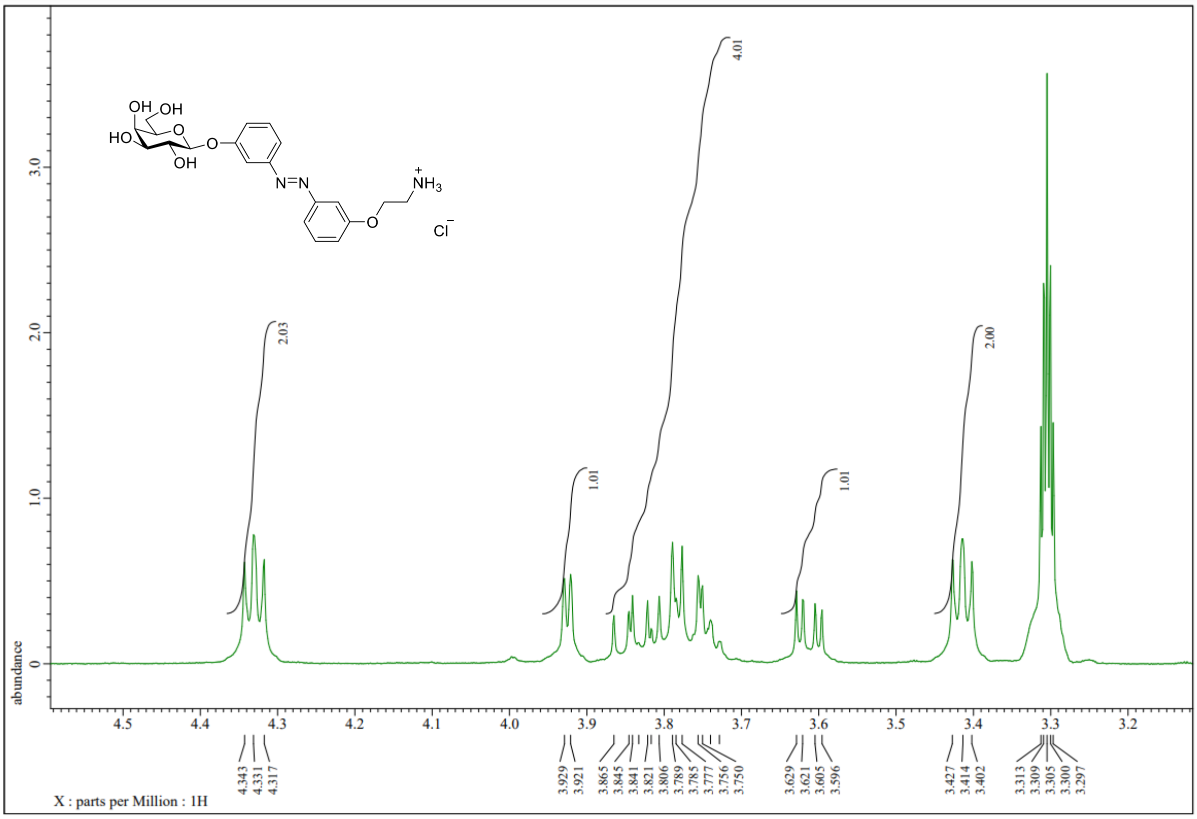

$^{13}C\{^{1}H\}$ NMR of compound *E*-2 in CD_3OD at 100 MHz

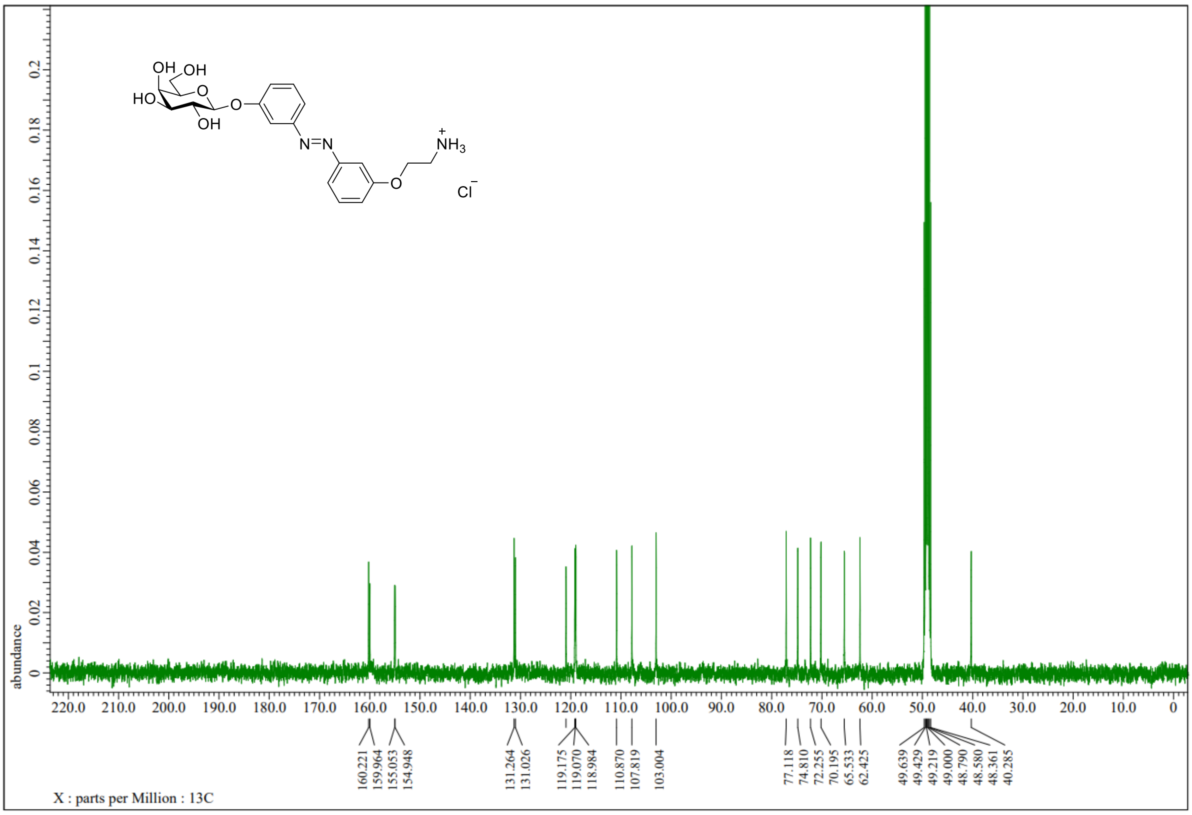

^{1}H NMR of compound *E*-3 in $CDCl_3$ at 400 MHz

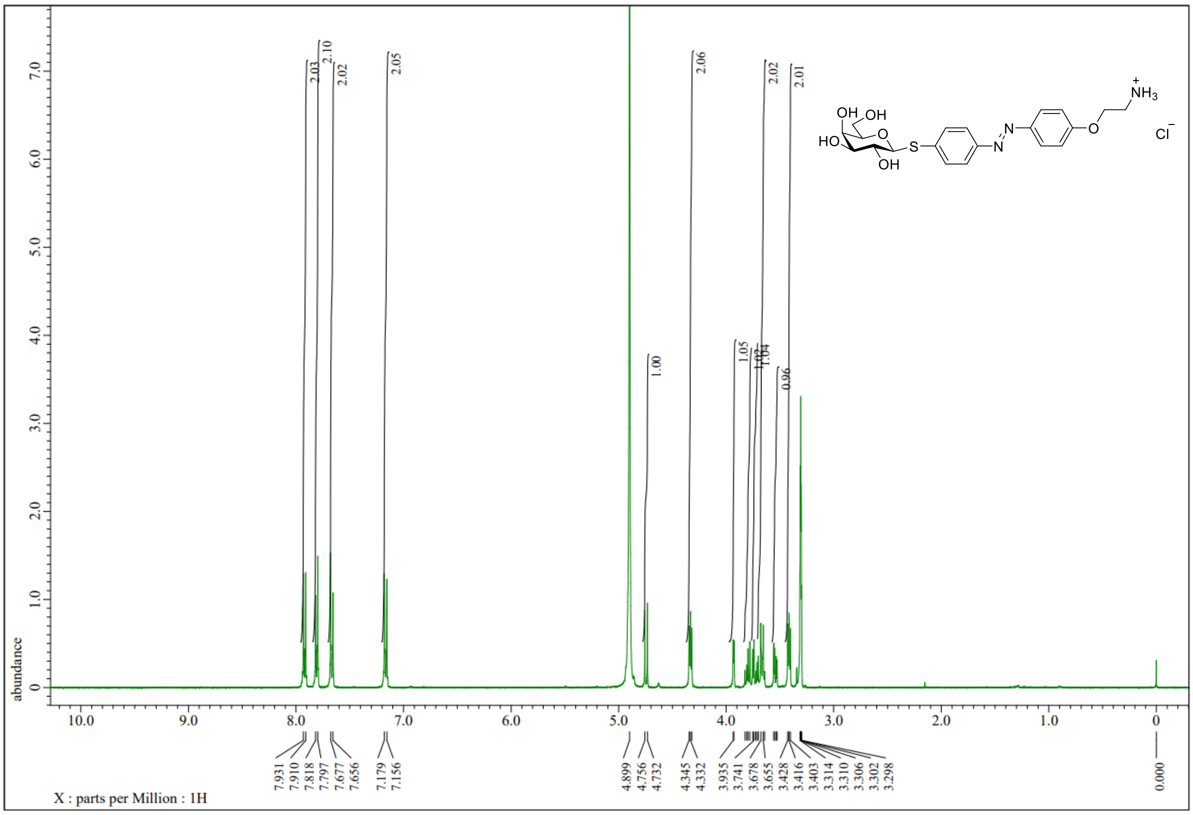

Enlarged ^{1}H NMR of compound *E*-3 in $CDCl_3$ at 400 MHz

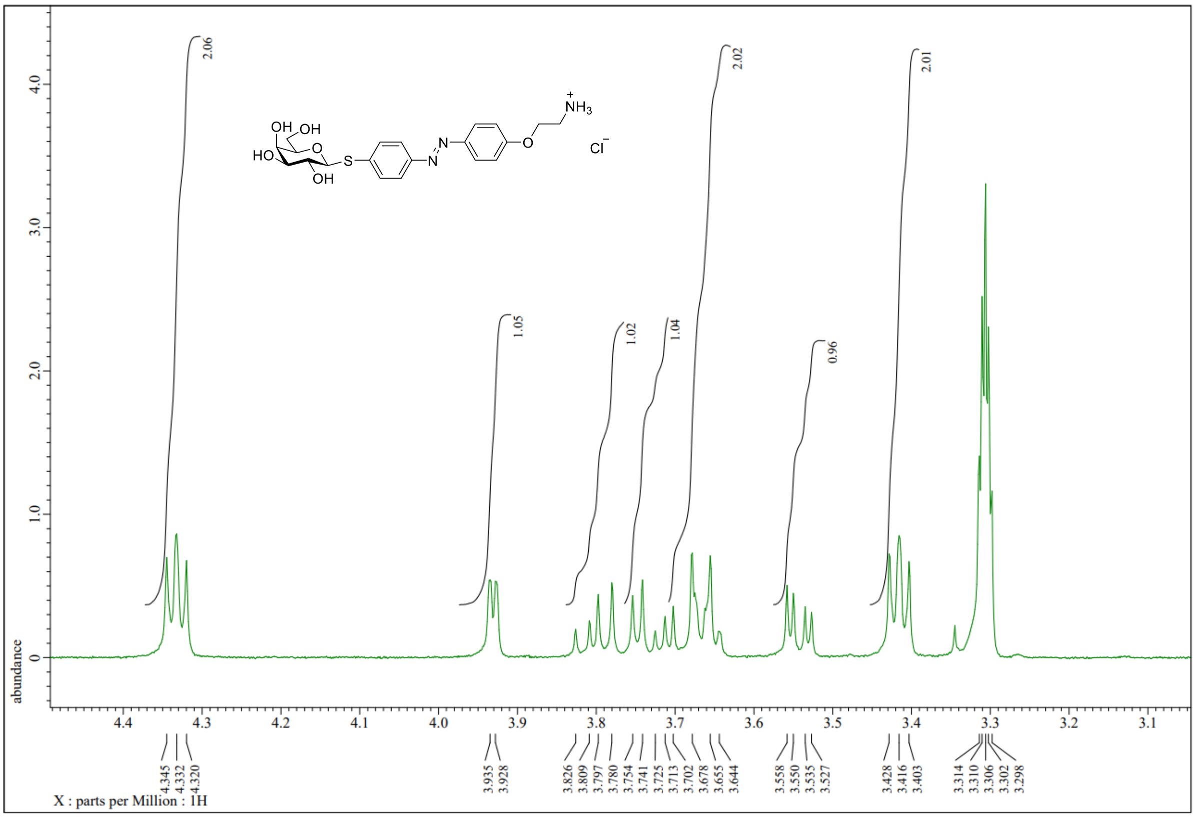

$^{13}C\{^{1}H\}$ NMR (UDEFT) of compound *E*-3 in $CDCl_3$ at 100 MHz

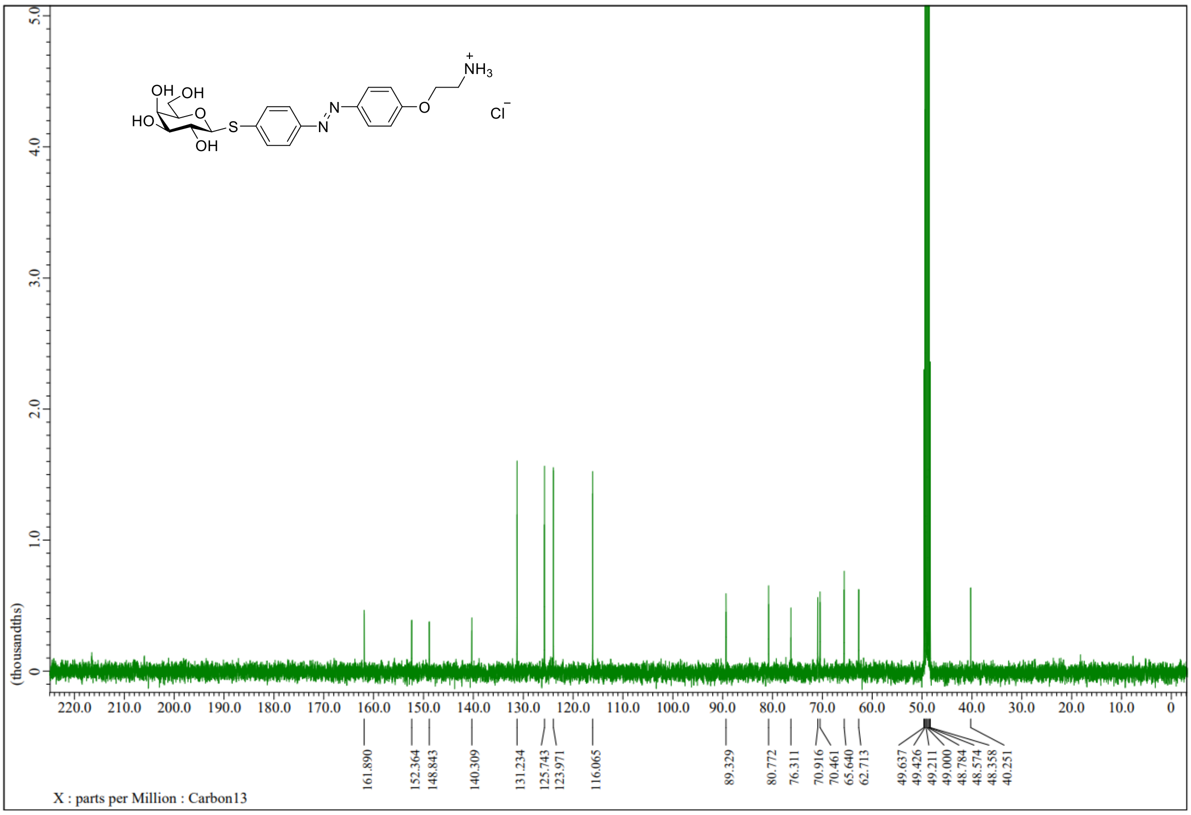

^{1}H NMR of compound *E*-4 in CD_3OD at 400 MHz

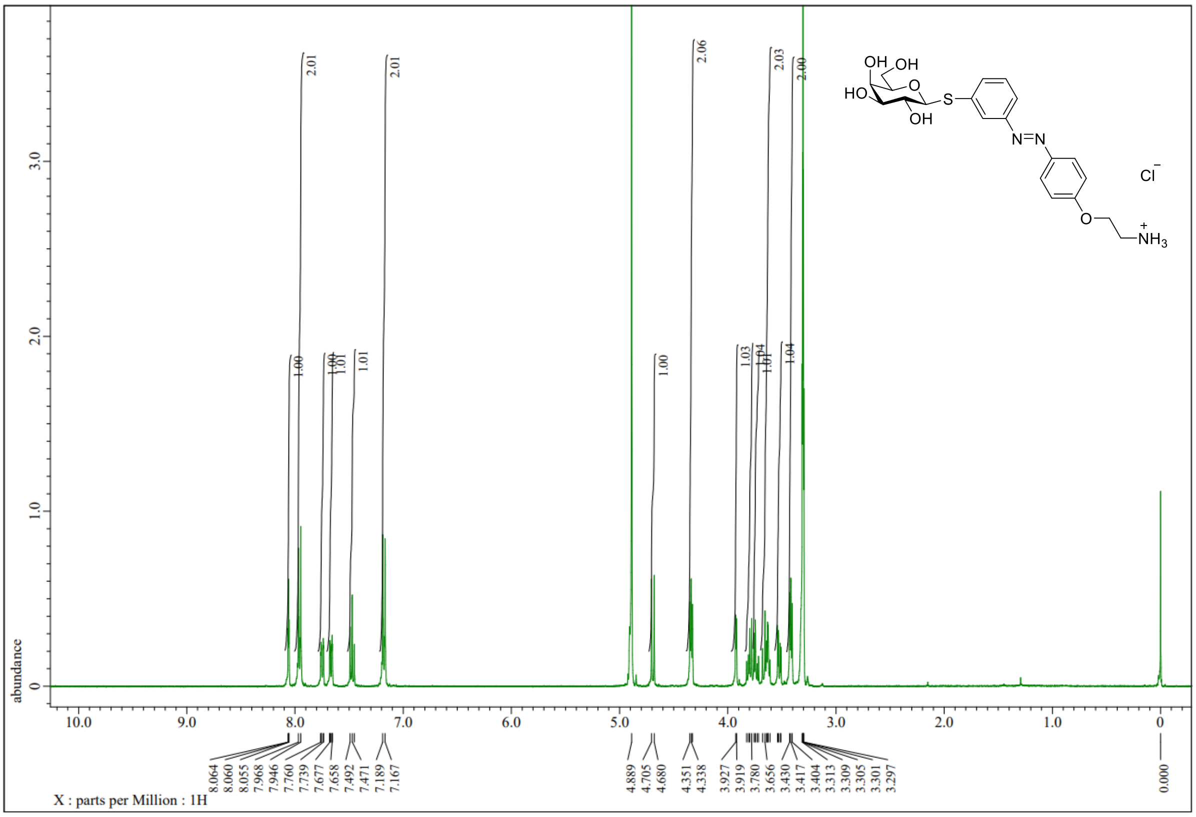

Enlarged ^{1}H NMR of compound *E*-4 in CD_3OD at 400 MHz

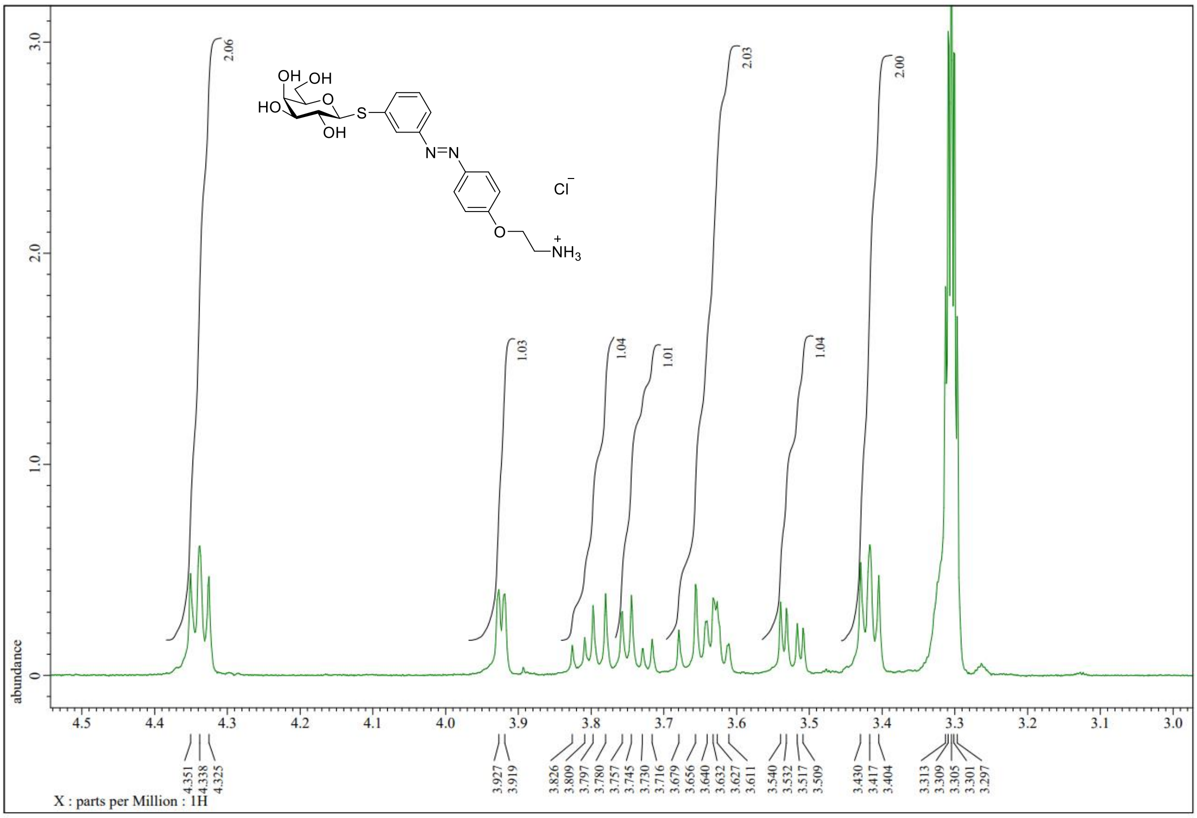

$^{13}C\{^{1}H\}$ NMR of compound *E*-4 in CD_3OD at 100 MHz

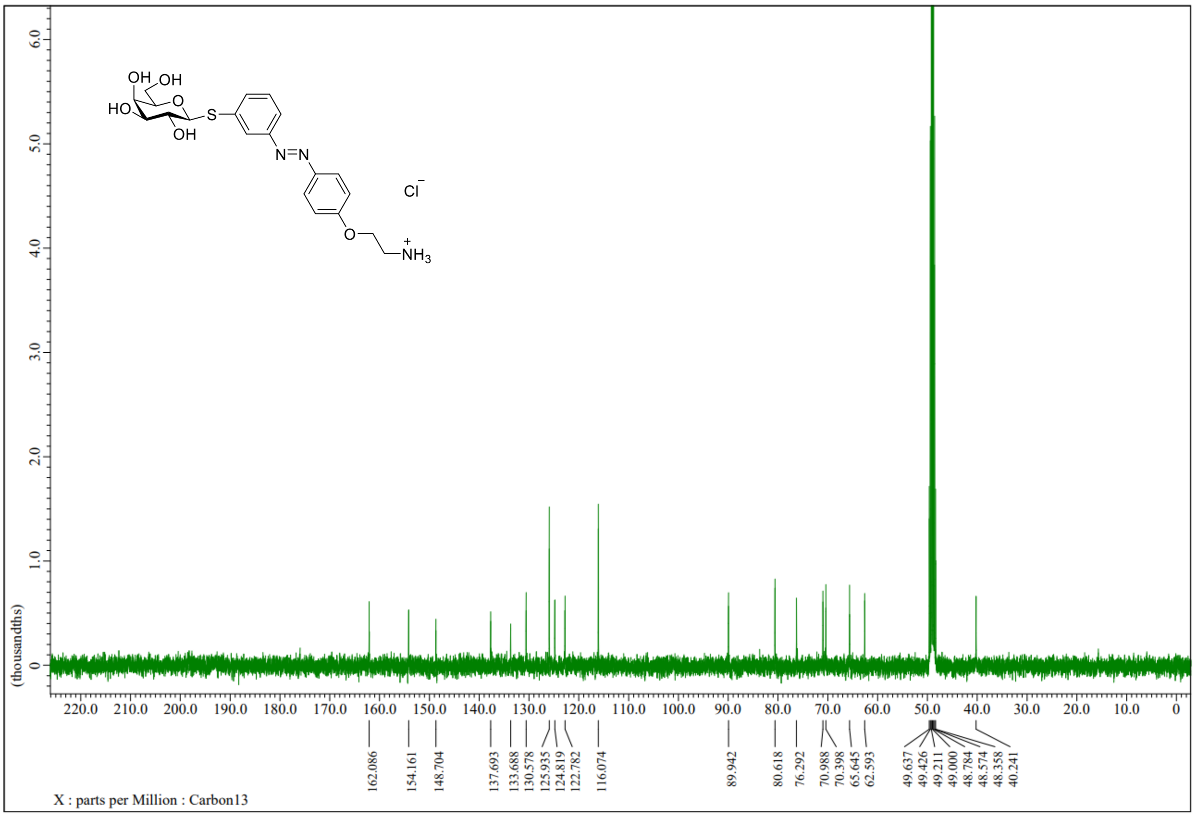

^{1}H NMR of compound *E*-5 in CD3OD at 400 MHz

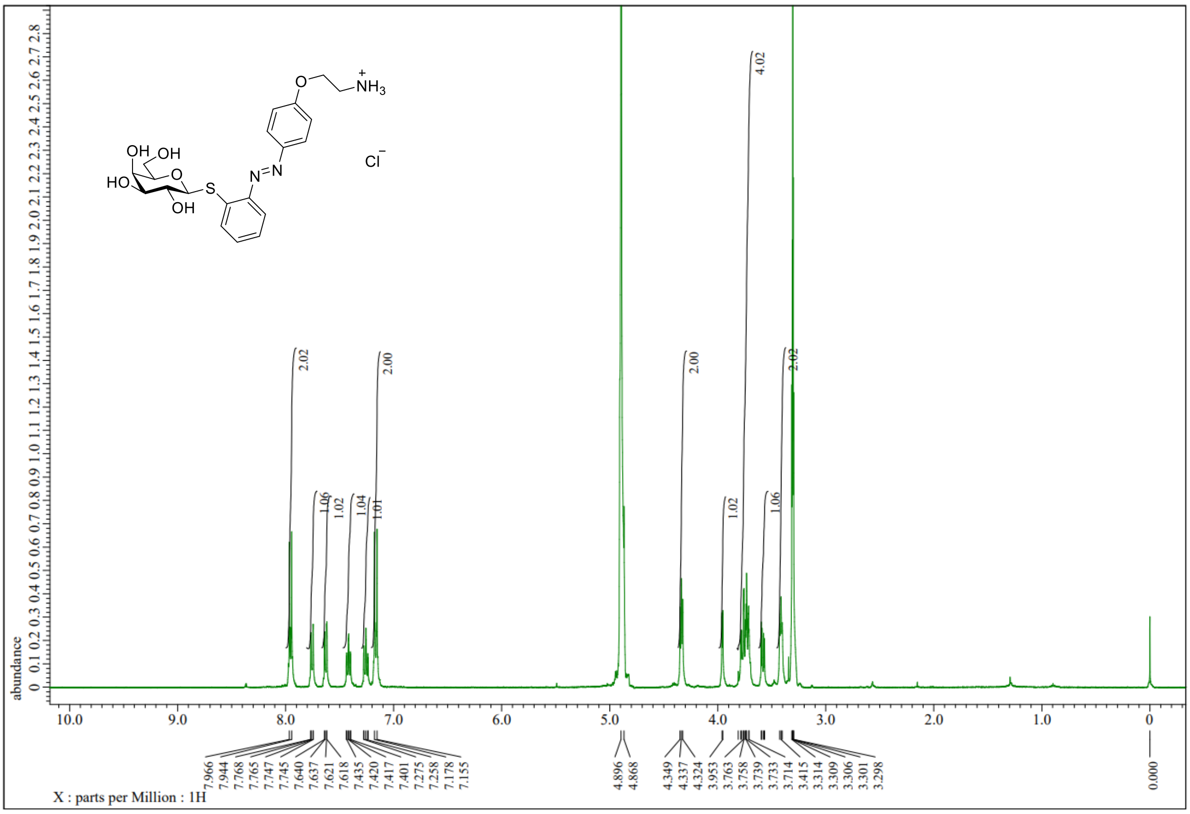

Enlarged ^{1}H NMR of compound *E*-5 in CD3OD at 400 MHz

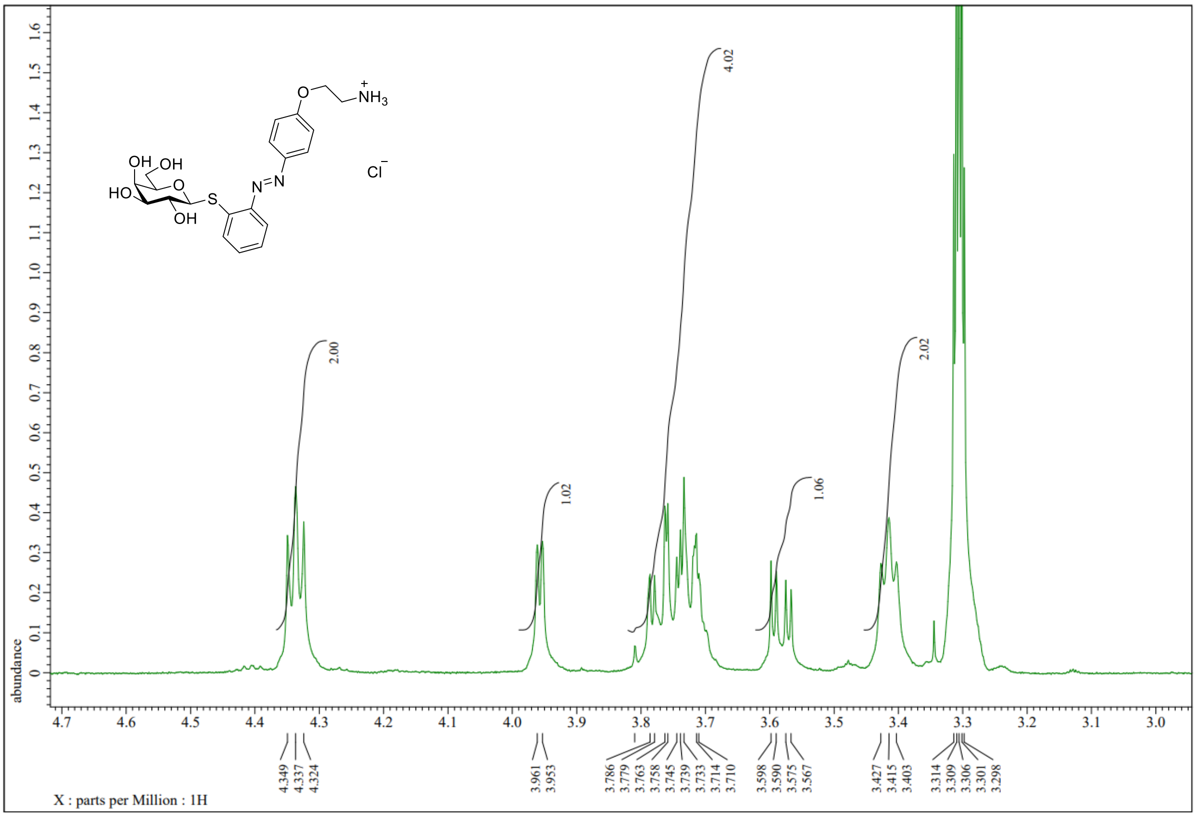

$^{13}C\{^{1}H\}$ NMR of compound *E*-5 in CD_3OD at 100 MHz

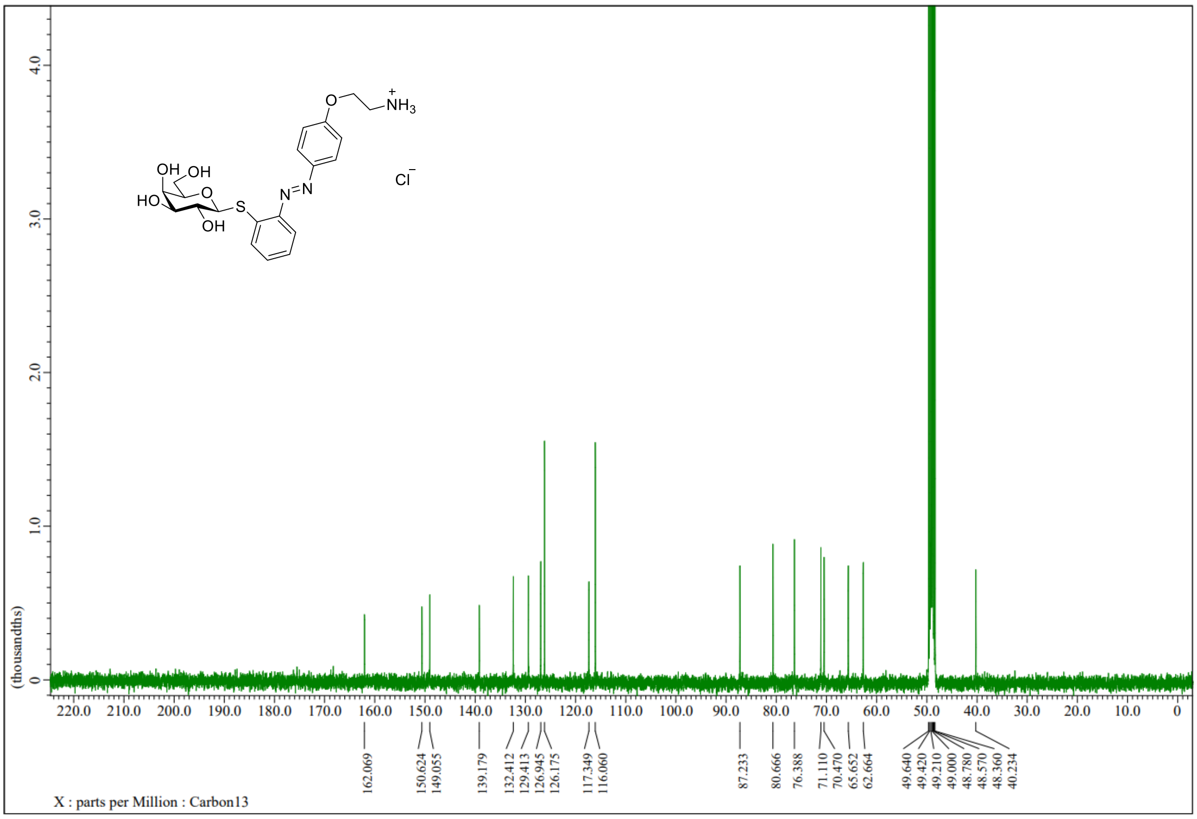

^{1}H NMR of compound *E*-8 in CD_3OD at 400 MHz

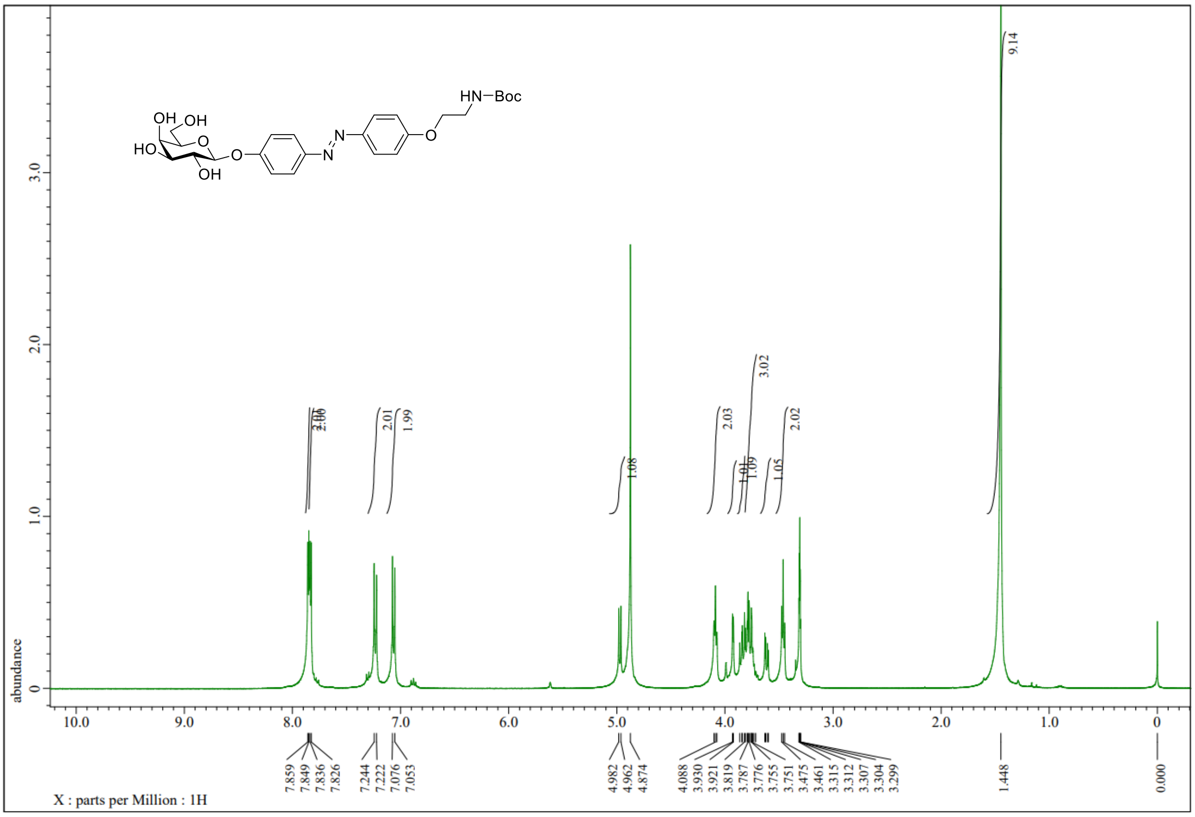

Enlarged ^{1}H NMR of compound *E*-8 in CD_3OD at 400 MHz

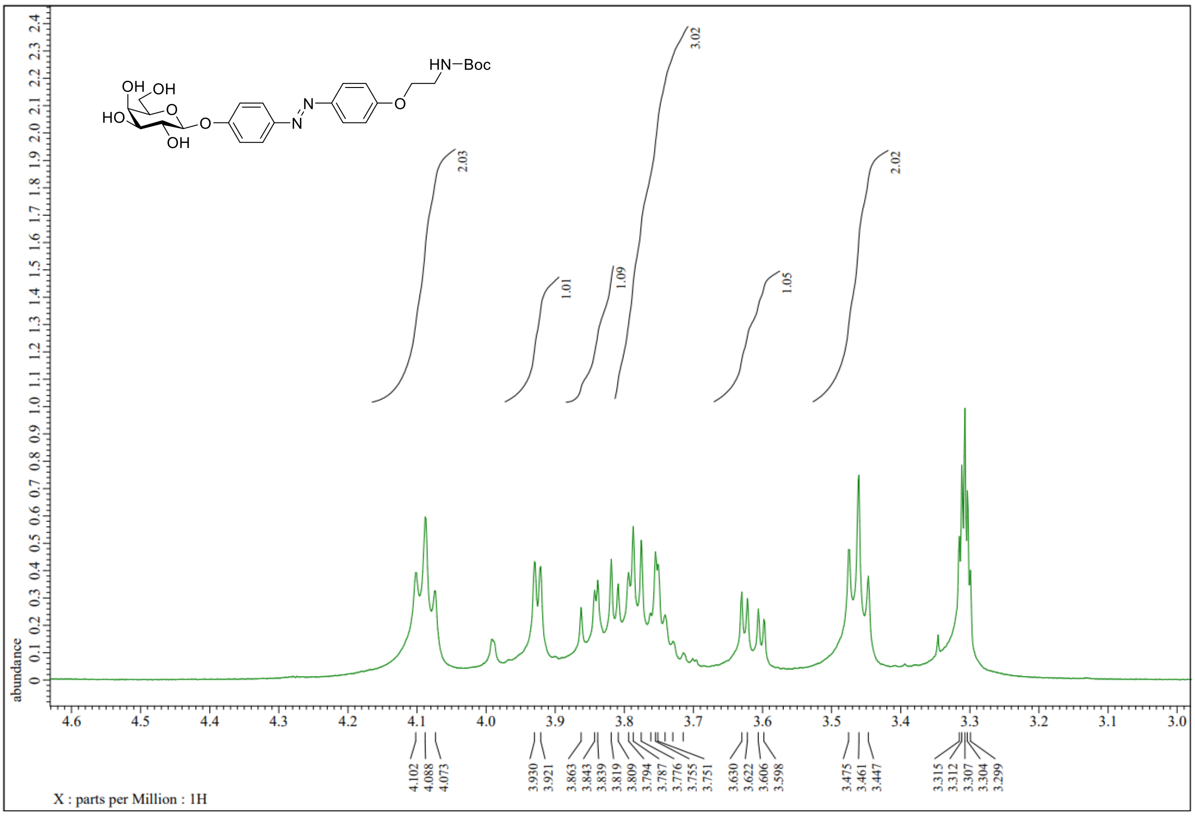

$^{13}C\{^1H\}$ NMR (UDEFT) of compound *E*-8 in CD_3OD at 100 MHz

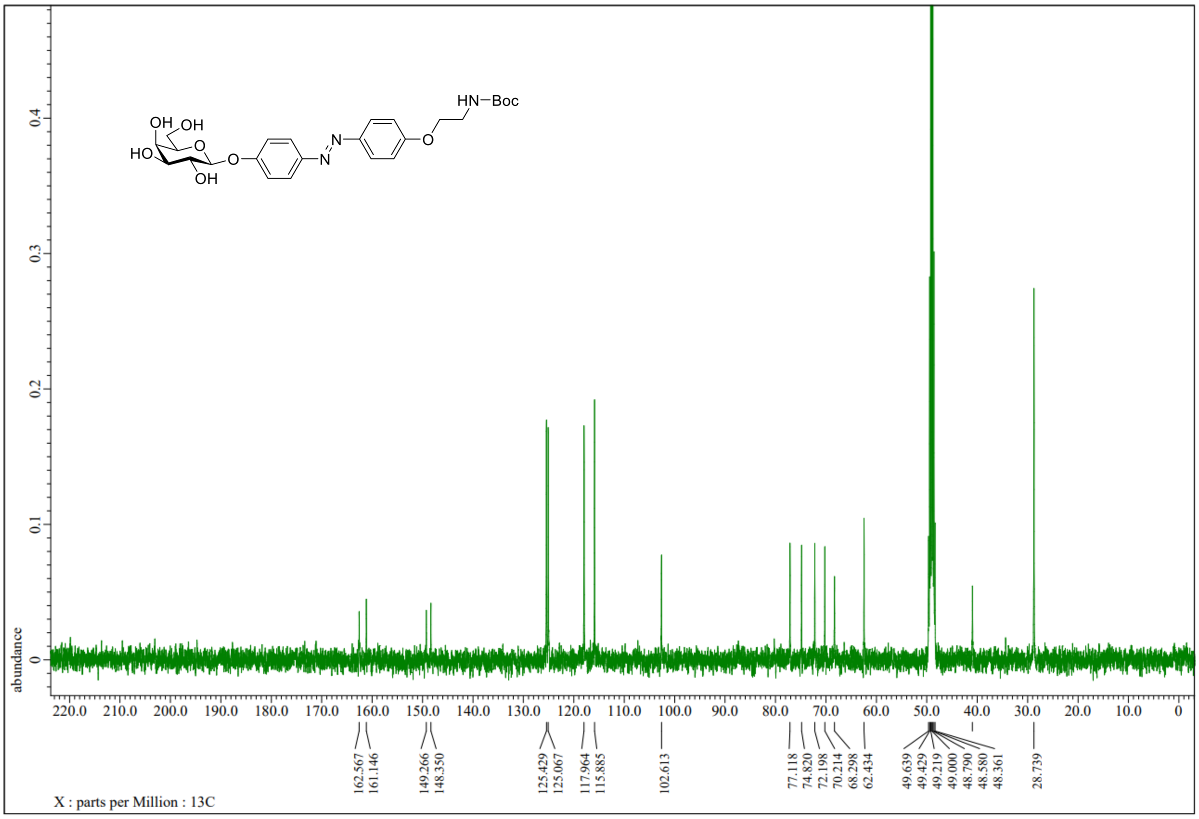

1H NMR of compound *E*-10 in CD_3OD at 400 MHz

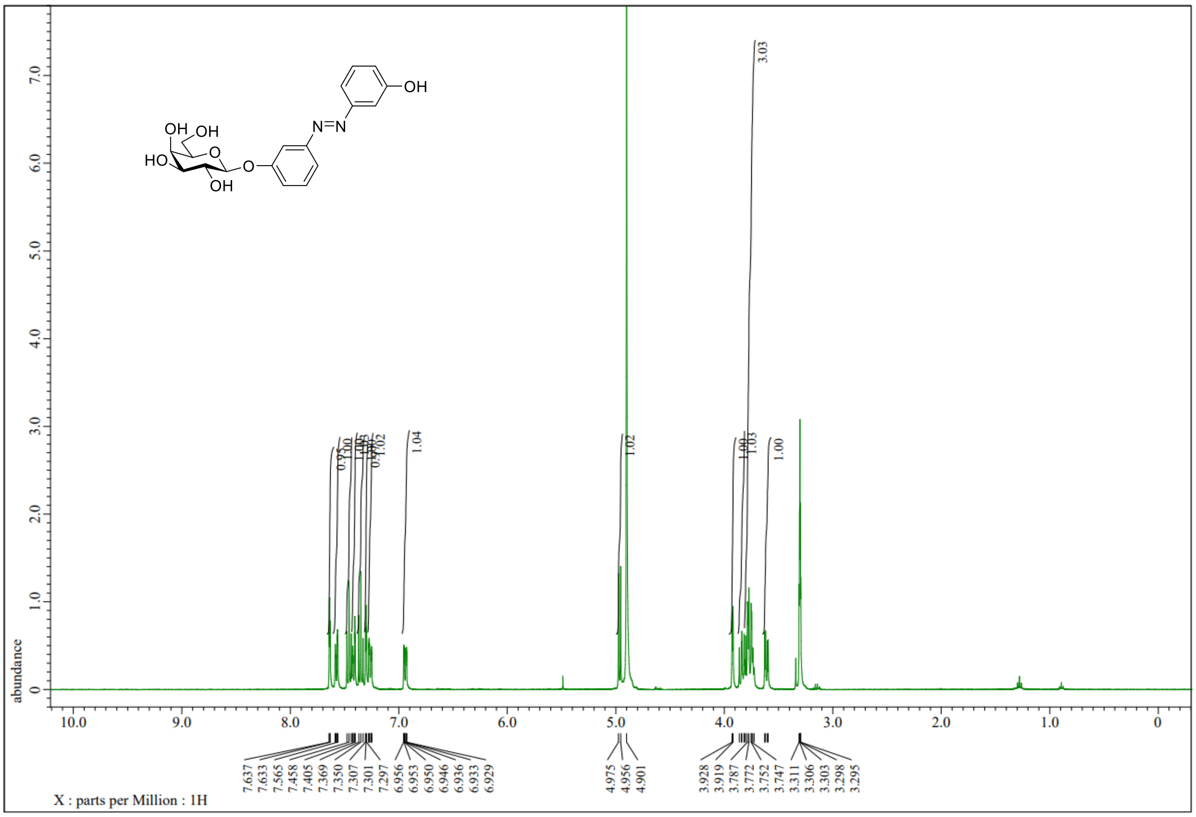

Enlarged 1H NMR of compound *E*-10 in CD_3OD at 400 MHz

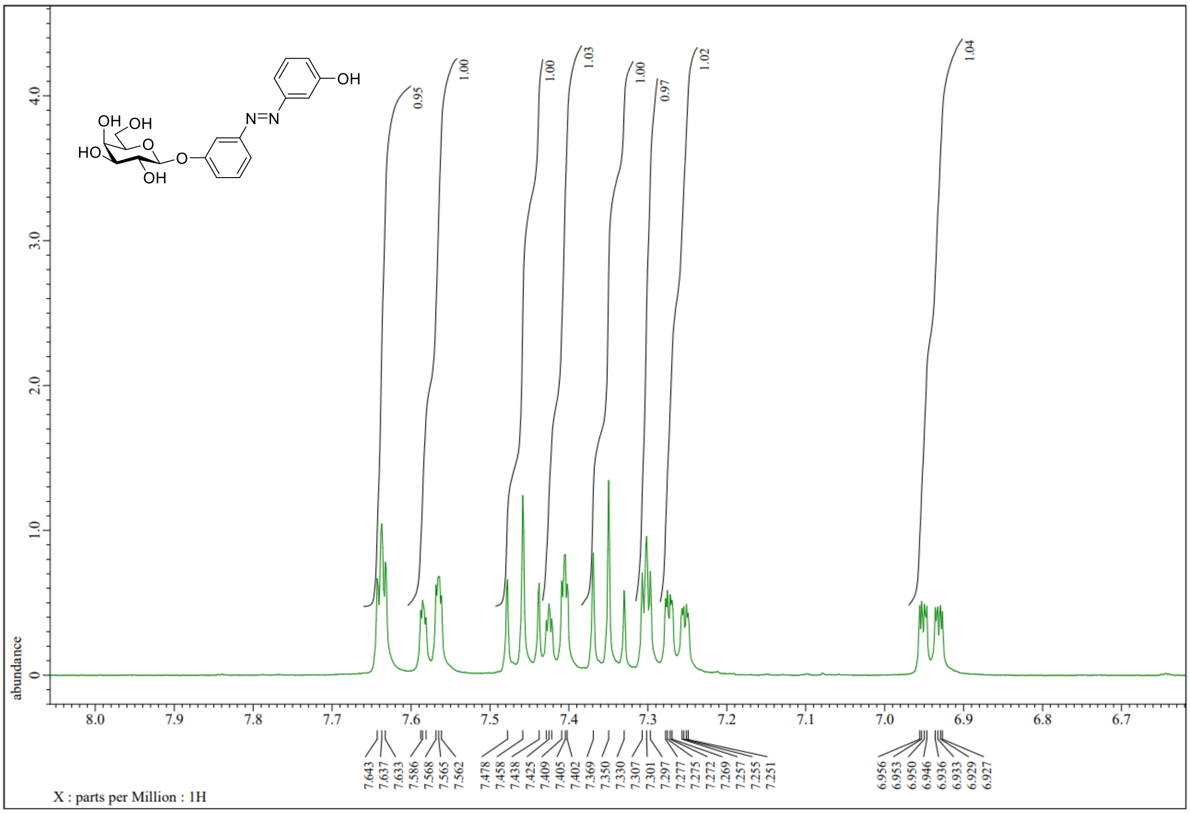

$^{13}C\{^1H\}$ NMR of compound *E*-10 in CD_3OD at 100 MHz

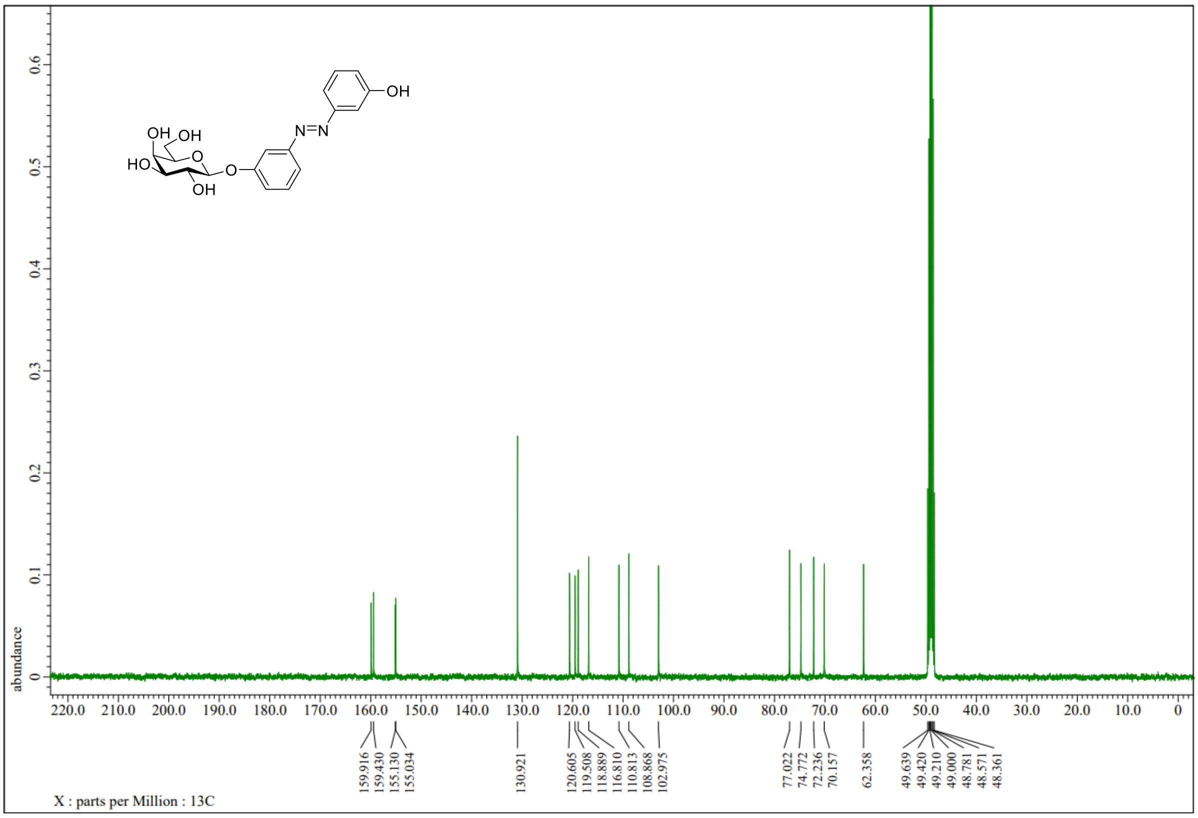

^{1}H NMR of compound *E*-11 in CD_3OD at 400 MHz

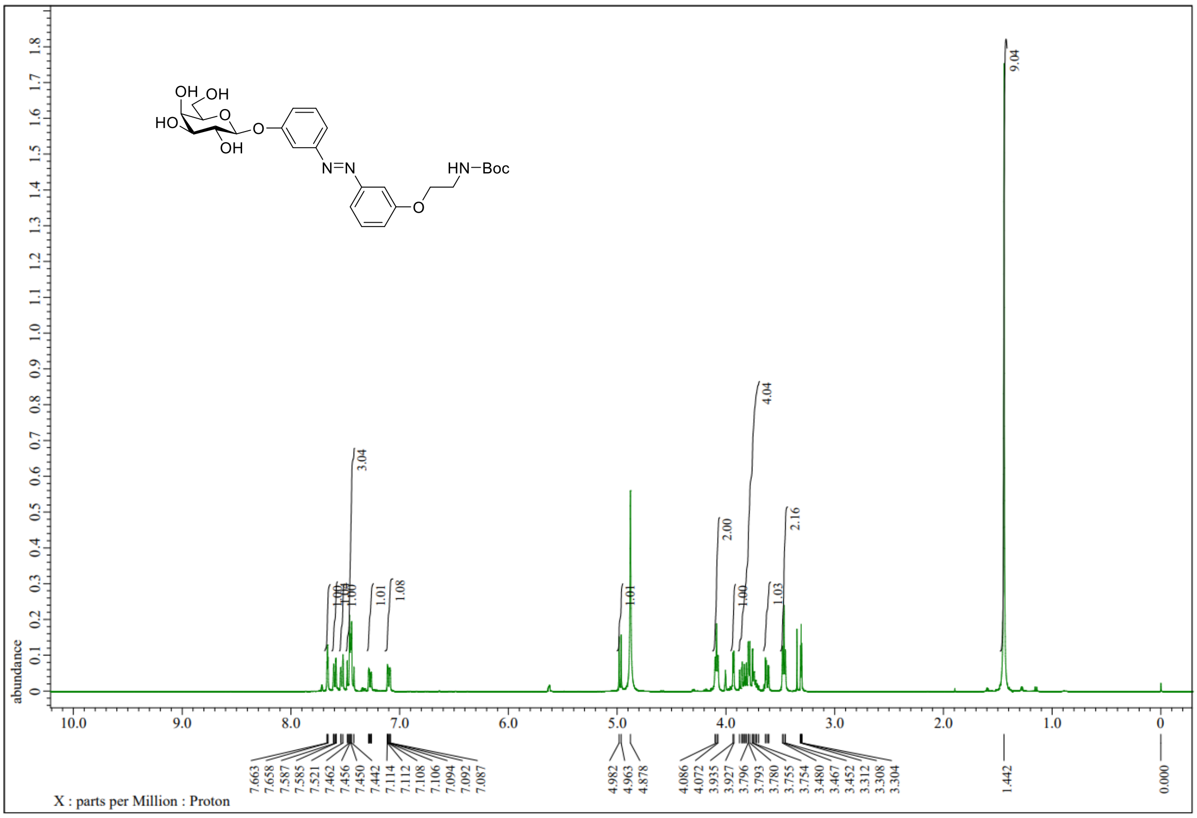

Enlarged ^{1}H NMR of compound *E*-11 in CD_3OD at 400 MHz

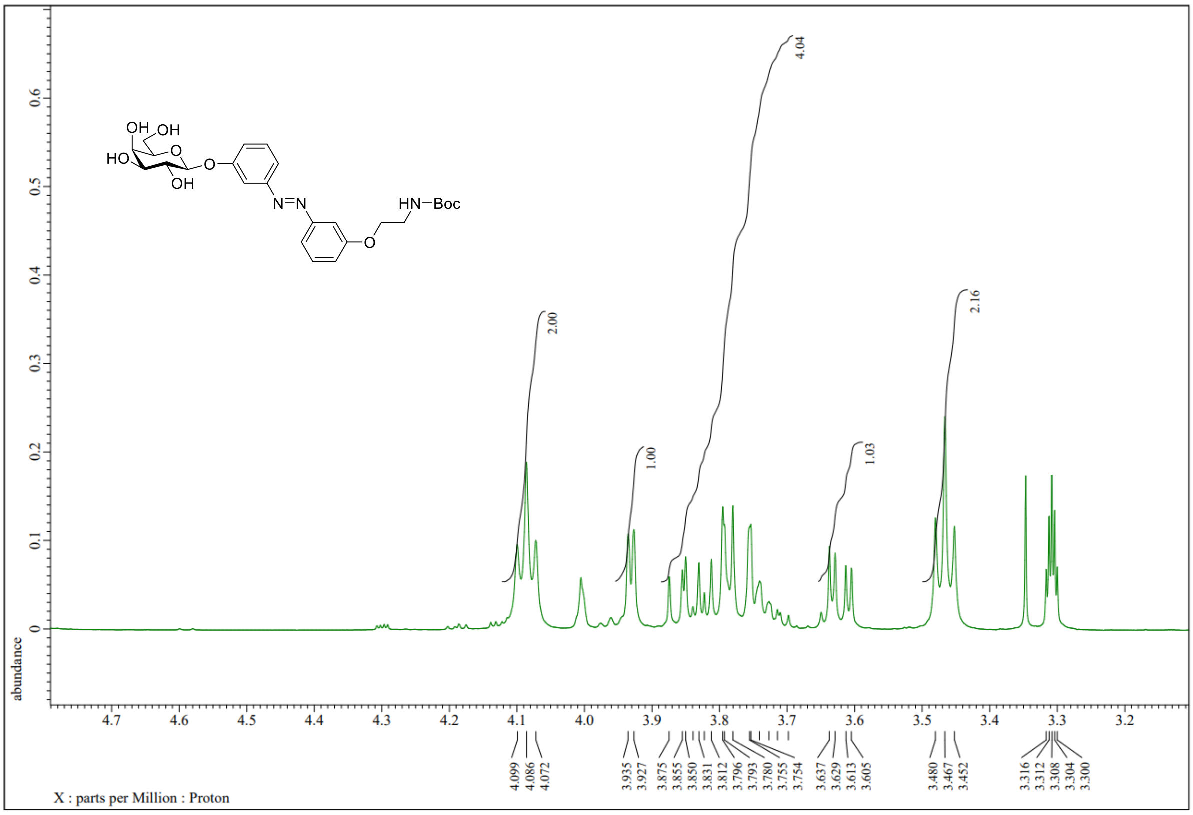

$^{13}C\{^{1}H\}$ NMR of compound *E*-11 in CD_3OD at 100 MHz

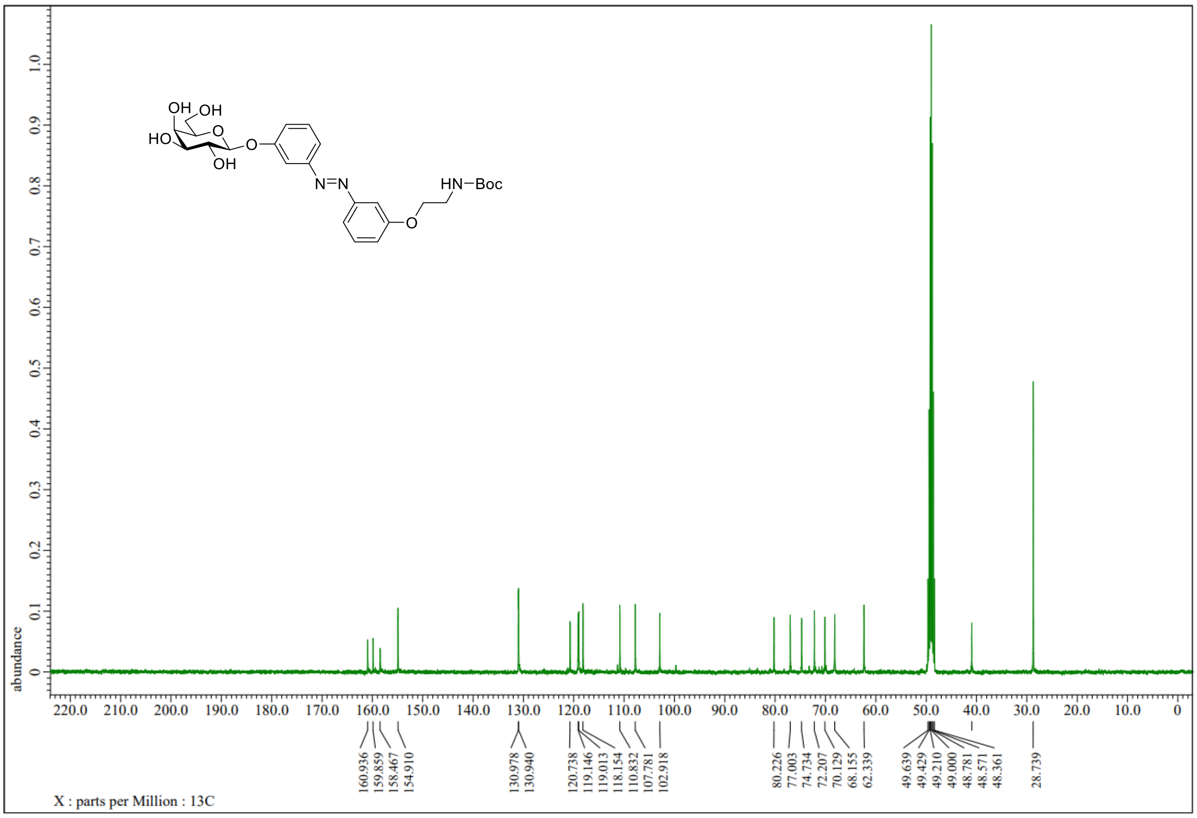

^{1}H NMR of compound *E*-14 in CD_3OD at 400 MHz

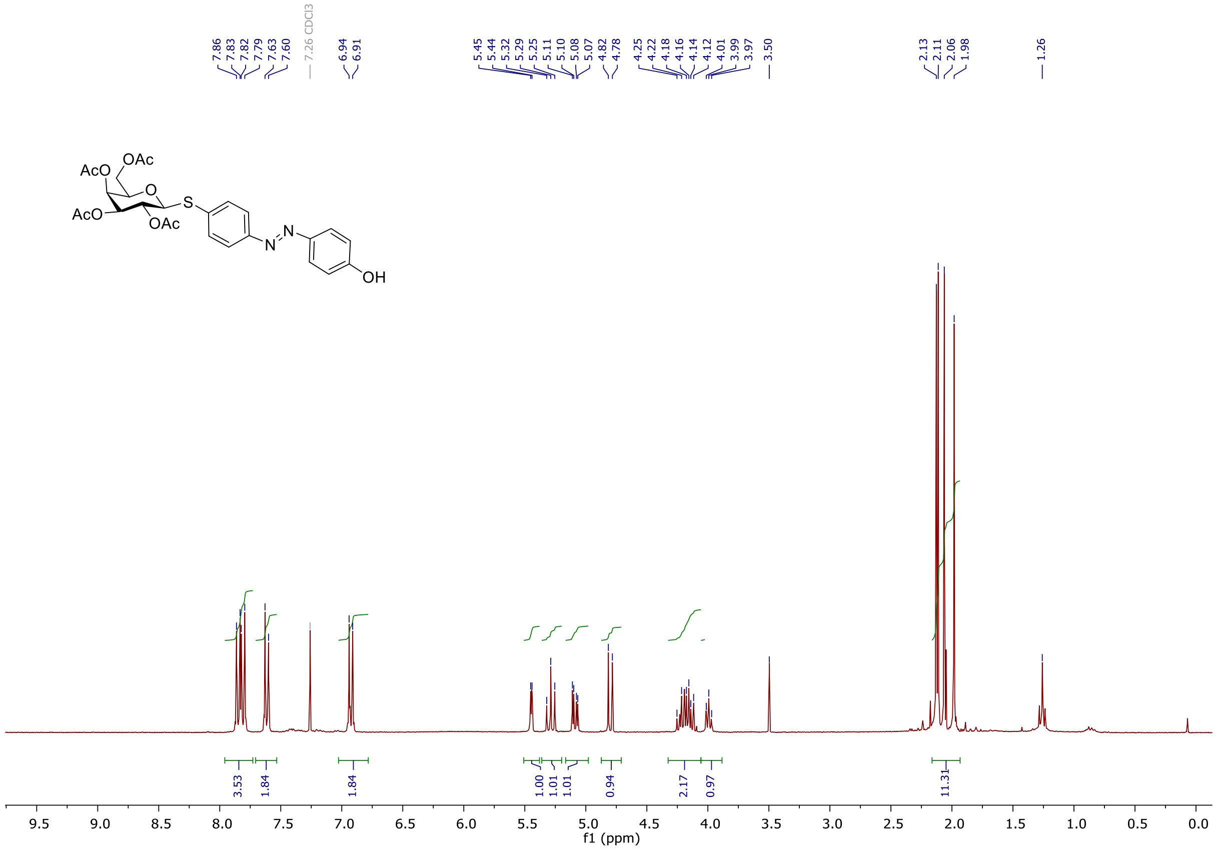

^{13}C{^{1}H} NMR of compound *E*-14 in CD_3OD at 100 MHz

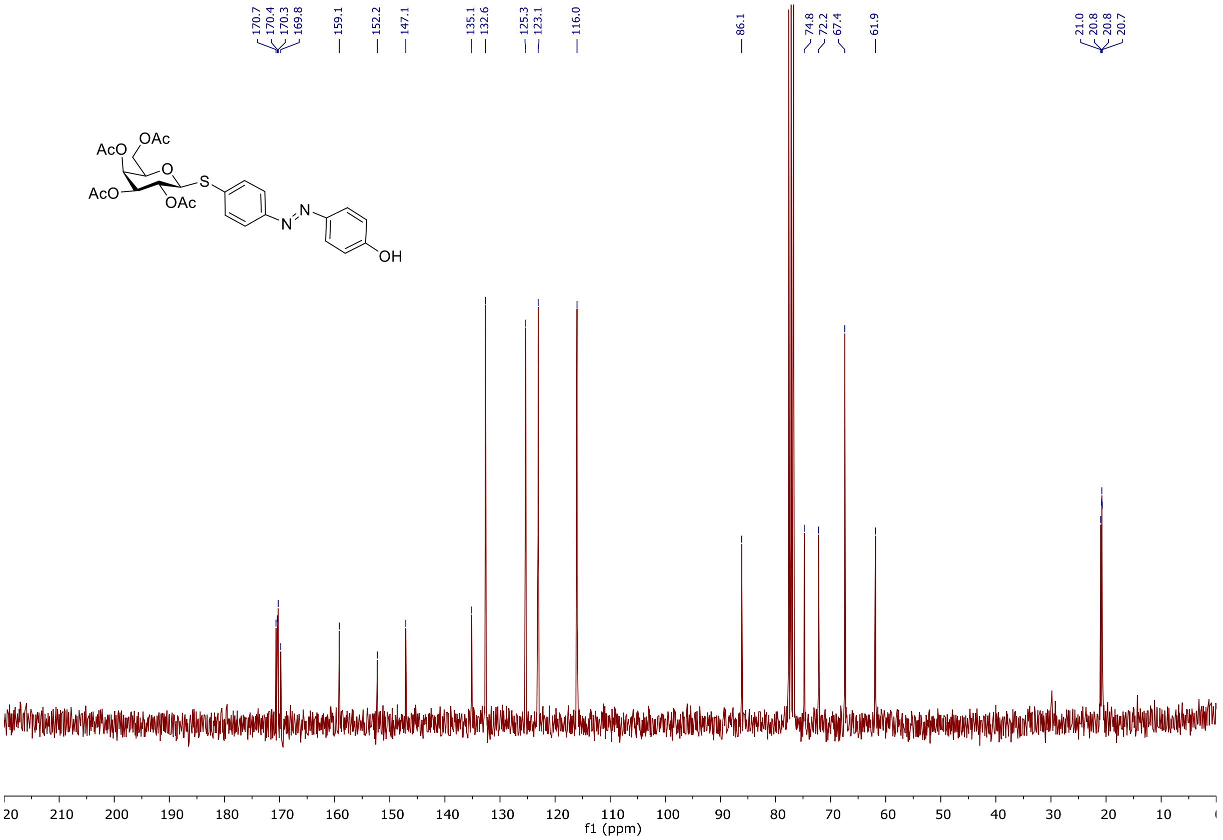

^{1}H NMR of compound *E*-15 in CD_3OD at 300 MHz

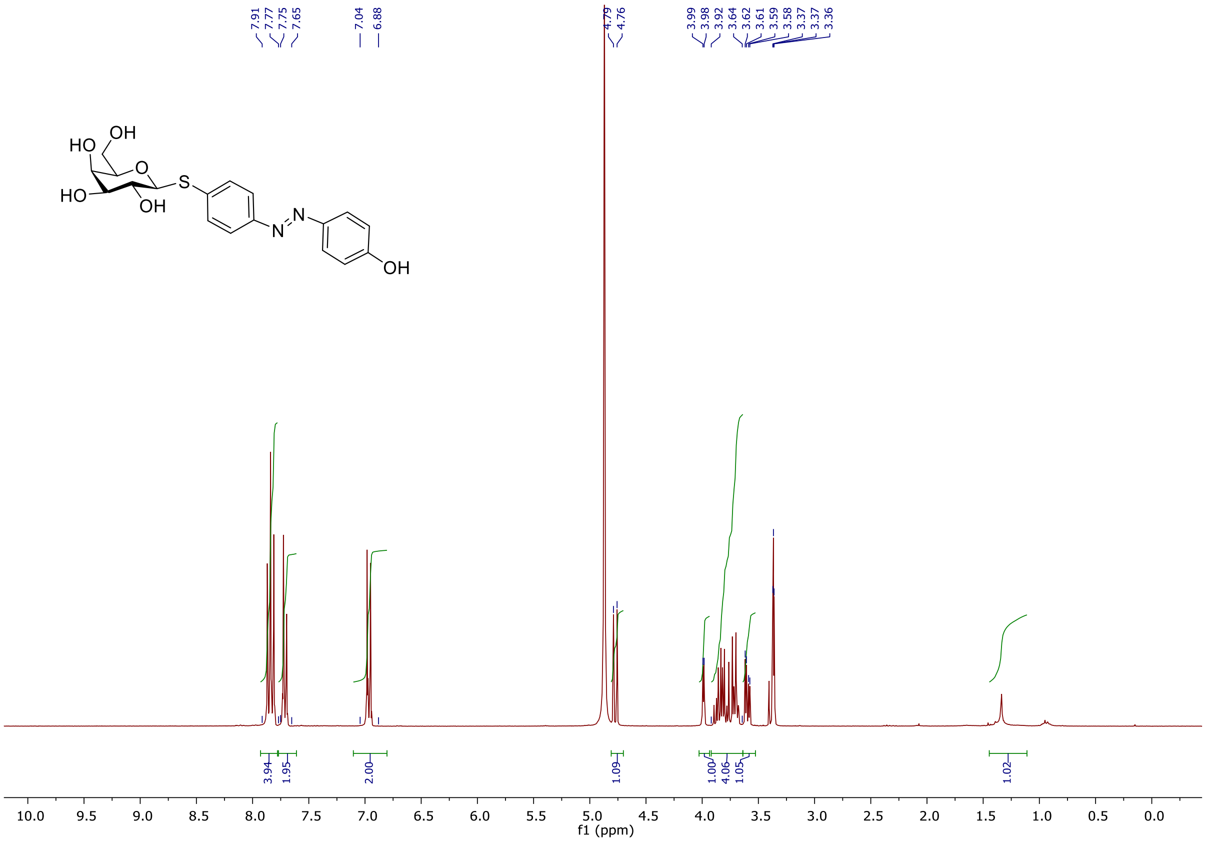

$^{13}C\{^{1}H\}$ NMR of compound *E*-15 in $CDCl_3$ at 75 MHz

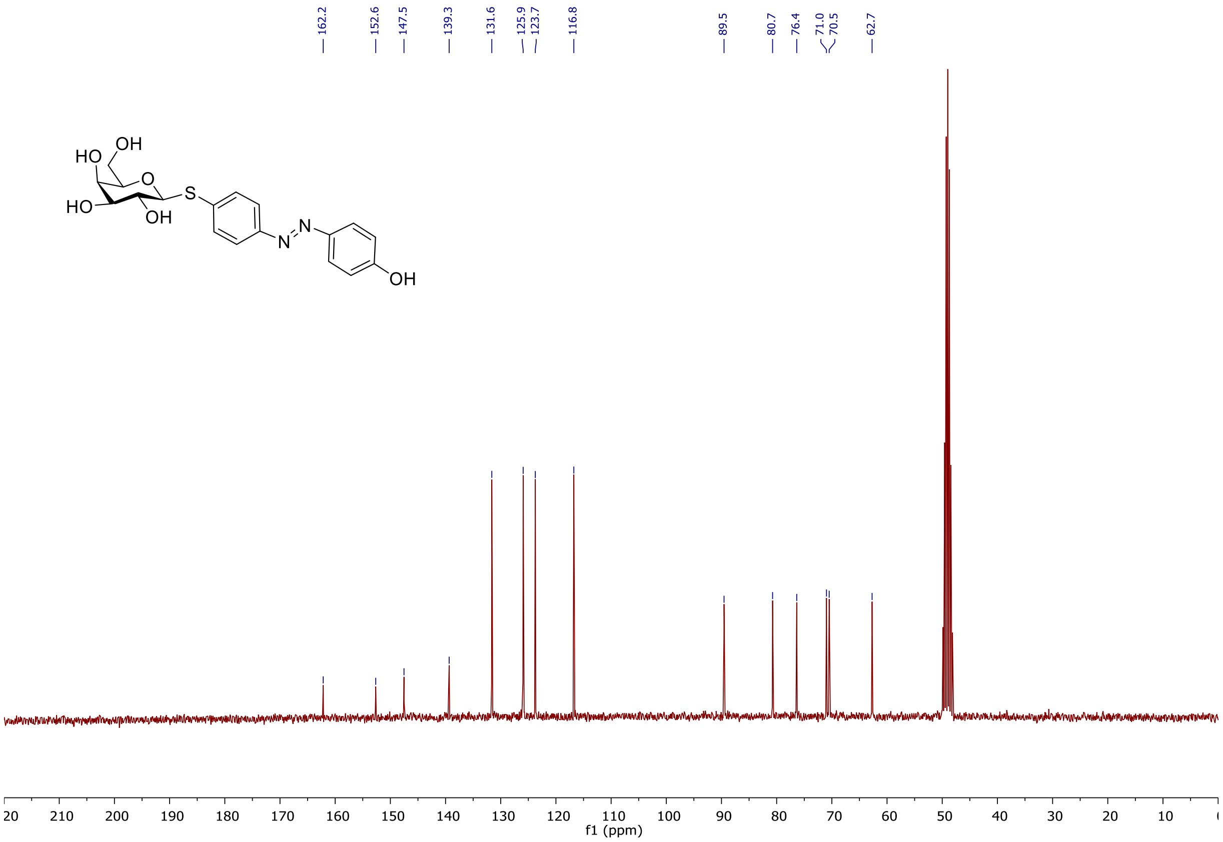

^{1}H NMR of compound *E*-16 in CD_3OD at 400 MHz

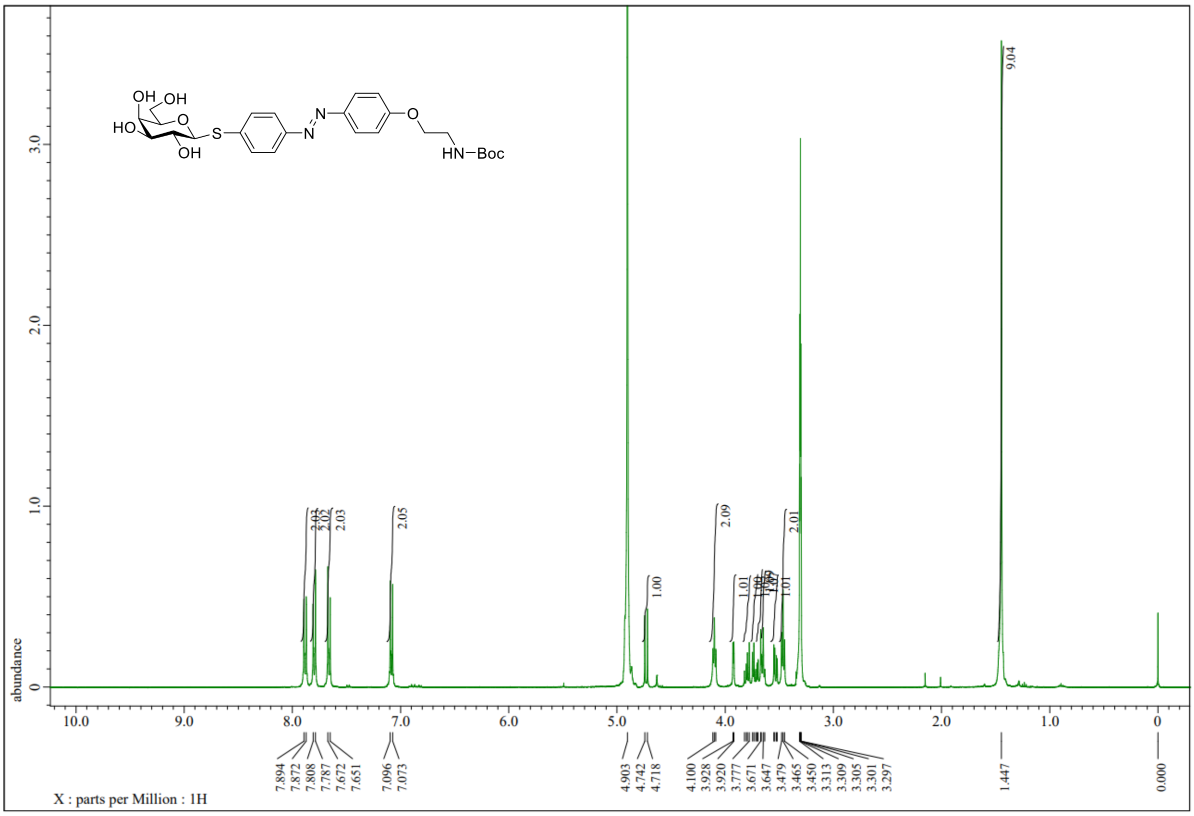

Enlarged ^{1}H NMR of compound *E*-16 in CD_3OD at 400 MHz

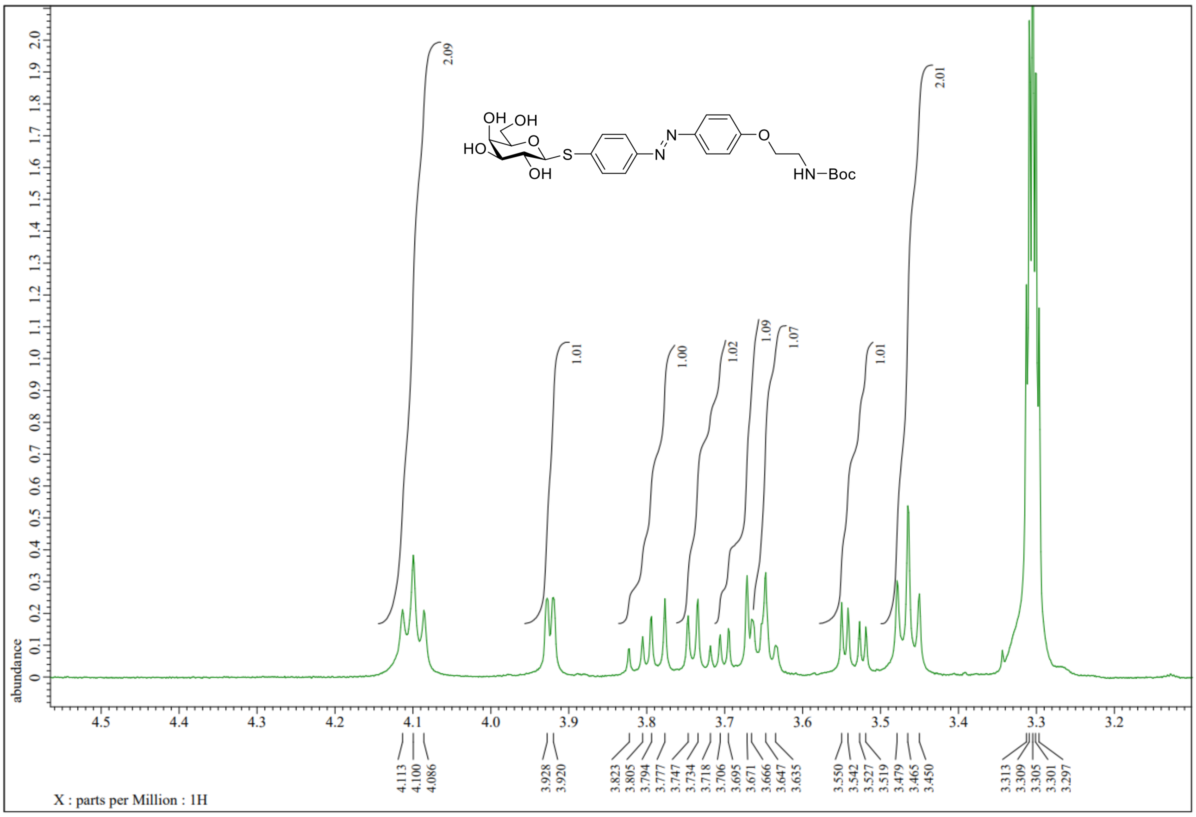

$^{13}C\{^{1}H\}$ NMR of compound *E*-16 in CD_3OD at 100 MHz

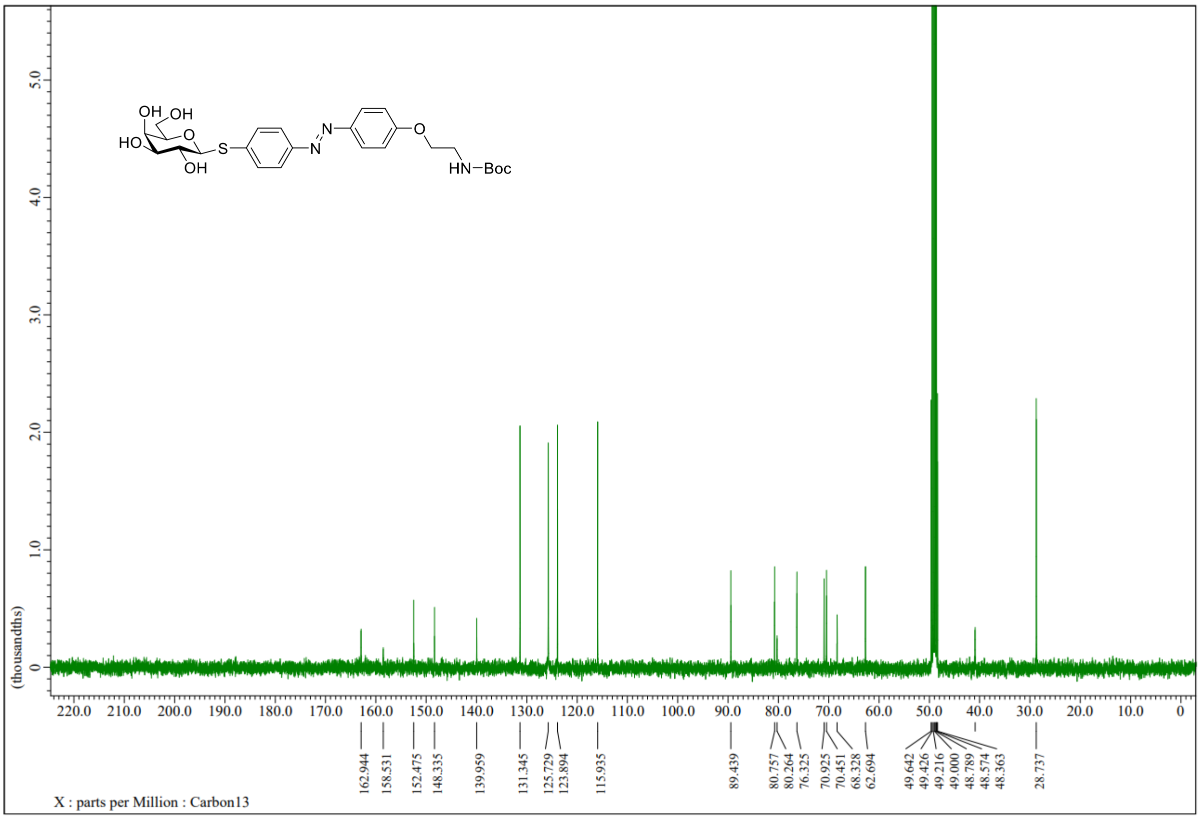

^{1}H NMR of compound *E*-18 in $CDCl_3$ at 300 MHz

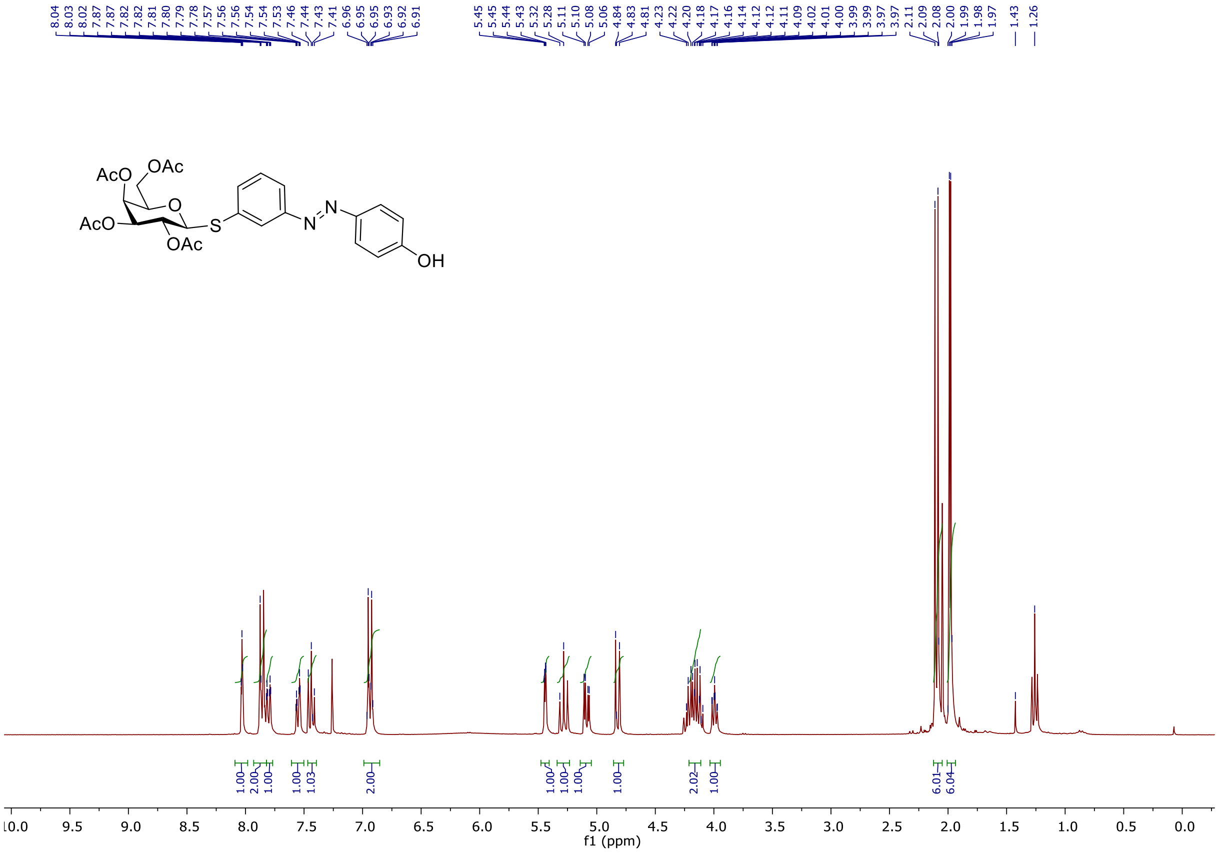

^{13}C{^{1}H} NMR of compound *E*-18 in $CDCl_3$ at 75 MHz

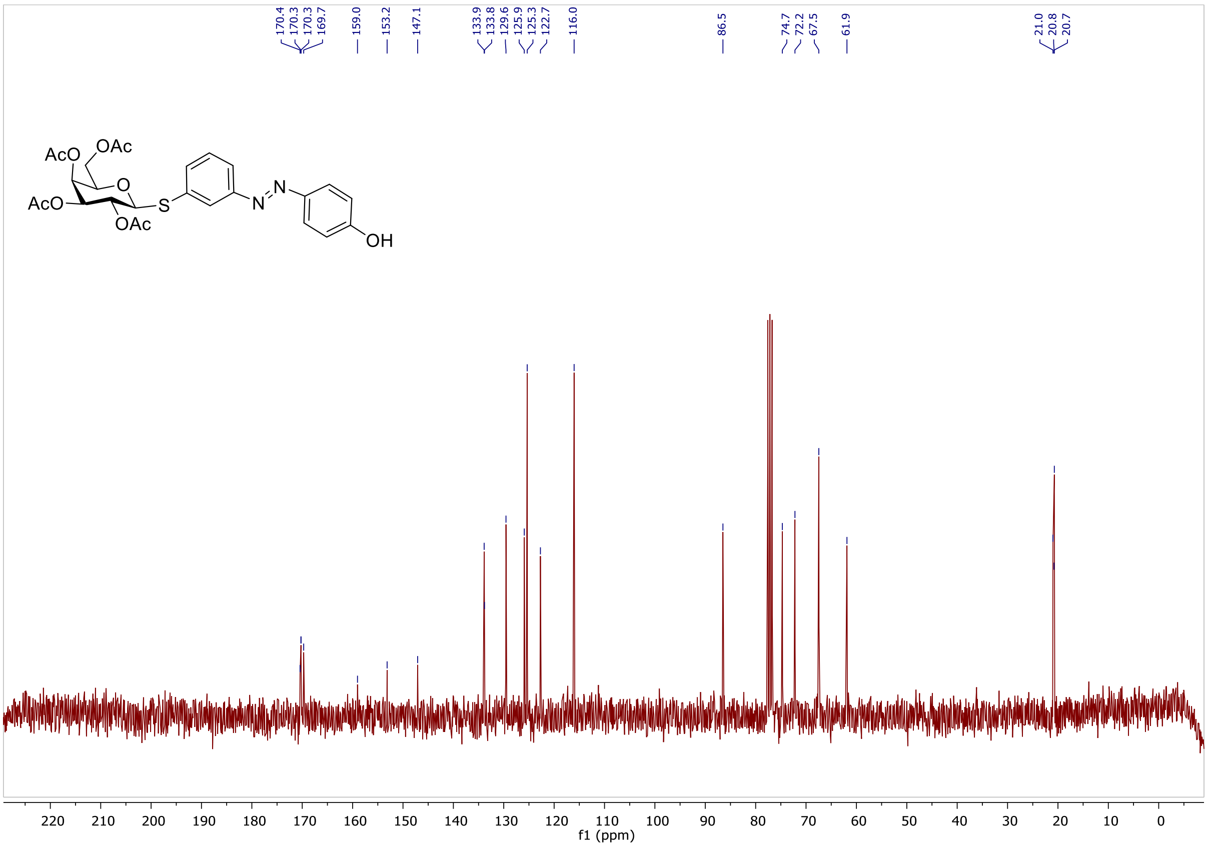

^{1}H NMR of compound *E*-19 in CD_3OD at 300 MHz

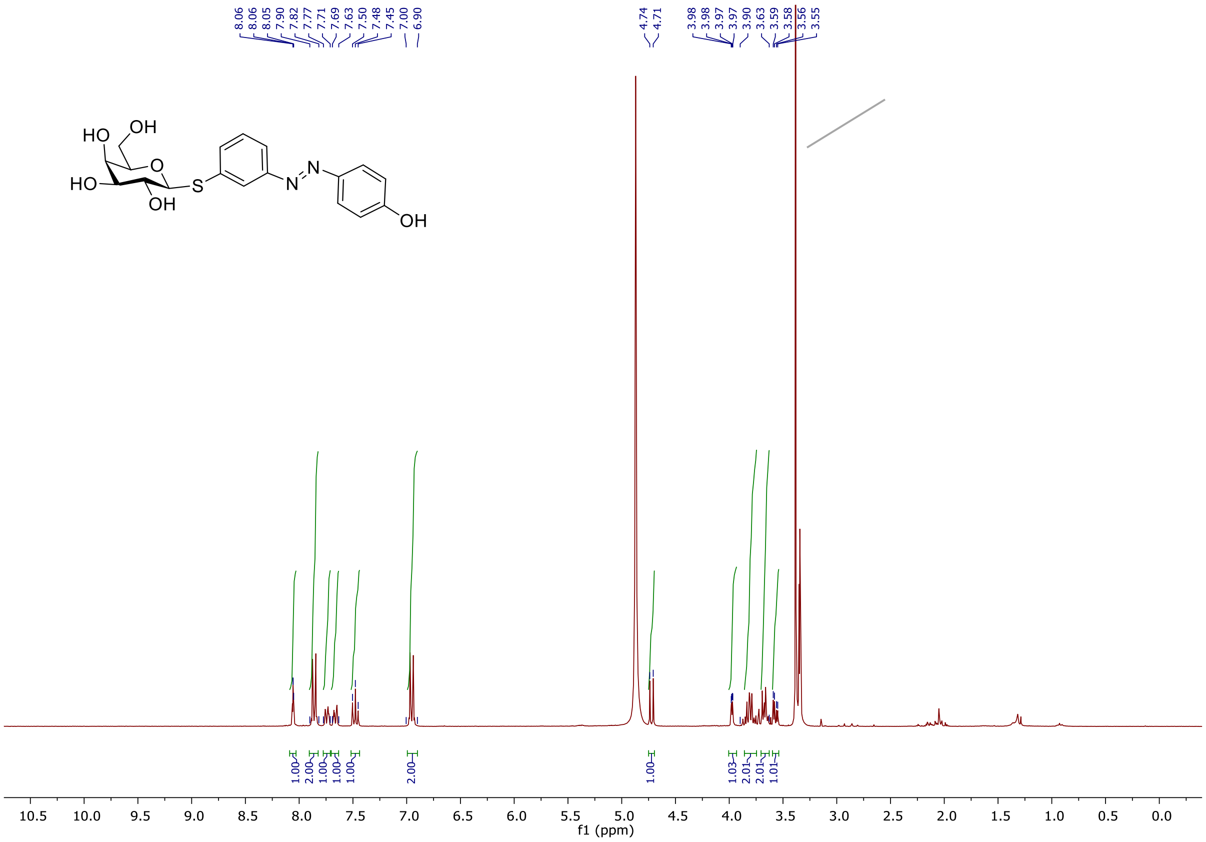

$^{13}C\{^{1}H\}$ NMR of compound *E*-19 in acetone-d_6 at 75 MHz

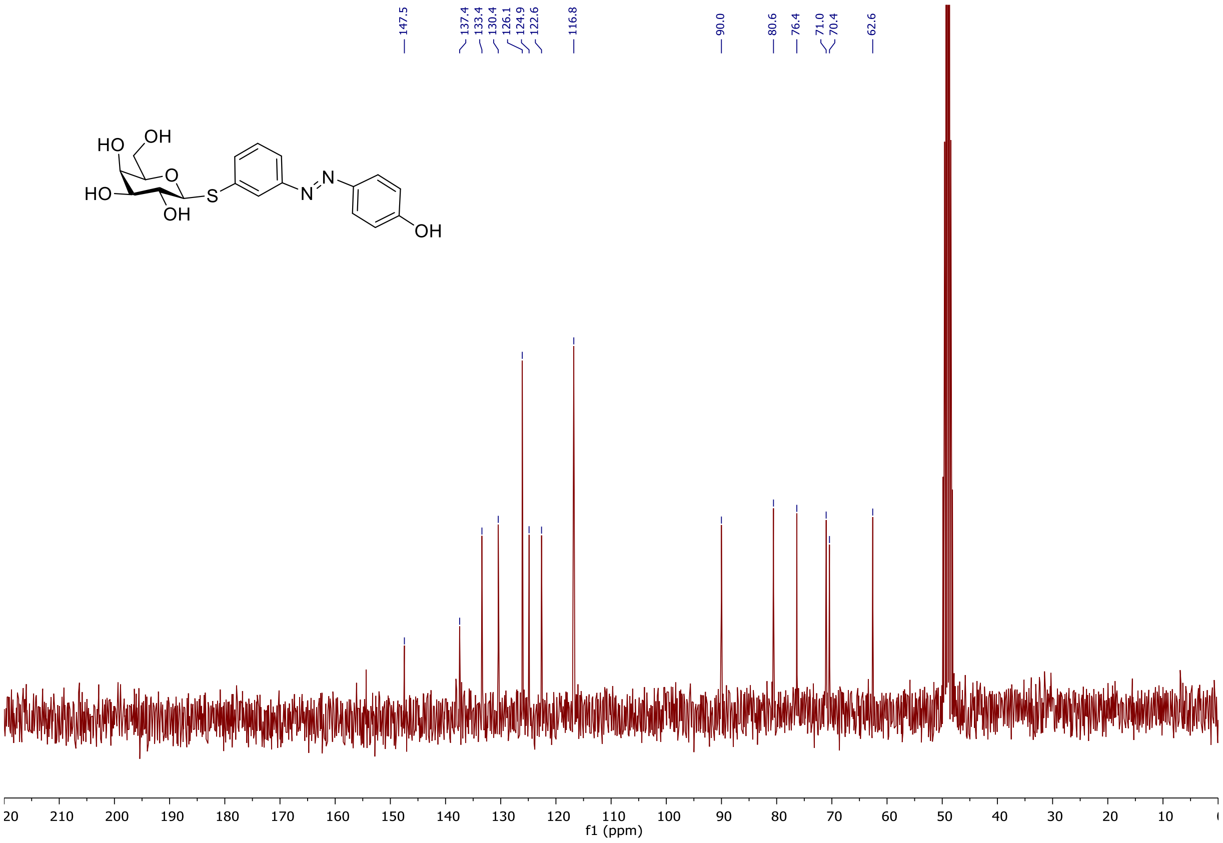

^{1}H NMR of compound *E*-20 in CD3OD at 400 MHz

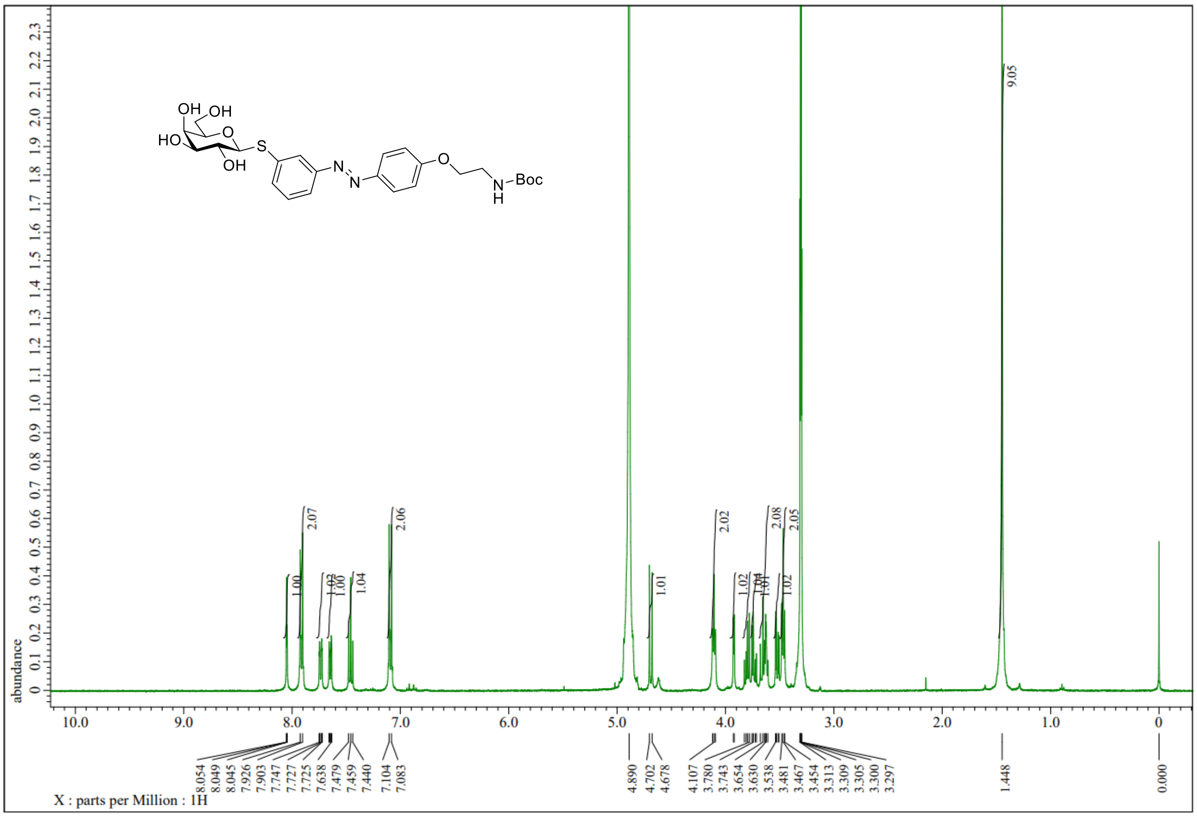

Enlarged ^{1}H NMR of compound *E*-20 in CD3OD at 400 MHz

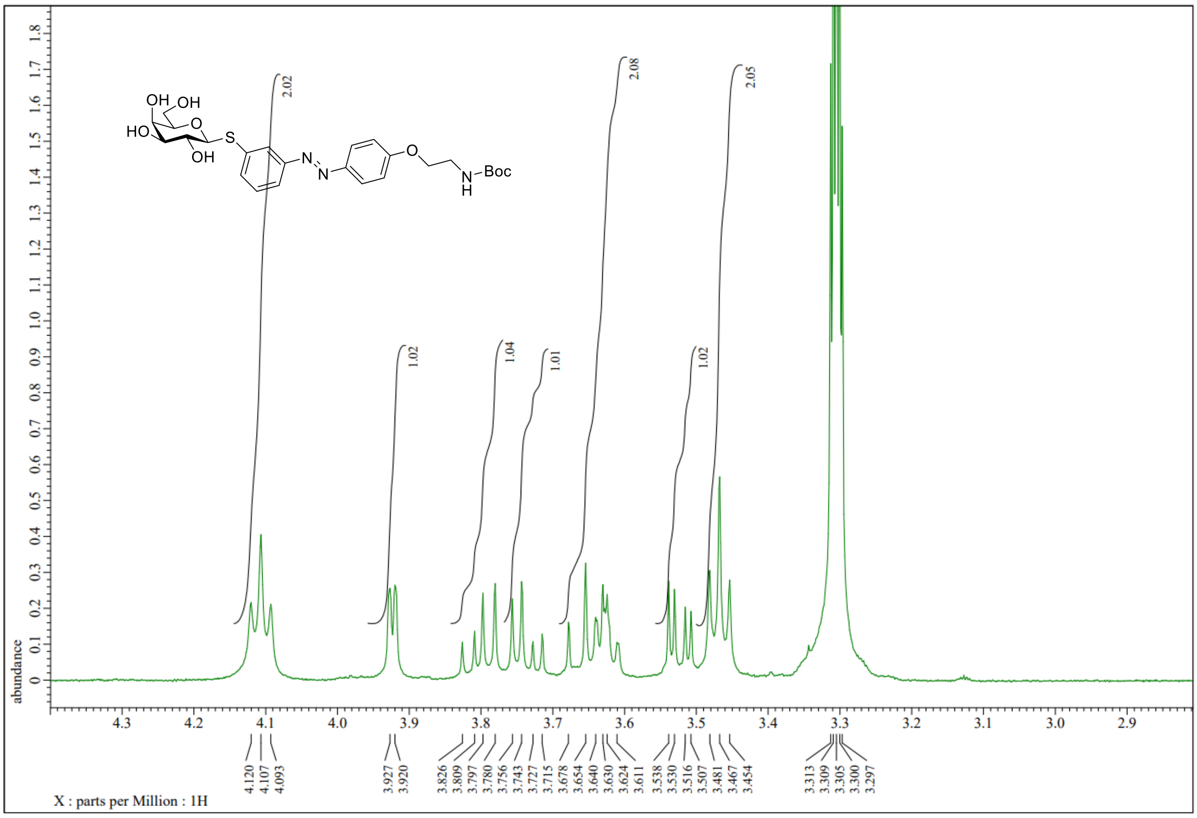

$^{13}C\{^{1}H\}$ NMR of compound *E*-20 in CD_3OD at 100 MHz

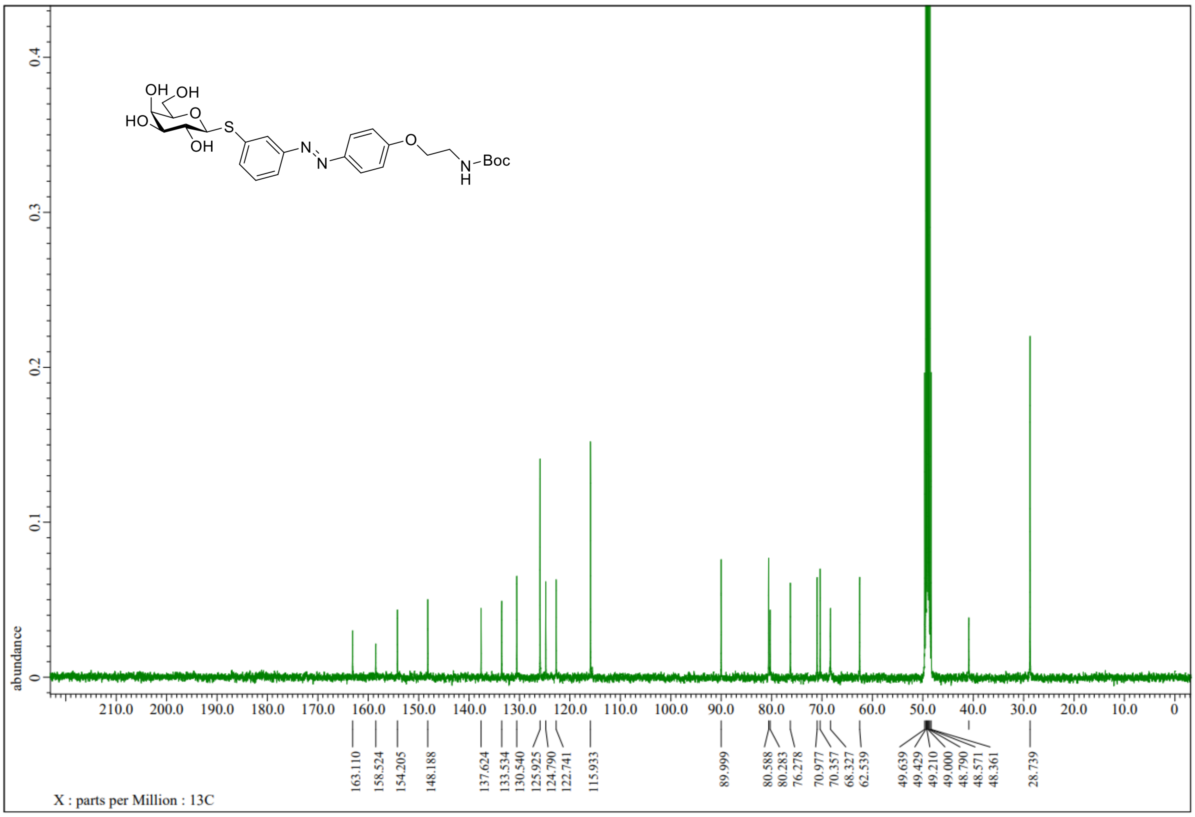

^{1}H NMR of compound *E*-22 in $CDCl_3$ at 300 MHz

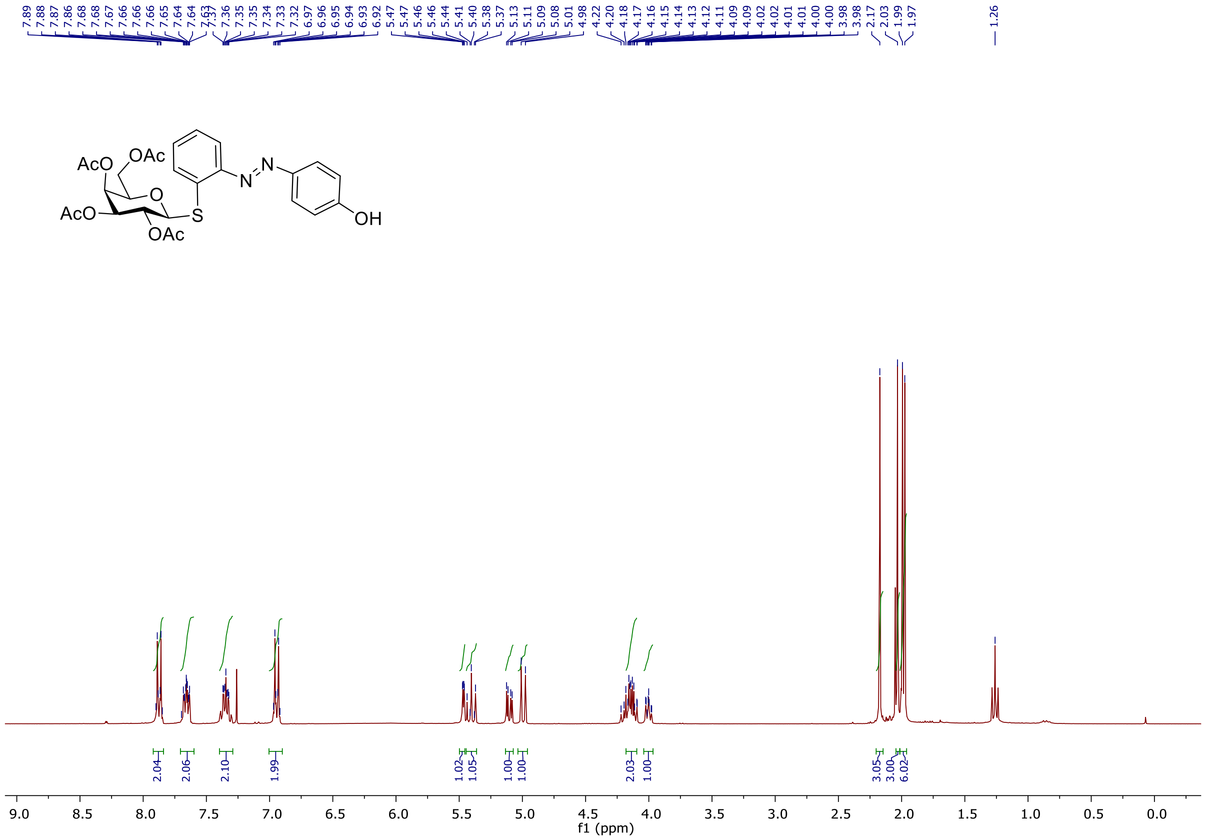

^{13}C{^{1}H} NMR of compound *E*-22 in $CDCl_3$ at 75 MHz

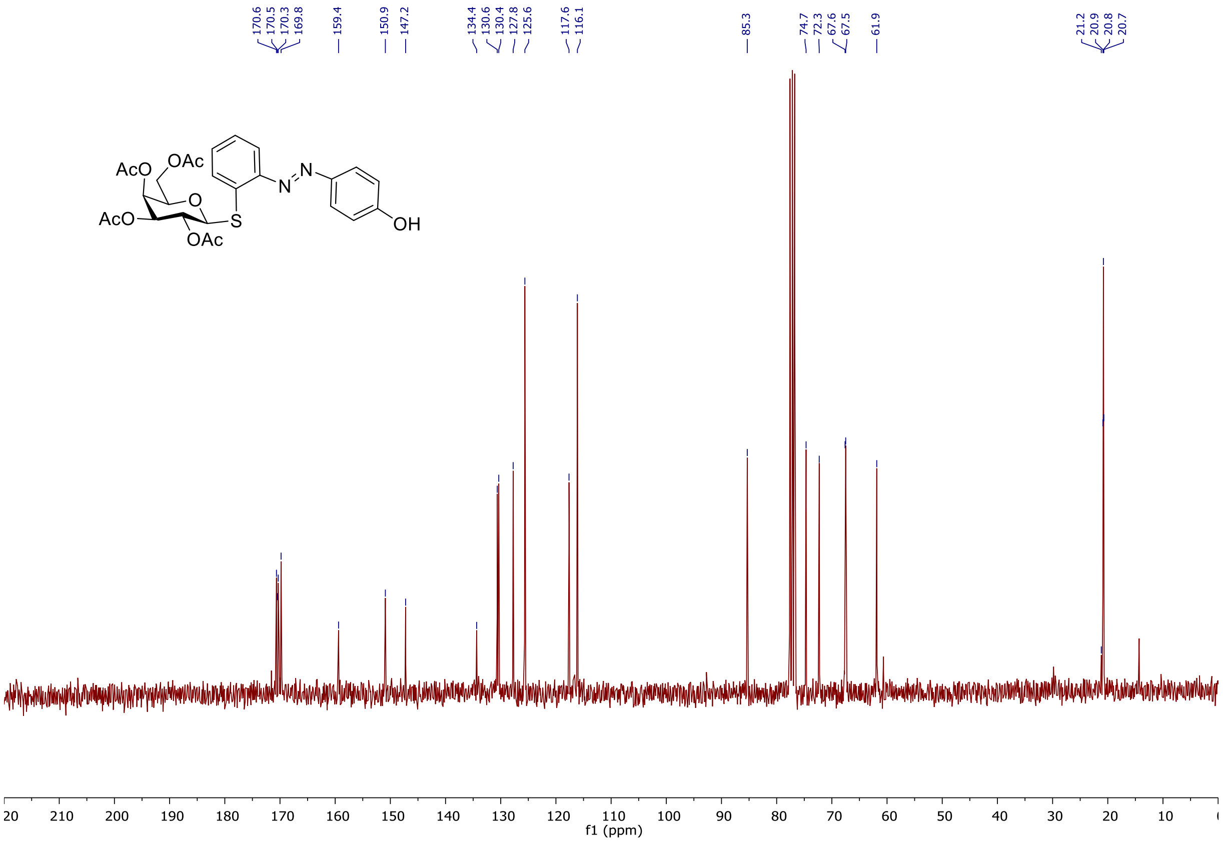

^{1}H NMR of compound *E*-23 in CD_3OD at 400 MHz

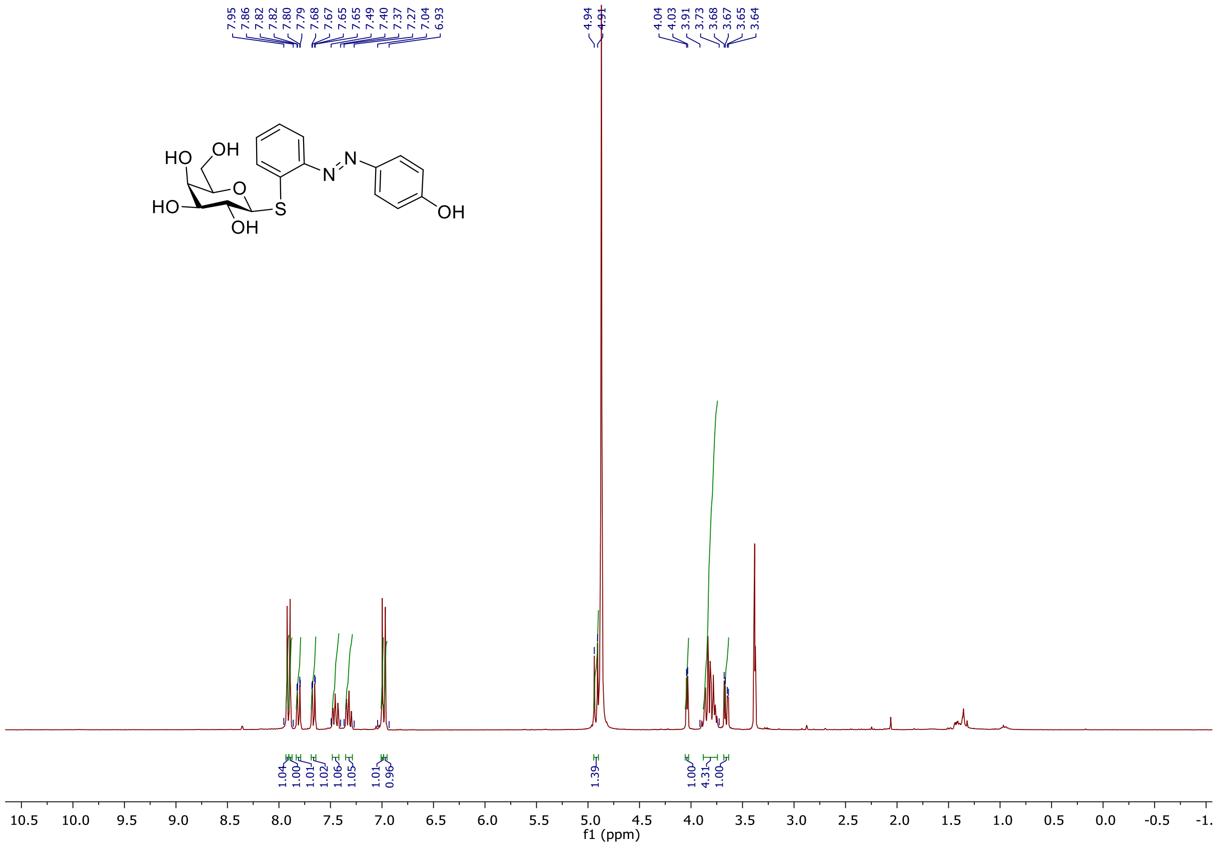

$^{13}C\{^{1}H\}$ NMR of compound *E*-23 in CD_3OD at 75 MHz

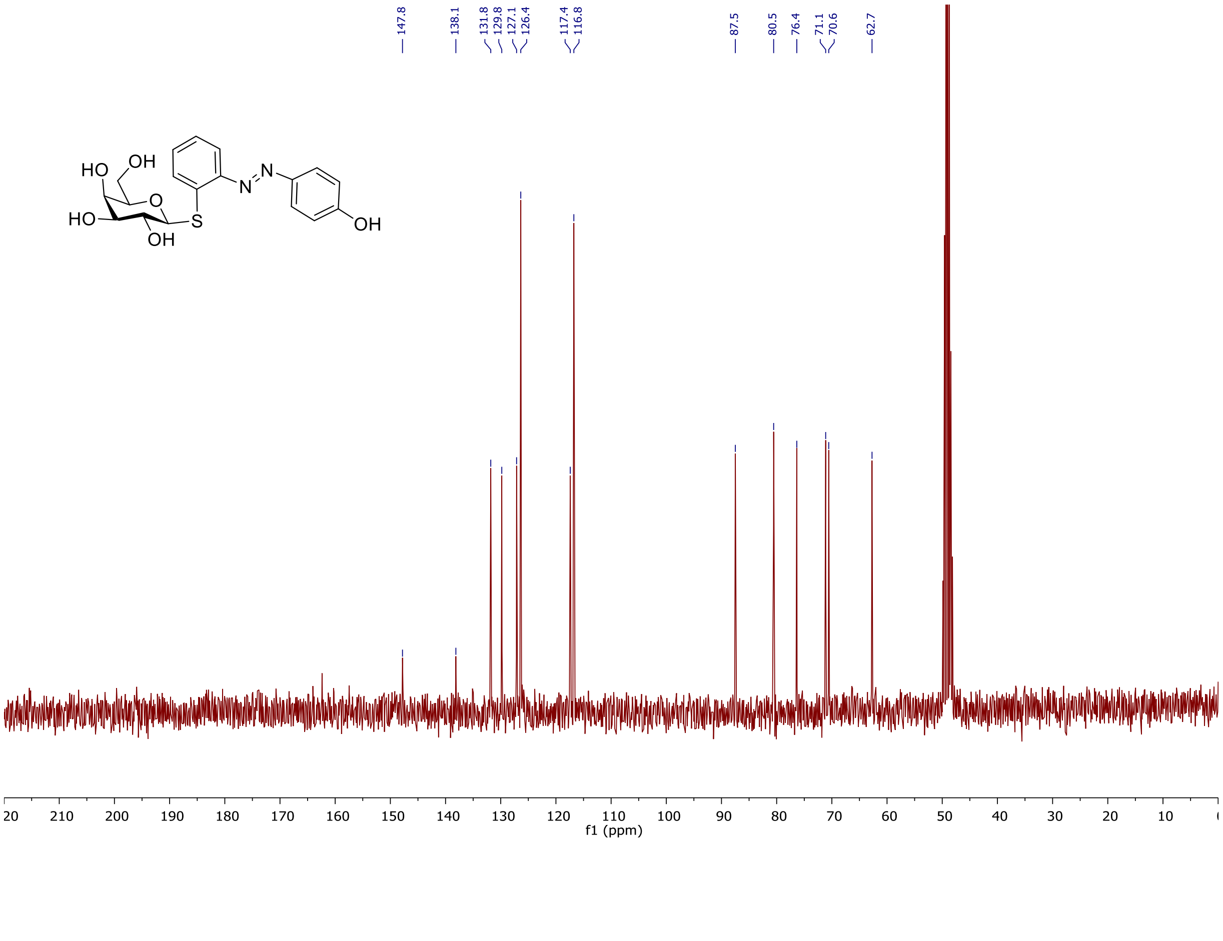

^{1}H NMR of compound *E*-24 in CD_3OD at 400 MHz

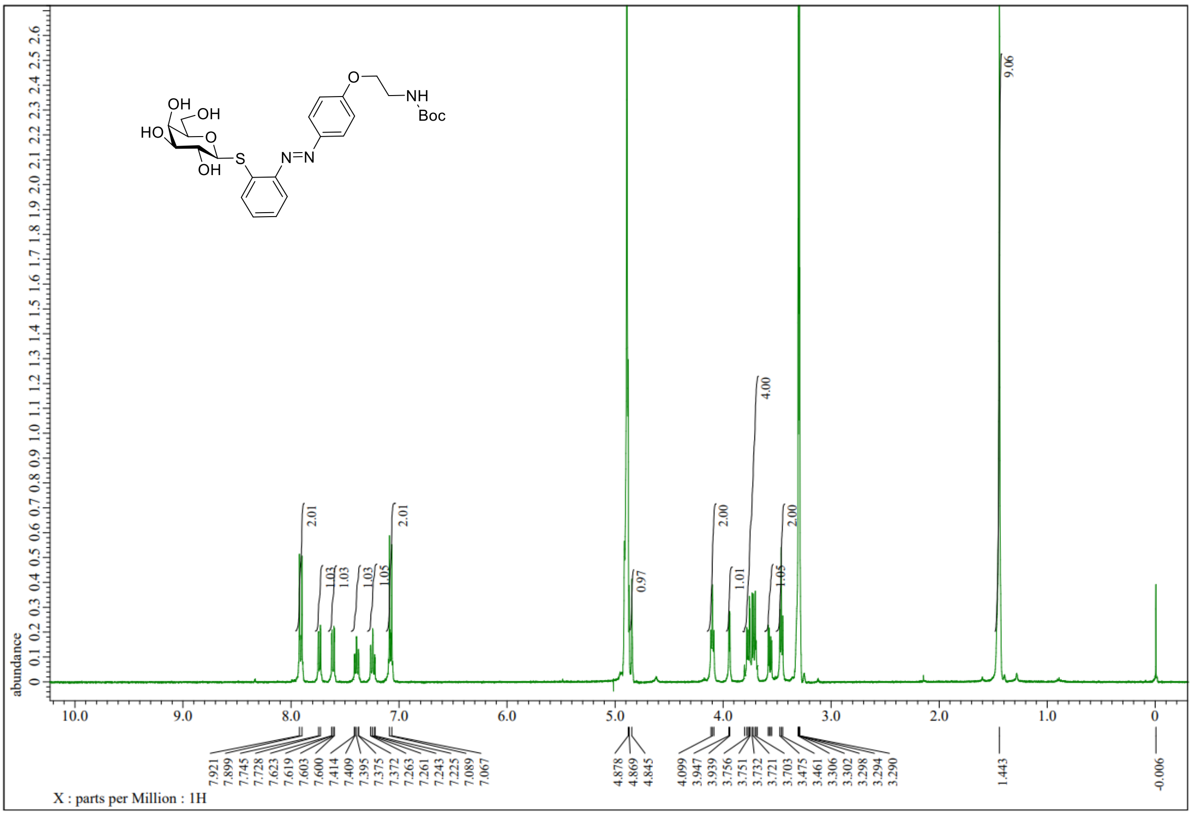

Enlarged ^{1}H NMR of compound *E*-24 in CD_3OD at 400 MHz

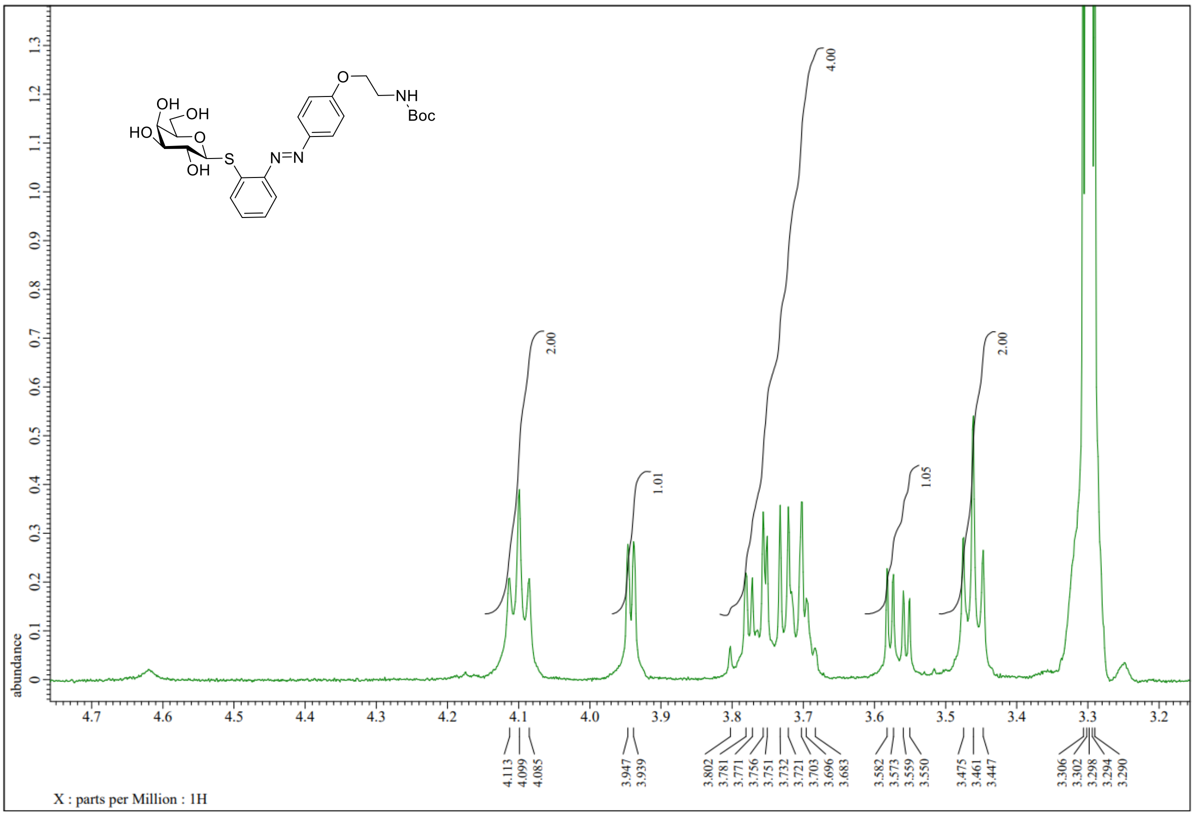

$^{13}C\{^{1}H\}$ NMR of compound *E*-24 in CD_3OD at 100 MHz

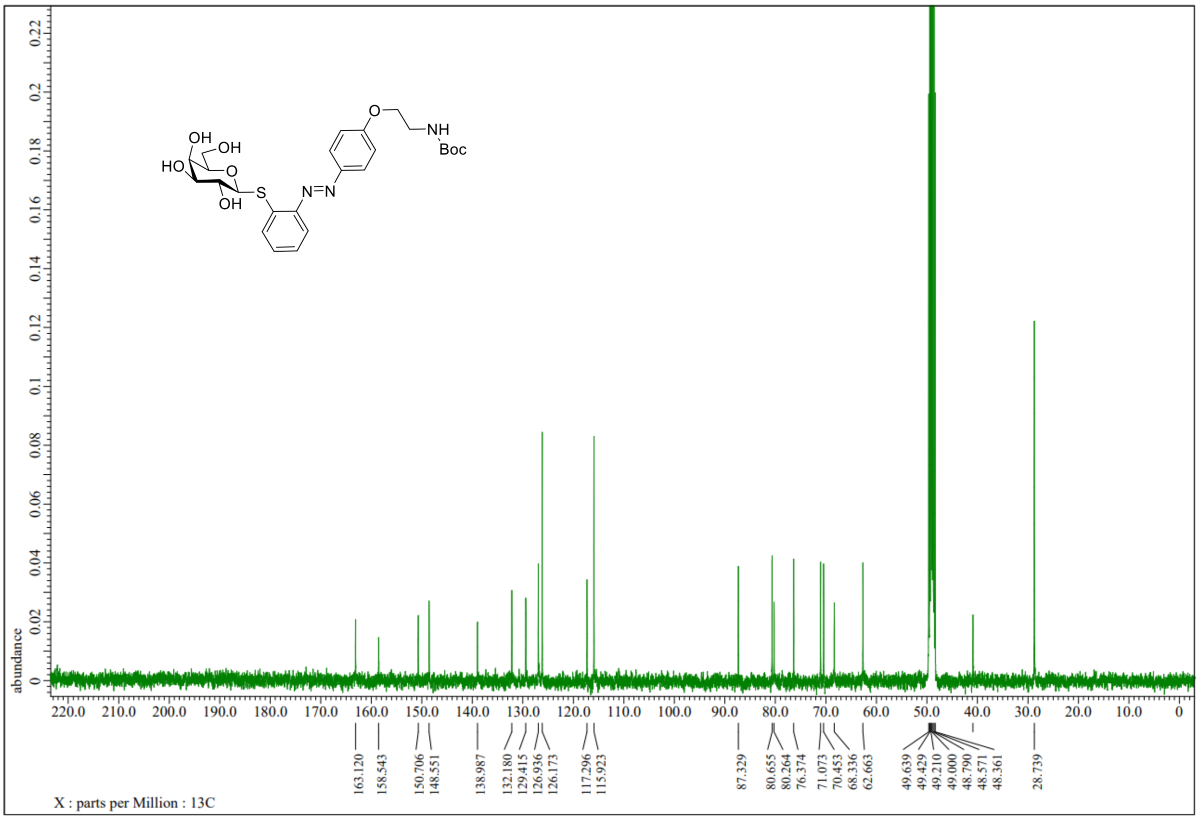

Supplement: File 1 — Compound characterization, detailed photochemical and photophysical procedures and copies of spectra. [file Beilstein_J_Org_Chem-20-1486-s001.pdf]
